# MetGENE: gene-centric metabolomics information retrieval tool

**DOI:** 10.1093/gigascience/giad089

**Published:** 2023-11-20

**Authors:** Sumana Srinivasan, Mano R Maurya, Srinivasan Ramachandran, Eoin Fahy, Shankar Subramaniam

**Affiliations:** University of California San Diego, Department of Bioengineering, 9500 Gilman Dr, La Jolla, CA 92093, United States; University of California San Diego, Department of Bioengineering, 9500 Gilman Dr, La Jolla, CA 92093, United States; University of California San Diego, Department of Bioengineering, 9500 Gilman Dr, La Jolla, CA 92093, United States; University of California San Diego, Department of Bioengineering, 9500 Gilman Dr, La Jolla, CA 92093, United States; University of California San Diego, Department of Bioengineering, 9500 Gilman Dr, La Jolla, CA 92093, United States; University of California San Diego, San Diego Supercomputer Center, Department of Computer Science and Engineering, Department of Cellular and Molecular Medicine, 9500 Gilman Drive, La Jolla, CA 92093, United States

**Keywords:** metabolomics workbench, gene-centric, data aggregator, web application

## Abstract

**Background:**

Biomedical research often involves contextual integration of multimodal and multiomic data in search of mechanisms for improved diagnosis, treatment, and monitoring. Researchers need to access information from diverse sources, comprising data in various and sometimes incongruent formats. The downstream processing of the data to decipher mechanisms by reconstructing networks and developing quantitative models warrants considerable effort.

**Results:**

MetGENE is a knowledge-based, gene-centric data aggregator that hierarchically retrieves information about the gene(s), their related pathway(s), reaction(s), metabolite(s), and metabolomic studies from standard data repositories under one dashboard to enable ease of access through centralization of relevant information. We note that MetGENE focuses only on those genes that encode for proteins directly associated with metabolites. All other gene–metabolite associations are beyond the current scope of MetGENE. Further, the information can be contextualized by filtering by species, anatomy (tissue), and condition (disease or phenotype).

**Conclusions:**

MetGENE is an open-source tool that aggregates metabolite information for a given gene(s) and presents them in different computable formats (e.g., JSON) for further integration with other omics studies. MetGENE is available at https://bdcw.org/MetGENE/index.php.

Key Points

- Knowledge-based data aggregator.
- Gene-centric query.
- Metabolomics Workbench studies.

## Introduction

Recent advances in high-throughput technologies have led to many high-resolution multiomic measurements available to biomedical researchers. However, obtaining biological insights remains challenging since considerable effort is required to find and access data from diverse sources, deal with diverse and sometimes incomplete data formats, and tease out the connections within those high-dimensional datasets. This has led to an initiative by the US National Institutes of Health (NIH) called the Common Fund Data Ecosystem (CFDE), which aims to provide a single portal that makes data findable, accessible, interoperable, and reusable (FAIR) across the data repositories maintained by Data Coordination Centers (DCCs). Some examples of DCCs include the Metabolomics Workbench (MW), which is a national metabolomics data repository [1]; Genotype-Tissue Expression (GTEx) Project, a comprehensive resource to study tissue-specific gene expression and regulation [2]; and the Library of Integrated Network-Based Cellular Signatures (LINCS) with the goal of generating a large-scale and comprehensive catalog of perturbation–response signatures by utilizing a diverse collection of perturbations across many model systems and assay types [3]. The MW is a comprehensive resource hosting more than 2,000 curated metabolomics studies and provides an integrated environment for data analysis and visualization through a suite of tools and interfaces to facilitate gaining biological insights.

A gene is a fundamental unit of query in the multiomics data hierarchy. One of the goals of CFDE is to make every DCC support gene-centric querying within their repositories. Currently, the MW supports a limited capability to perform gene-centric queries on the studies. MetGENE was designed to bridge this gap and enhance the capability by allowing a user to specify a gene or a set of genes that code for the metabolic enzyme(s) as a search term and, in return, fetch the relevant information from sources like the Kyoto Encyclopedia of Genes and Genomes (KEGG) [4] and the MW. Given one or more genes, the MetGENE tool identifies associations between the gene(s) and the metabolites (biosynthesized or catabolized) through the reactions and pathways involving these metabolites. For each metabolite, studies containing the metabolite are identified from the MW. The results are organized as a gene landing page or a Dashboard, with all the information presented in a user-friendly manner to enable further analyses. There are many other ways a gene may have an “association” with a small-molecule metabolite, such as via gene regulation (e.g., Transcription Factor (TF)–target relationship) or its protein product, gene expression–metabolite association, protein–protein–metabolite association, and so on. However, in MetGENE, we only focus on those genes that encode for proteins directly associated with metabolites, namely, metabolic enzymes.

While other databases such as GeneCards [5] provides extensive gene-related information (although not in a readily accessible format for metabolites, despite the presence of hyperlinked ChEBI IDs), and MetaCyc [6] provides information on genes, their associated pathways, and reactions, to the best of our knowledge, there exists no other tool that establishes a link between a gene and its relevant metabolomic studies like MetGENE does. This is important for several reasons. Consider a scenario where a biomedical researcher aims to uncover therapeutic targets for a gene like *IDH1*, which plays a crucial role in the early stages of tumorigenesis across various cancers. Given its metabolic significance, the researcher may want to find specific metabolomic studies and their findings (e.g., statistical and biological insights) relevant to cancer. Currently, apart from conducting searches on the MW, the only viable option would entail painstakingly combing through scientific literature to locate relevant information. MetGENE simplifies this process by employing targeted anatomy and disease filters and seamlessly connects genes with the precise metabolomic studies of interest. This not only drastically reduces the time and effort required by users but also streamlines the exploration of relevant data on a single, accessible platform. Furthermore, MetGENE goes beyond offering information about individual genes; it extends its capabilities to cover entire gene sets in one seamless process. In scenarios where users identify a specific subset of genes within a particular context, MetGENE stands out by efficiently processing the entire list. This differs from the practice of fetching information individually, as commonly seen in platforms like GeneCards, MetaCyc, or KEGG.

## Methods

**Figure 1:**
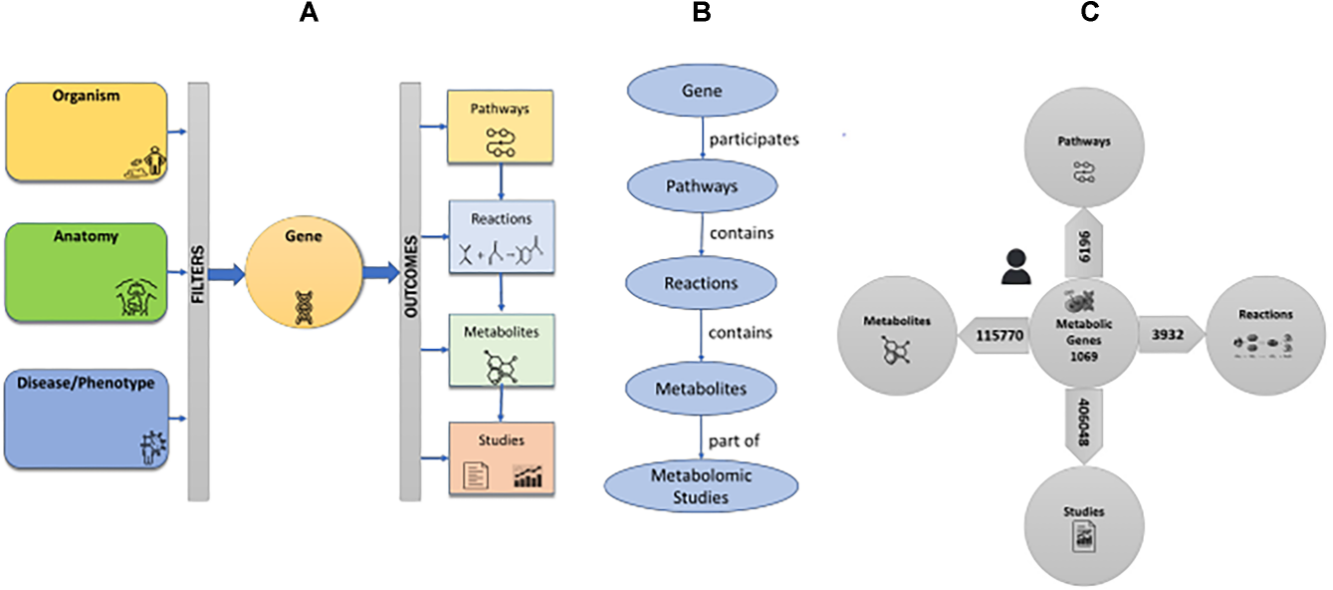
(A) Gene(s) search is contextualized by the organism (species). The gene-associated pathways, reactions, metabolites, and their corresponding metabolomic studies are reported as outcomes. Metabolites and Studies information can be filtered using anatomy (sample source), disease, or phenotype. (B) The knowledge graph underlying MetGENE. (C) The number of associations for each relation in MetGENE.

MetGENE (RRID:SCR_023402) is a hierarchical, knowledge-based gene-centric information retrieval tool. Given a gene or a set of genes as a search term, MetGENE returns entities associated with the gene(s)—namely, pathways, reactions, metabolites, and metabolomic studies in the MW, as shown in Fig. 1. MetGENE contextualizes the search by allowing the users to specify filters based on organism name, anatomy or tissue name (broadly, sample source), and disease/phenotype as a part of its query interface, as shown in Fig. 1A. A knowledge graph represents a network of entities, such as objects or concepts, and depicts their relationship. The knowledge graph that underlies information retrieval in MetGENE is depicted in Fig. 1B. Further, for the human species, the number of metabolic genes, gene–pathway, gene–reaction, gene–metabolite, and gene–metabolomic study associations found in the MW is enumerated in Fig. 1C.

**Figure 2:**
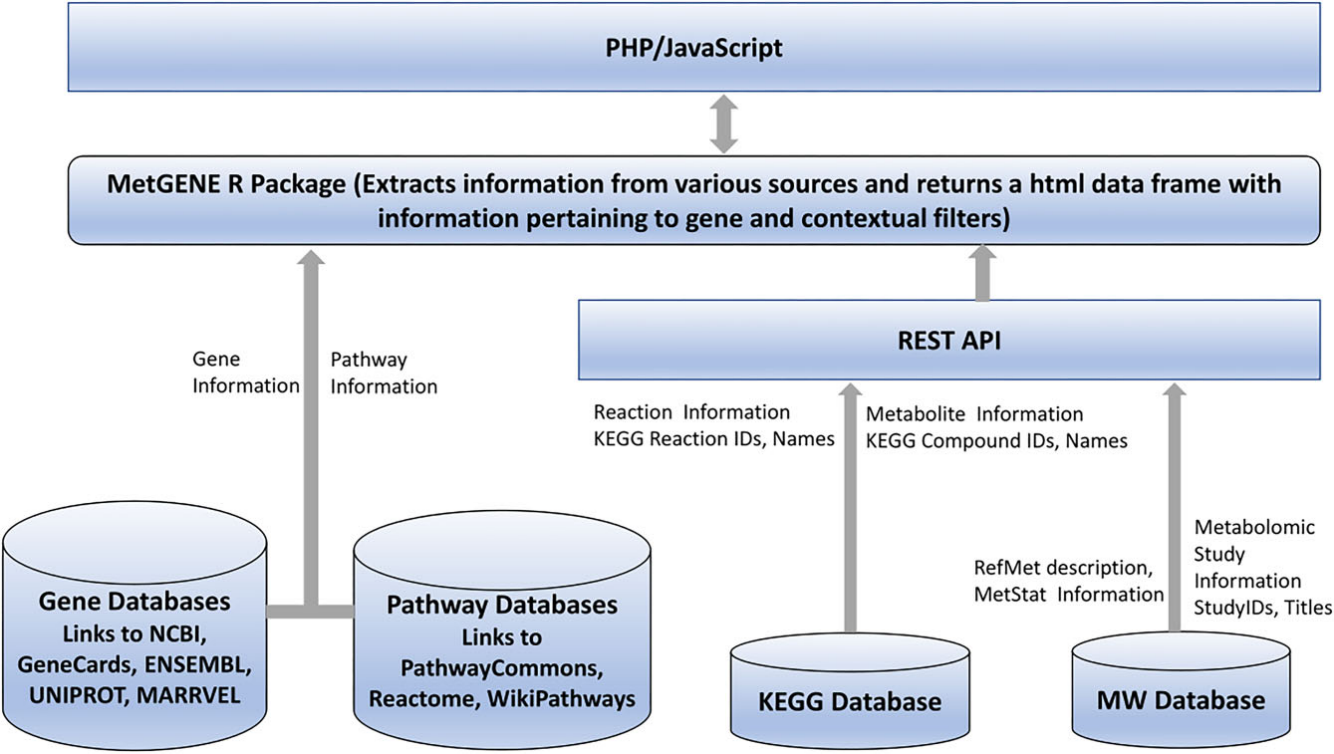
The architecture of MetGENE comprises server-side PHP and JavaScript interacting with R scripts that use REST APIs to extract information from KEGG and MW databases. The gene and pathway information links are generated for specific repositories.

MetGENE is designed as a web-based application using PHP and JavaScript as the front-end. The back-end contains R scripts with wrapper functions to retrieve information from various data repositories, such as KEGG [4] (for reaction and metabolite/compound IDs) and MW (for metabolite study IDs and RefMet names), as shown in Fig. 2. The KEGG database provides the KEGG REST API to access information from the KEGG database. For any given gene, MetGENE supports SYMBOL, ENTREZ ID, RefSeq, UniProt, Ensembl, and ALIAS (SYMBOL_OR_ALIAS) formats and converts IDs using an in-house Gene ID Conversion Tool (GICT). The GICT uses R Bioconductor packages, org.Xy.eg.db (e.g., org.Hs.eg.db for human), and NCBI gene_info table to convert the gene IDs. If the ID type for the input term is SYMBOL_OR_ALIAS, then the term is first searched in SYMBOL. If not found, then it is searched in ALIAS. Given the 3-letter KEGG organism code and the ENTREZ gene ID (of the input gene term from the GICT), the R KEGGREST API provides a way to access all the information, such as pathway IDs, reaction IDs, and compound (metabolites) IDs as a data frame object, which is parsed further to display relevant information. The KEGG compound ID, along with the filter information pertaining to the species, anatomy, and disease/phenotype, is used to extract information such as RefMet names and study IDs using the MW REST API. RefMet names provide a standardized reference nomenclature for both discrete metabolite structures and metabolite species identified in metabolomic experiments. This is an essential prerequisite for comparing and contrasting metabolite data across different experiments and studies. For efficiency and to speed up the display, MetGENE caches the number of pathways, reactions, metabolites, and studies associated with a particular gene, which is updated weekly to accommodate new studies being deposited into the MW.

MetGENE maintains session variables for species ID and organism name, ENTREZ gene ID and gene symbol, anatomy, disease/phenotype terms, and the previous values of these terms to enable server-side caching of pages and thus avoid unnecessary and time-consuming fetching of data across the network. The MetGENE source code is available on GitHub for download. For programmatic ease of access, we provide REST APIs that output each information table displayed in JSON or CSV formats. The REST APIs are developed using Smart/Open API format [7].

## Results

In this section, we describe the user experience starting from the MetGENE Query Page and ending with the MetGENE Studies Page containing metabolomic studies corresponding to the gene in the MW, incorporating various intermediate views of interest based on the knowledge graph described earlier.

### MetGENE query interface

**Figure 3:**
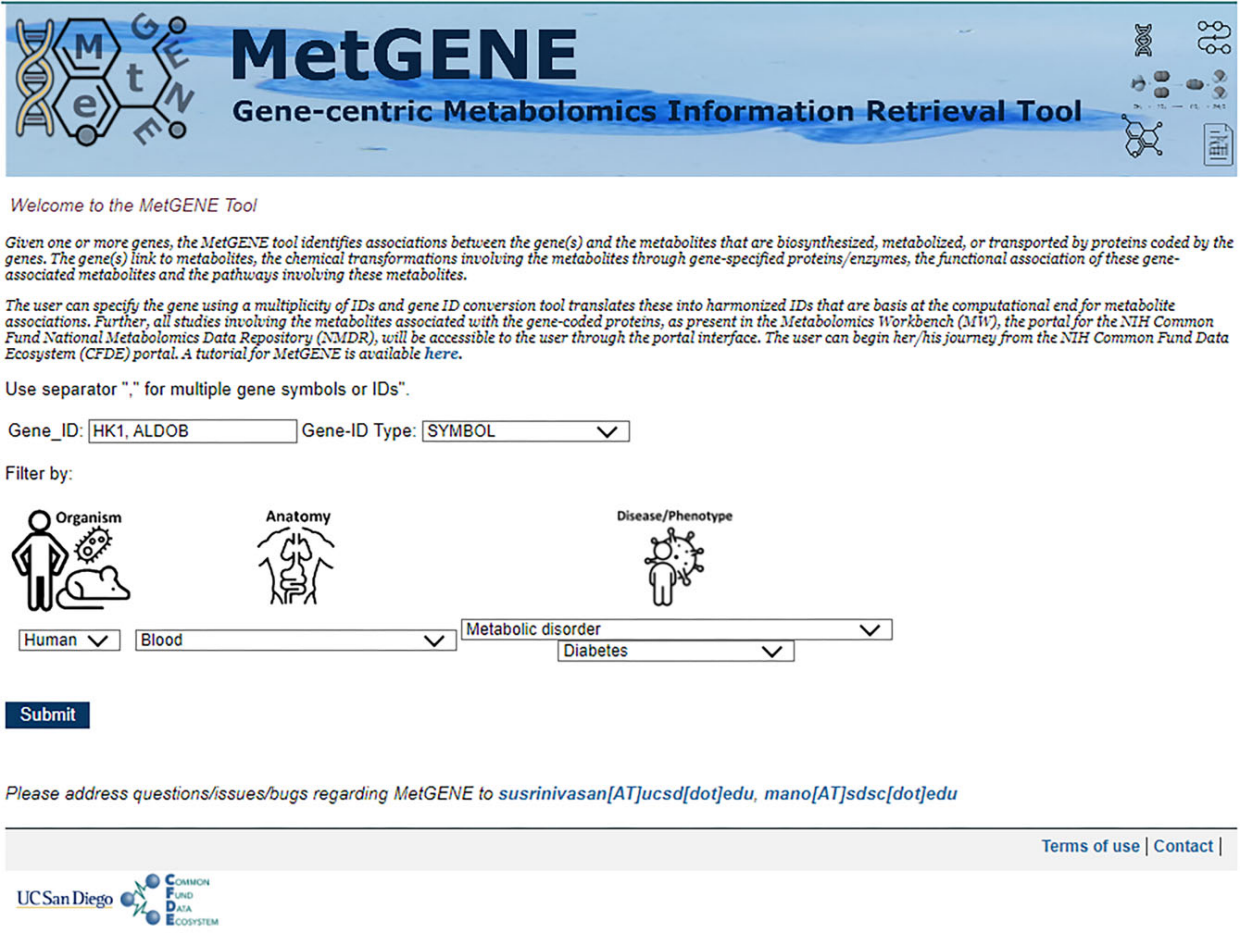
MetGENE Query page with the organism, anatomy (sample source), and disease/phenotype-specific filters.

**Figure 4:**
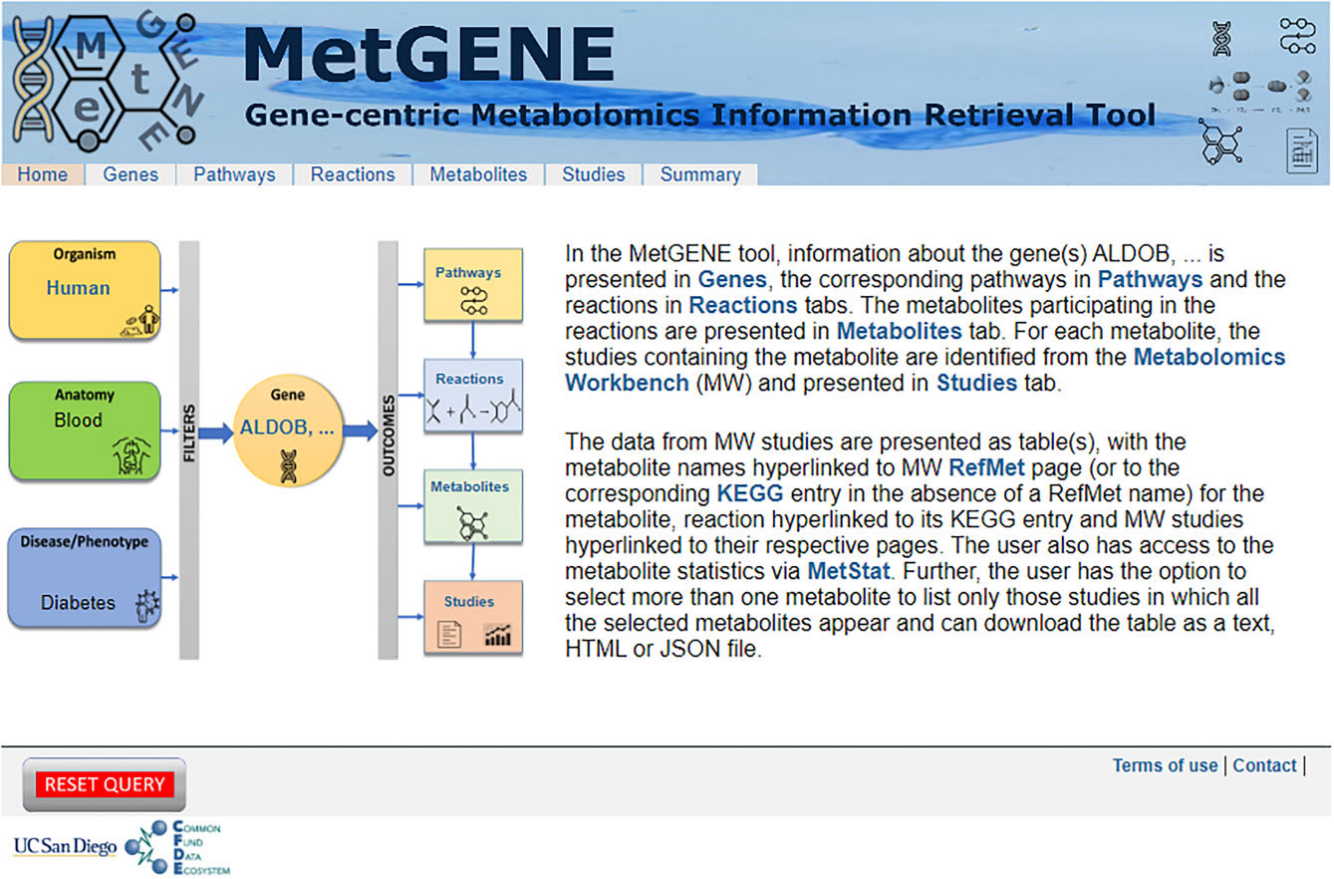
MetGENE landing page with context-sensitive display and access to Gene, Pathway, Reactions, Metabolites, Study, and Summary information.

The user can input the gene information as a gene ID in any one of the formats described in the previous section. The format of the query page is as shown in Fig. 3. The gene search input is validated on the client side to allow only alphanumeric symbols. Invalid gene IDs are recognized, and appropriate error messages are displayed. MetGENE uses terms (e.g., Human, Mouse) for taxonomy filtering as per the NCBI taxonomy database [8]. Currently, MetGENE supports human (*Homo sapiens*), mouse (*Mus musculus*), and rat (*Rattus norvegicus*) species, and we plan to add *Escherichia coli* (K12), *Caenorhabditis elegans*, common fruit fly (*Drosophila melanogaster*), and mosquito (*Anopheles gambiae*) in the near future. For filtering the information on metabolites and studies by anatomy/tissue (e.g., Liver, Blood) and disease/phenotype (e.g., Diabetes, Fatty liver disease), terms from the MW are used. Internally, the MW database records disease and phenotype under the metadata field, *disease*. Hence, the phenotype is searched as a disease term within the MW. The JSON files for each filter/category are curated and updated regularly and used to generate a pull-down menu. For the disease/phenotype filter, a 2-step selection menu with slim (or disease class) terms in the first level and fine-grained terms in the second level is used for ease of presenting the options to the user. The user inputs from this page (main landing page) are submitted as a form, and a second page for MetGENE (as shown in Fig. 4) is populated with the context-specific filtering terms. The second page comprises tabs for the search term–associated entities, “Genes,” “Pathways,” “Reactions,” “Metabolites,” “Studies,” and “Summary.” The Summary tab displays the total number of pathways, reactions, metabolites, and studies corresponding to each gene in the query. As MetGENE supports only those genes that encode proteins directly associated with metabolites, a warning is issued if the queried gene or a set of queried genes does not encode for such proteins.

### Gene and pathway information pages

**Figure 5:**
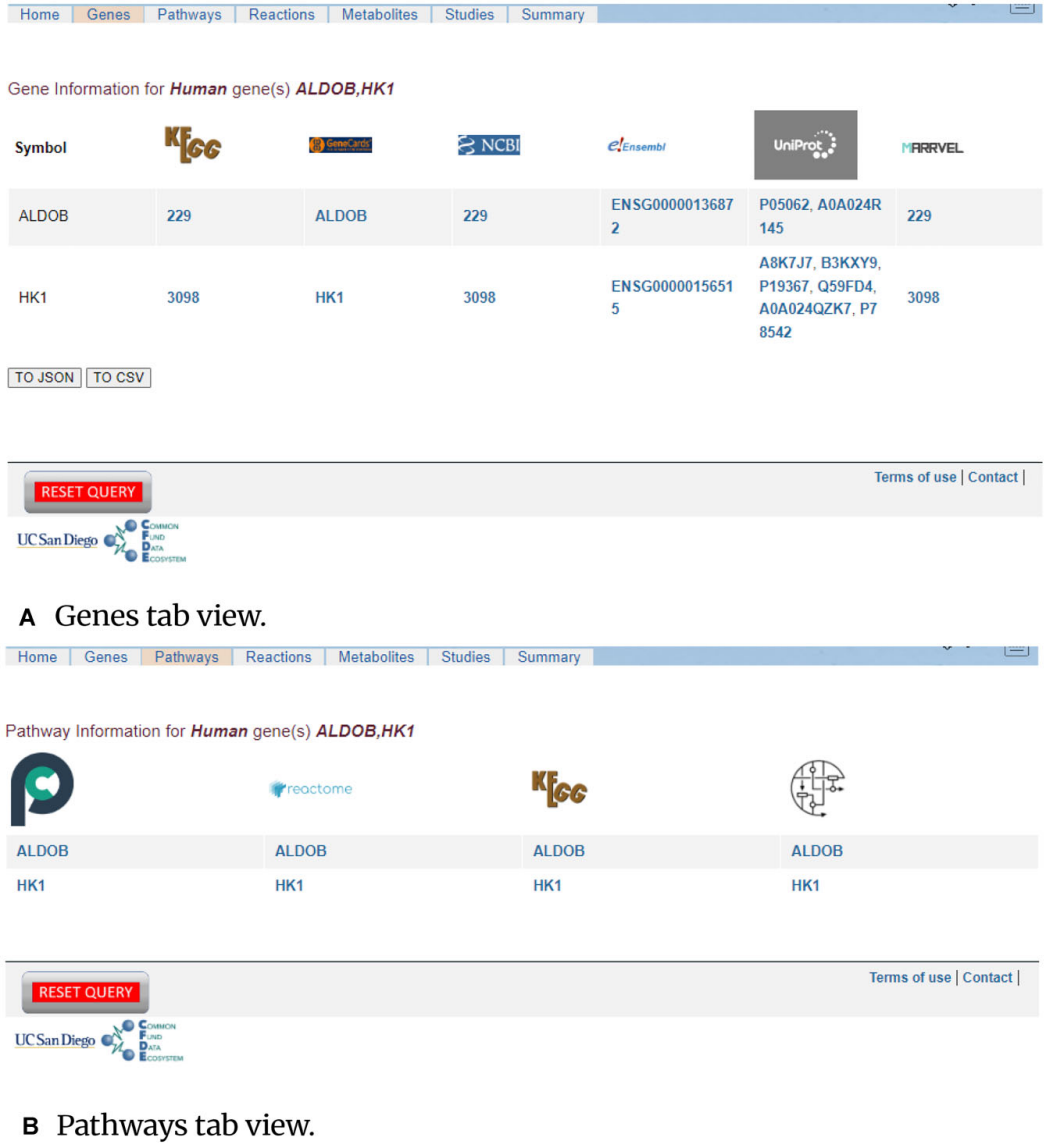
(A) MetGENE gene information page comprising Gene IDs in various formats corresponding to the searched gene(s) hyperlinked to various online resources. (B) MetGENE pathway information page comprising Gene IDs hyperlinked to various pathway resources.

The gene information page shown in Fig. 5A presents gene IDs in different formats hyperlinked to the corresponding webpages pointing to repositories such as KEGG [4], GeneCards [5], NCBI [9], Ensembl [10], UniProt [11], and Marrvel [12]. The URLs to these repositories for the specific genes are constructed based on their base URLs and the respective supported gene ID types. This information is obtained from the REST API of the GICT and converted from JSON to an HTML table format for display purposes.

The pathway information page (Fig. 5B) displays gene symbols hyperlinked with species and gene ID or symbol information as appropriate to various well-maintained pathway databases such as Pathway Commons [13], Reactome [14], KEGG [4], and Wikipathways [15]. MetGENE provides context-specific ease of access to these online resources.

### Reaction and metabolite information pages

**Figure 6:**
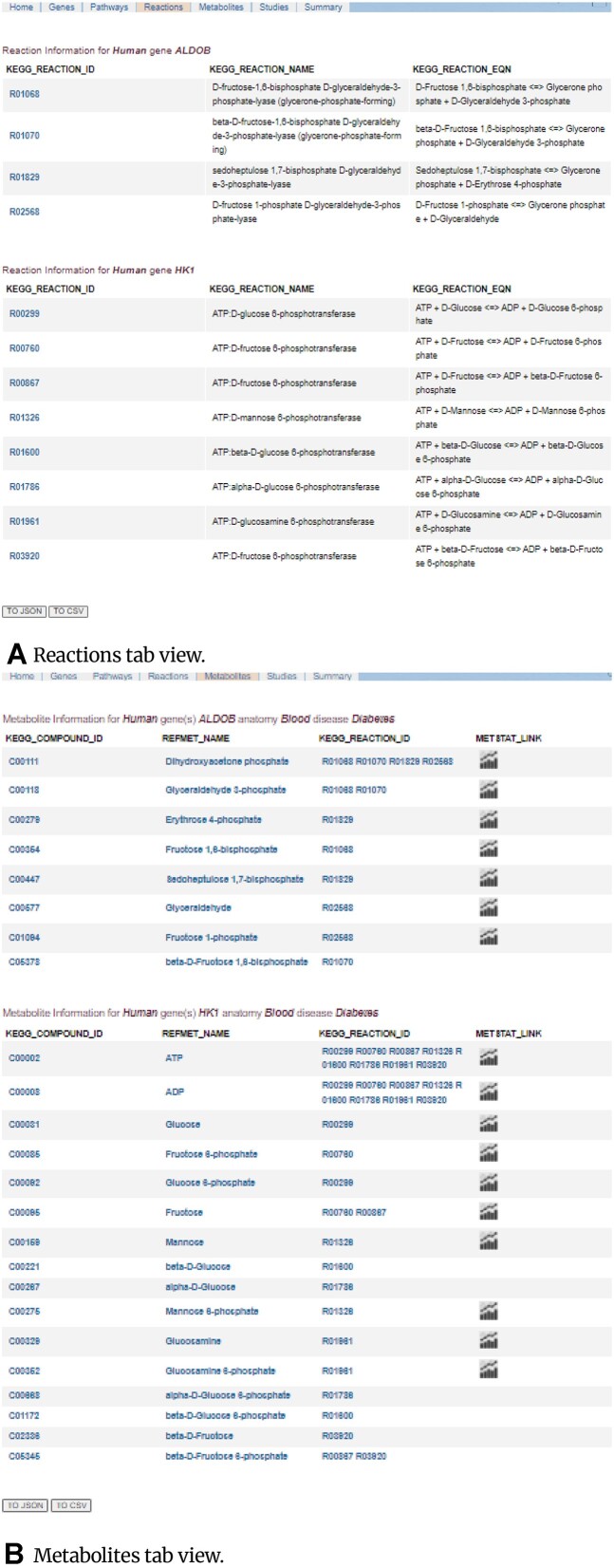
(A) MetGENE reaction information page comprising KEGG Reaction IDs hyperlinked to KEGG reaction information page and reaction descriptions in a tabular format. (B) MetGENE metabolite information page comprises KEGG compound IDs, MW RefMet names, and reaction IDs in which a metabolite participates in, hyperlinked to the KEGG reaction information page and MetStat link for the metabolite in a tabular format.

The KEGG database provides the KEGG REST API to access information from the KEGG database. Given the organism code and the gene ID, the R KEGGREST API provides a way to access all the information, such as pathway IDs, reaction IDs, reaction names, reaction equations, and compound (metabolites) IDs, which are displayed in a tabular format. Fig. 6A depicts the reaction information tab corresponding to the metabolic gene(s) of interest in a table view (1 per gene) comprising reaction IDs hyperlinked to the corresponding KEGG reaction information page, reaction names, and the reaction equation.

In the metabolites information tab, as shown in Fig. 6B, a unique list of metabolites across all reactions corresponding to a given gene, along with their respective RefMet names (MW provides REST APIs to access the RefMet name corresponding to a KEGG compound ID) and KEGG reaction IDs of all the reactions the metabolite participates in, are displayed. Further, the MW MetStat link provides access to the statistics about the metabolite measured across various studies in the MW database, filtered by anatomy (sample source) and disease/phenotype. This tool generates a report for any given metabolite in the MW, comprising all the unique studies containing that metabolite and their median value of the relative standard deviation (RSD) across all those studies. The KEGG compound IDs that do not have RefMet names in the MW database display only the KEGG compound name and reaction IDs. The KEGG compound IDs are hyperlinked to their corresponding KEGG compound information pages. MetGENE allows users to download the tables directly from the displayed page in JSON or CSV formats for further analysis. The MW also provides REST APIs to access all the study IDs, titles, and RefMet names for a given KEGG compound ID in JSON, text, and HTML formats.

### MetGENE metabolomics study information page

**Figure 7:**
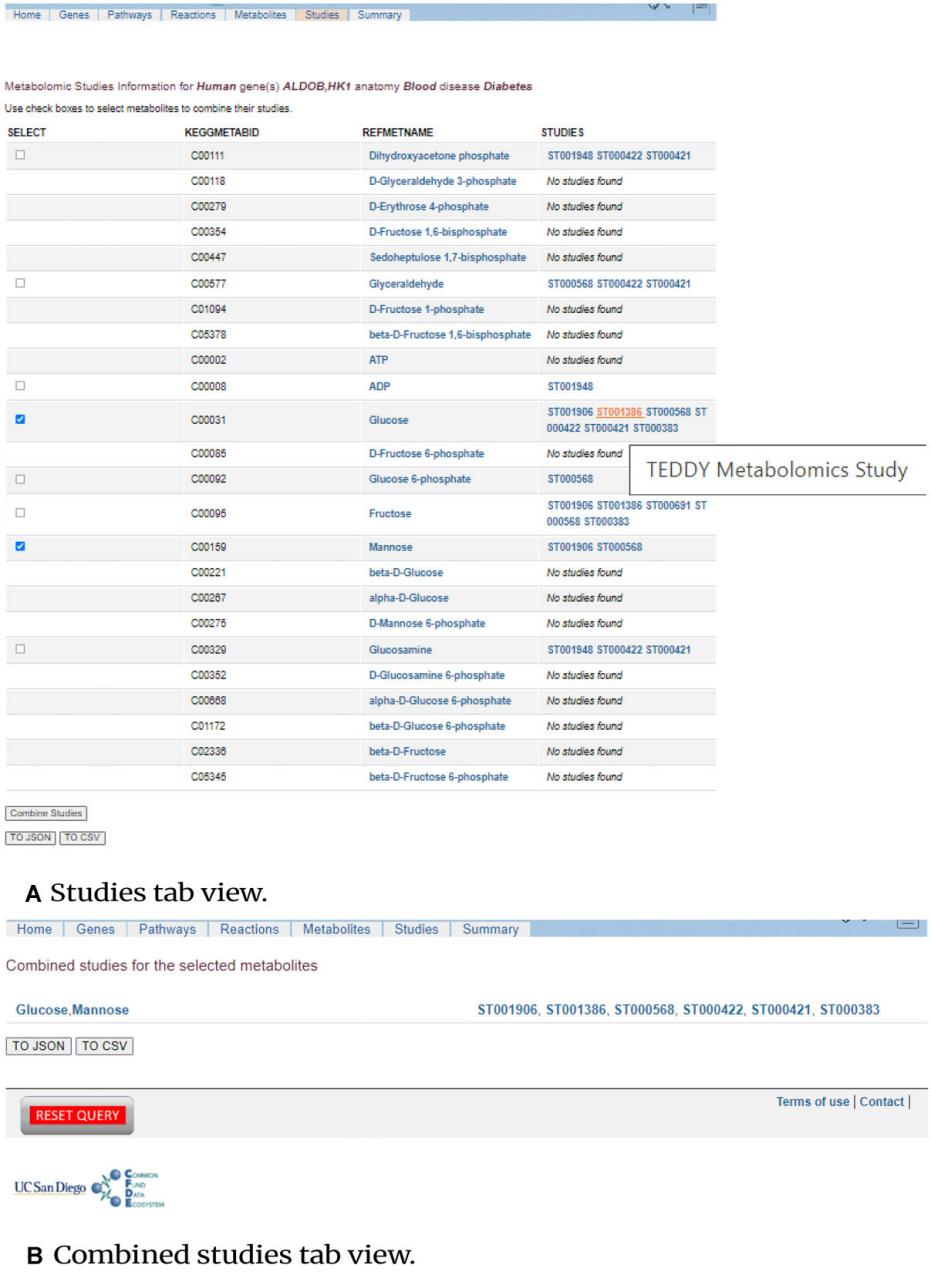
(A) MetGENE metabolomics studies information page comprises KEGG compound IDs, RefMet names, and MW study IDs corresponding to a metabolite in a tabular format. (B) MetGENE allows users to combine studies for a selected set of metabolites.

In the Studies page (as shown in Fig. 7A), a tabular view of the unique list of metabolites for the queried gene(s), their RefMet names hyperlinked to their corresponding description page in the MW, and a comma-separated list of study IDs in which the metabolite participates (with each study ID linked to its corresponding study description page in the MW) are presented. Further, a helpful text hover feature displaying the study title corresponding to a particular study ID is also provided to the user. MetGENE also allows users to select metabolites of interest and combine their studies for download and further analysis, as shown in Fig. 7B.

### MetGENE summary page

**Figure 8:**
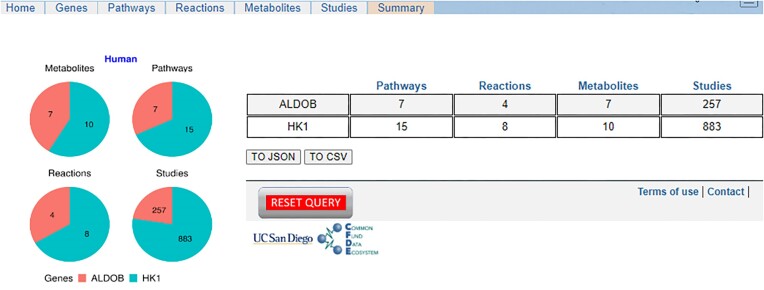
The Summary tab in MetGENE enumerates the total number of pathways, reactions, metabolites, and metabolomic studies corresponding to each queried gene.

In the Summary page (as shown in Fig. 8), MetGENE displays the total number of pathways, reactions, metabolites, and metabolomic studies for each queried gene and displays the information in a tabular format and a pie chart. This information is available for download both in JSON and CSV formats.

### Case study: exploring gene *PNPLA3* using MetGENE

Here, we demonstrate a use case that shows the utility of MetGENE as a 1-stop tool to obtain all metabolomic information associated with a gene(s) in a specific disease condition. The protein adiponutrin, encoded by the gene *PNPLA3*, is a multifunctional enzyme that belongs to the IPLA2/lipase family, which has both triacylglycerol lipase and acylglycerol O-acyltransferase activities. *PNPLA3* is predominantly expressed in adipocytes and liver cells. It regulates the development of adipocytes and the metabolism of fats (lipogenesis and lipolysis). Diseases associated with *PNPLA3* mutations include fatty liver disease and nonalcoholic steatohepatitis (NASH) [16–19].

To obtain information about a gene(s) and associated entities like enzymes, reactions, pathways, and existing metabolomic studies in MW for a given context (fatty liver disease in humans), a user needs to identify precise key terms, perform a search on the Internet, sift through the results to identify literature about various metabolomic studies, and download the studies to perform downstream analyses. These steps are sometimes time-consuming and misleading, depending on the search terms’ specificity. However, with MetGENE, the user can specify a gene ID(s) in any popular format and apply the filters on organism, anatomy, and disease/phenotype to obtain genes, pathways, reactions, metabolites, and study information consolidated from various data resources at one go. Supplementary Figure S1 shows the Gene tab for the *PNPLA3* gene. Links to various online resources (GeneCards, KEGG, NCBI, Uniprot, and Marrvel) with genomic, transcriptomic, proteomic, clinical, and functional information (via GeneCards link); module and pathway information (via KEGG); transcript and region information (via NCBI link); sequence, variant, and gene expression information (via Ensembl link); protein sequence and functional information (via Uniprot link); and gene variants associated with *PNPLA3* (via Marrvel link) are provided in the gene information table. Supplementary Figure S2 represents the Pathway tab for the *PNPLA3* gene with links to interactions with and pathways that involve *PNPLA3* (via Pathway Commons), protein and reaction location information (via Reactome), pathway definitions (via KEGG), and pathway-related collaborative information (via Wikipathways). Supplementary Figure S3 represents the Reactions and Metabolites tabs for *PNPLA3*. It delineates 2 hydrolysis reactions where diacylglycerol (DAG) is hydrolyzed to 2-monoacylglycerol (2-MAG), and triacylglycerol (TAG) is hydrolyzed to DAG by *PNPLA3*. Three generic compounds (TAG, DAG, and fatty acid) participate in the reactions. The Metabolites tab for *PNPLA3* lists all the metabolites (with substitutions for TAG and DAG) along with corresponding RefMet names or KEGG metabolite names (in the absence of RefMet names) along with a MetStat link that points to metabolite statistics information such as a histogram of the RSD metabolite data, and analysis of variance results for the metabolite with a cutoff p-value in the MW via MetStat, with the anatomy and disease filters applied. Supplementary Figure S4 depicts the Studies tab in MetGENE for the gene *PNPLA3*. Each metabolite displays the corresponding Study IDs if measured/listed in any studies deposited in the MW, hyperlinked to the study description. A hover text displays the study title. The tool provides the user with the ability to combine studies for metabolites of interest to a consolidated view, as shown in Supplementary Figure S4. Supplementary Figure S5 serves as an informative visual aid, providing an overview of the information flow network within MetGENE for the *PNPLA3* use case. It simplifies the complexity of the underlying technical details of MetGENE, facilitating comprehension of the query-to-results process. All MetGENE tables can be downloaded in formats such as JSON and CSV directly from the respective pages in the browser or via the REST API. The REST API supports JSON and text formats. They are deposited in the Smart API repository along with the accompanying documentation.

## Discussion

Given a gene(s), the MetGENE tool identifies associations between the gene(s) and the metabolites that are biosynthesized or catabolized by proteins coded by those gene(s). It is a knowledge-based data aggregator, accessing and integrating data from resources such as the KEGG and MW. The gene(s) link to metabolites, the chemical transformations involving the metabolites through gene-specified proteins/enzymes, the functional association of these gene-associated metabolites, and the pathways involving these metabolites with context-based filtering based on anatomy (sample source) and disease/phenotype. The user can specify the gene using a multiplicity of IDs, and the gene ID conversion tool translates these into harmonized IDs that are the basis for metabolite associations. Further, all studies involving the metabolites associated with the gene-coded proteins, as present in the MW, will be accessible to the user as a stand-alone tool or via the portal interface for the NIH Common Fund National Metabolomics Data Repository. The user can begin their journey either from the main webpage for MetGENE (see Availability) or from the NIH CFDE portal (https://app.nih-cfde.org/; the steps are: Data Browser → Vocabulary → Gene).

## Potential Implications

Features from MetGENE will contribute to the integration of other omics data to draw metabolomics perspectives and with metabolomics data, with genes serving as the bridging nodes. For example, tools such as MetGENE will assist a researcher in interpreting the results of multiomics data integration holistically, where they can consider both gene-related and metabolomics data in a metabolic pathway. We are also in the process of integrating MetGENE with tools from other DCCs as a part of a broader NIH-CFDE initiative. Through that, we can provide support for persistent and shareable MetGENE-based workflows.

## Supplementary Material

giad089_Supplemental_File

giad089_GIGA-D-23-00021_Original_Submission

giad089_GIGA-D-23-00021_Revision_1

giad089_GIGA-D-23-00021_Revision_2

giad089_GIGA-D-23-00021_Revision_3

giad089_Response_to_Reviewer_Comments_Original_Submission

giad089_Response_to_Reviewer_Comments_Revision_1

giad089_Response_to_Reviewer_Comments_Revision_2

giad089_Reviewer_1_Report_Original_Submission Timothy Griffin -- 2/26/2023

giad089_Reviewer_1_Report_Revision_1 Timothy Griffin -- 6/26/2023

giad089_Reviewer_2_Report_Original_Submission Yue Wu -- 3/13/2023

giad089_Reviewer_2_Report_Revision_1 Yue Wu -- 6/25/2023

## Data Availability

The GitHub page for MetGENE [20] provides the source code, the documentation for using it, and several examples for testing the web application and the REST API. Snapshots of our code and other data further supporting this work are openly available at the *GigaScience* repository, GigaDB [22].

## Availability of Supporting Code and Requirements

MetGENE is an open-source collaborative initiative available at GitHub [20]. The main website of MetGENE and the Smart APIs are available, as shown below. MetGENE is registered with the SciCrunch registry (RRID:SCR_023402). We also plan to register MetGENE-based workflows as BioCompute Objects in the Galaxy Community Hub [21] in the near future as a part of NIH-CFDE efforts.

- Project name: MetGENE
- Project homepage: https://bdcw.org/MetGENE/index.php
- Operating system: Platform independent
- Programming languages: R and PHP
- Smart APIs: https://smart-api.info/registry/?q=MetGENE
- Source code: https://github.com/metabolomicsworkbench/MetGENE
- SciCrunch Registry: RRID:SCR_023402  https://scicrunch.org

## Additional Files

**Supplementary Fig. S1**. The gene information tab for *PNPLA3* in MetGENE has links to various online resources that provide different types of information pertaining to the gene, including sequence, gene expression, protein structure, and functional and disease information.

**Supplementary Fig. S2**. The pathway information tab for *PNPLA3* in MetGENE has links to various online resources that provide different types of information pertaining to the pathways in which PNPLA3 participates.

**Supplementary Fig. S3**. The reaction and metabolite information tabs for *PNPLA3* in MetGENE have links to reactions controlled by PNPLA3, with Reaction IDs linked to the KEGG reaction definition page. RefMet provides the metabolite details, and the MetStat link provides the context-specific metabolite measurement statistics.

**Supplementary Fig. S4**. The studies information tab for *PNPLA3* in MetGENE has links to studies in the MW pertaining to the metabolites listed. Studies can be combined for selected metabolites for further analysis.

**Supplementary Fig. S5**. A consolidated network of information flow for gene *PNPLA3* demonstrating how pathways, reactions, metabolites, studies, and summary are connected in the result.

## Abbreviations

CFDE: Common Fund Data Ecosystem; DCC: Data Coordination Centers; GTEx: Gene Tissue Expression; LINCS: Library of Integrated Network-Based Cellular Signatures; GICT: Gene ID Conversion tool; KEGG: Kyoto Encyclopedia of Genes and Genomes; MW: Metabolomics Workbench; NCBI: The National Center for Biotechnology Information.

## References

[1] Sud M, Fahy E, Cotter D et al. Metabolomics Workbench: an international repository for metabolomics data, metadata and metabolite standards, protocols, tutorials and training, and analysis tools. Nucleic Acids Res. 2016;44:D463–70. 10.1093/nar/gkv1042. 26467476 PMC4702780

[2] GTEx Consortium . Human genomics. The Genotype-Tissue Expression (GTEx) pilot analysis: multitissue gene regulation in humans. Science. 2015;348(6235):648–60. 10.1126/science.1262110. 25954001 PMC4547484

[3] Stathias V, Turner J, Koleti A, et al. LINCS Data Portal 2.0: next generation access point for perturbation-response signatures. Nucleic Acids Res. 2020;48:D431–9. 10.1093/nar/gkz1023. 31701147 PMC7145650

[4] Kanehisa M, Goto S. KEGG: Kyoto Encyclopedia of Genes and Genomes. Nucleic Acids Res. 2000;28(1):27–30. 10.1093/nar/28.1.27. 10592173 PMC102409

[5] Safran M, Rosen N, Twik M, et al. The GeneCards Suite. In: Abugessaisa I, Kasukawa T, eds. Practical Guide to Life Science Databases. Singapore: Springer, 2021, 27–56. 10.1007/978-981-16-5812-9_2.

[6] Caspi R, Billington R, Keseler I, et al. The MetaCyc database of metabolic pathways and enzymes—a 2019 update. Nucleic Acids Res. 2020;48:D445–53. 10.1093/nar/gkz862. 31586394 PMC6943030

[7] Schloerke B, Allen J. plumber: an API Generator for R. *GitHub*. 2022. https://github.com/rstudio/plumber.

[8] NCBI Resource Coordinators . Database resources of the National Center for Biotechnology Information. Nucleic Acids Res. 2000;44(D1):D7–D19. 10.1093/nar/gkv1290. PMC4702911 26615191

[9] Federhen S . The NCBI Taxonomy database. Nucleic Acids Res. 2012;40:D136–43. 10.1093/nar/gkr1178. 22139910 PMC3245000

[10] Cunningham F, Allen JE, Allen J, et al. Ensembl 2022. Nucleic Acids Res. 2022;50(D1):D988–95. 10.1093/nar/gkab1049. 34791404 PMC8728283

[11] The Uniprot Consortium . UniProt: the universal protein knowledgebase in 2021. Nucleic Acids Res. 2021;49(D1):D480–89. 10.1093/nar/gkaa1100. 33237286 PMC7778908

[12] Wang J, Al-Ouran R, Hu Y et al. MARRVEL: Integration of Human and Model Organism Genetic Resources to Facilitate Functional Annotation of the Human Genome. Am J Hum Genet. 2017;100(6):843–53. 10.1016/j.ajhg.2017.04.010. 28502612 PMC5670038

[13] Cerami EG, Gross BE, Demir E et al. Pathway Commons, a web resource for biological pathway data. Nucleic Acids Res. 2011;39:D685–90. 10.1093/nar/gkq1039. 21071392 PMC3013659

[14] Gillespie M, Jassal B, Stephan R, et al. The reactome pathway knowledgebase 2022. Nucleic Acids Res. 2021;50(D1):D687–92. 10.1093/nar/gkab1028. PMC8689983 34788843

[15] Martens M, Ammar A, Riutta A et al. WikiPathways: connecting communities. Nucleic Acids Res. 2021;49:D613–21. 10.1093/nar/gkaa1024. 33211851 PMC7779061

[16] Cohen J, Horton J, Hobbs H. Human fatty liver disease: old questions and new insights. Science. 2011;332(6037):1519–23. 10.1126/science.1204265. 21700865 PMC3229276

[17] Gorden D, Myers D, Ivanova P, et al. Biomarkers of NAFLD progression: a lipidomics approach to an epidemic. J Lipid Res. 2015;56(3):722–36. 10.1194/jlr.P056002. 25598080 PMC4340319

[18] Pingitore P, Romeo S. The role of PNPLA3 in health and disease. Biochem Biophys Acta Mol Cell Biol Lipids. 2019;1864(6):900–6. 10.1016/j.bbalip.2018.06.018. 29935383

[19] Dong X . PNPLA3—a potential therapeutic target for personalized treatment of chronic liver disease. Front Med. 2019;6. 304. 10.3389/fmed.2019.00304. PMC6927947 31921875

[20] Srinivasan S, Maurya MR, Ramachandran S, et al. MetGENE: gene-centric metabolomics information retrieval tool. *GitHub*. 2023. https://github.com/metabolomicsworkbench/MetGENE. 10.1093/gigascience/giad089 PMC10659118 37983749

[21] Mazumder R, Keeney J, King H, et al. Development of BioCompute objects for integration into galaxy in a cloud computing environment. In: Exploring clouds for acceleration of science (E-CAS) panel at the 2020 Internet2 Global Summit. 2020. https://www.youtube.com/watch?v=8pwss1SY8Tg (Accessed 12 October 2023).

[22] Srinivasan S, Maurya MR, Ramachandran S, et al. Supporting data for “MetGENE: Gene-Centric Metabolomics Information Retrieval Tool.”. GigaScience Database. 2023. 10.5524/102452. PMC10659118 37983749

