# Supplementary figures and images for: MetGENE: gene-centric metabolomics information retrieval tool
Source: Gigascience. 2023 Nov 20;12:giad089. doi: 10.1093/gigascience/giad089 (PMC10659118; doi:10.1093/gigascience/giad089)

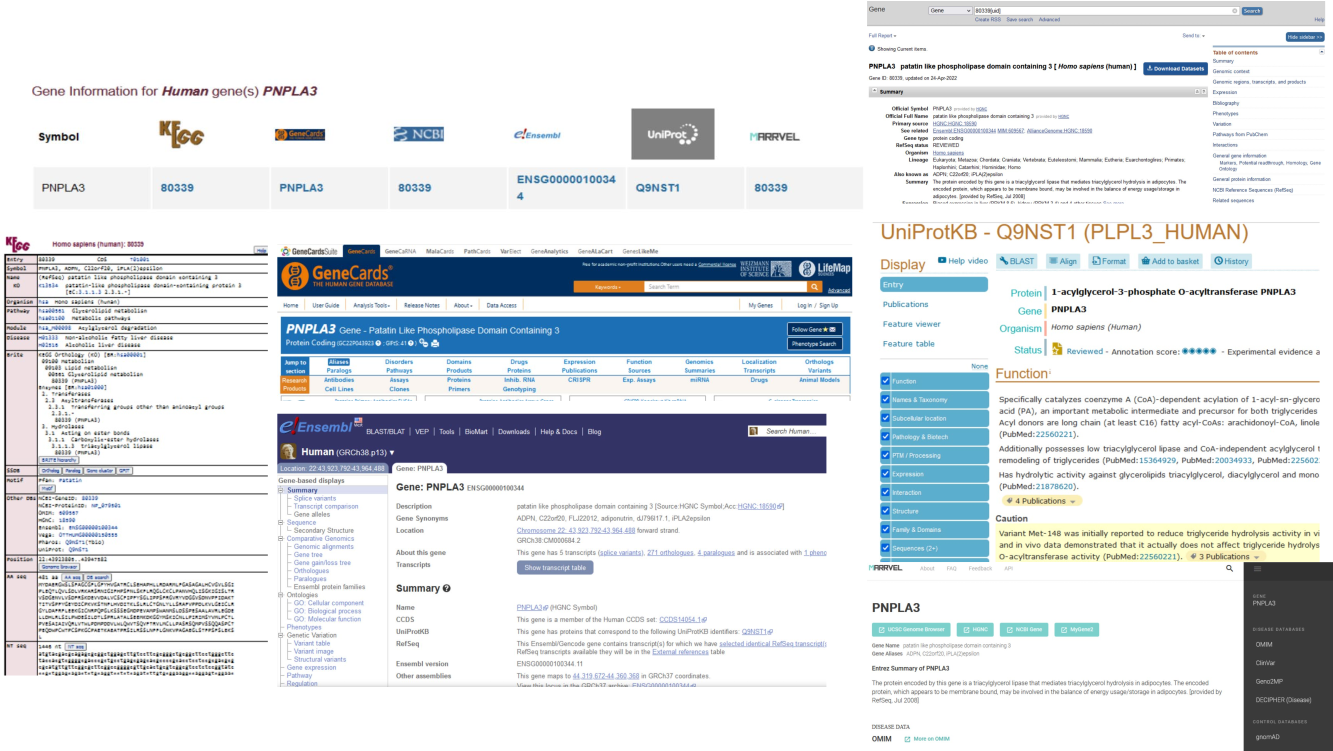

Supplement: giad089_Supplemental_File [file giad089_supplemental_file.zip › FigureA1_Supplementary.png]

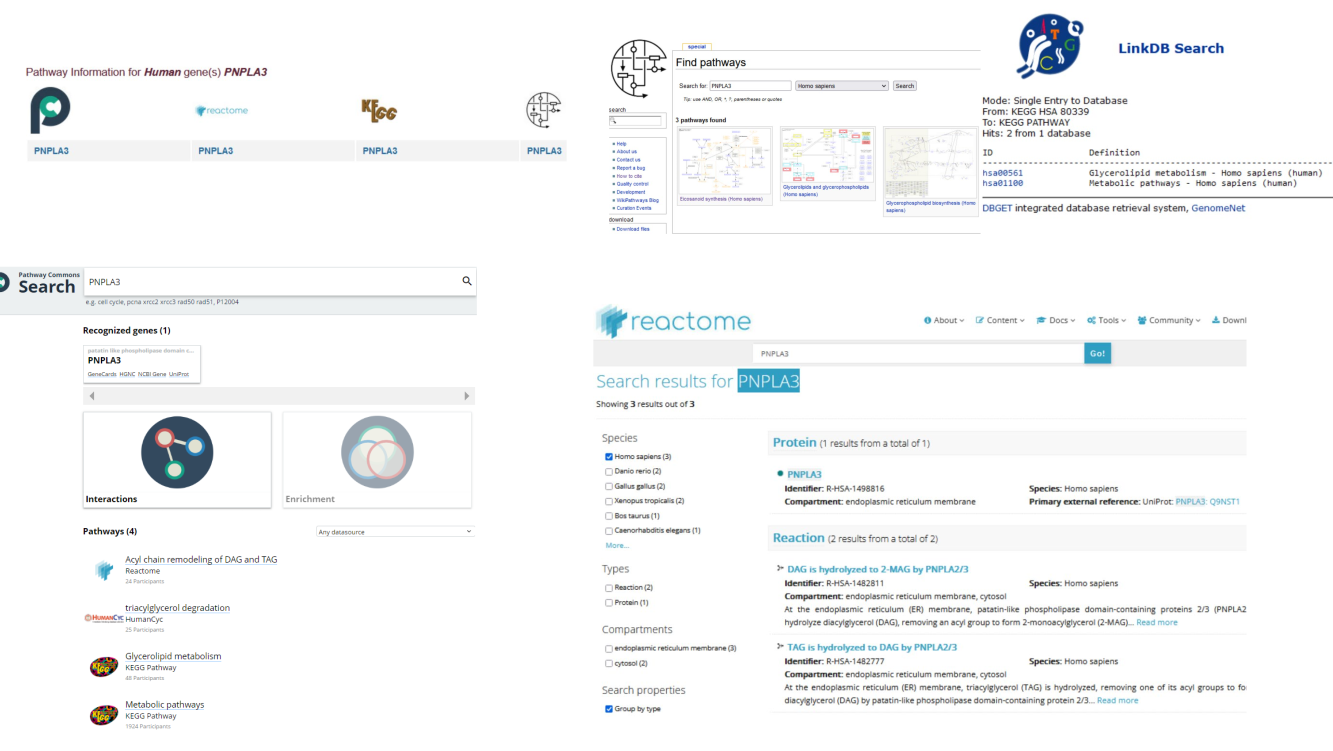

Supplement: giad089_Supplemental_File [file giad089_supplemental_file.zip › FigureA2_Supplementary.png]

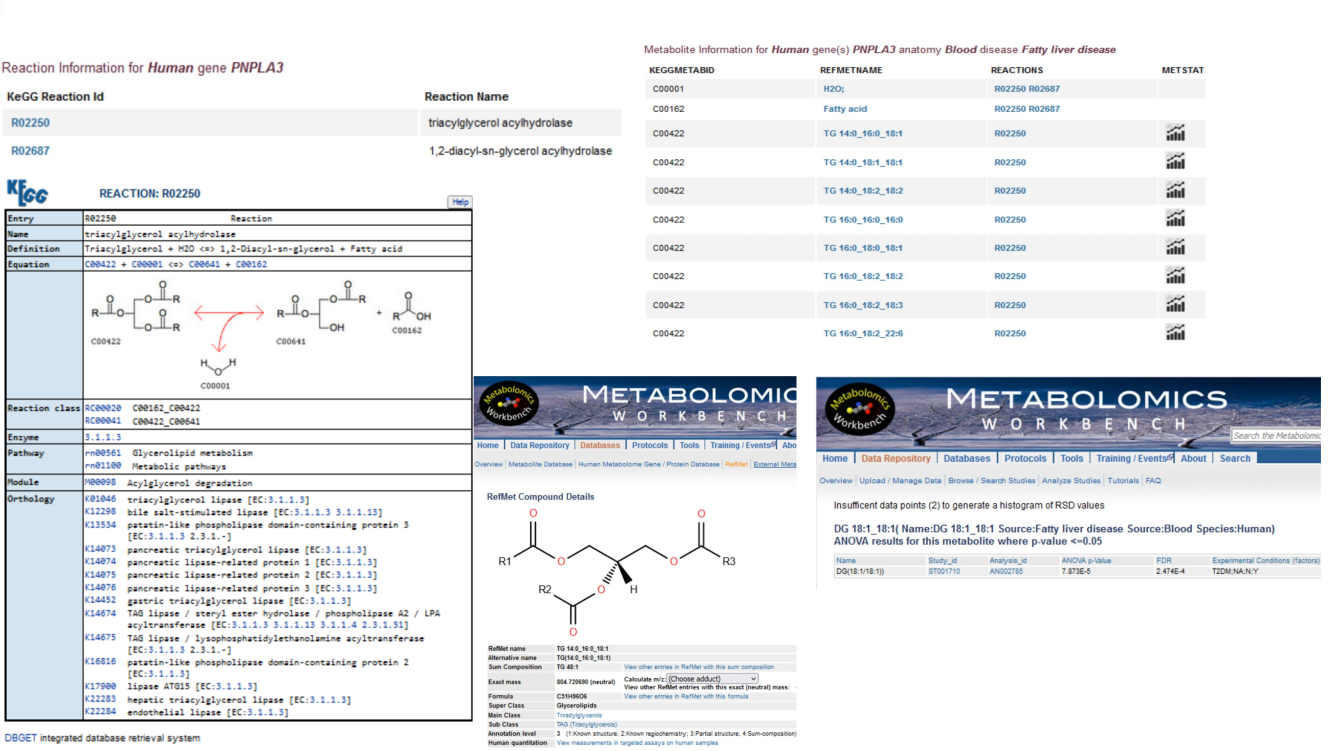

Supplement: giad089_Supplemental_File [file giad089_supplemental_file.zip › FigureA3_Supplementary.png]

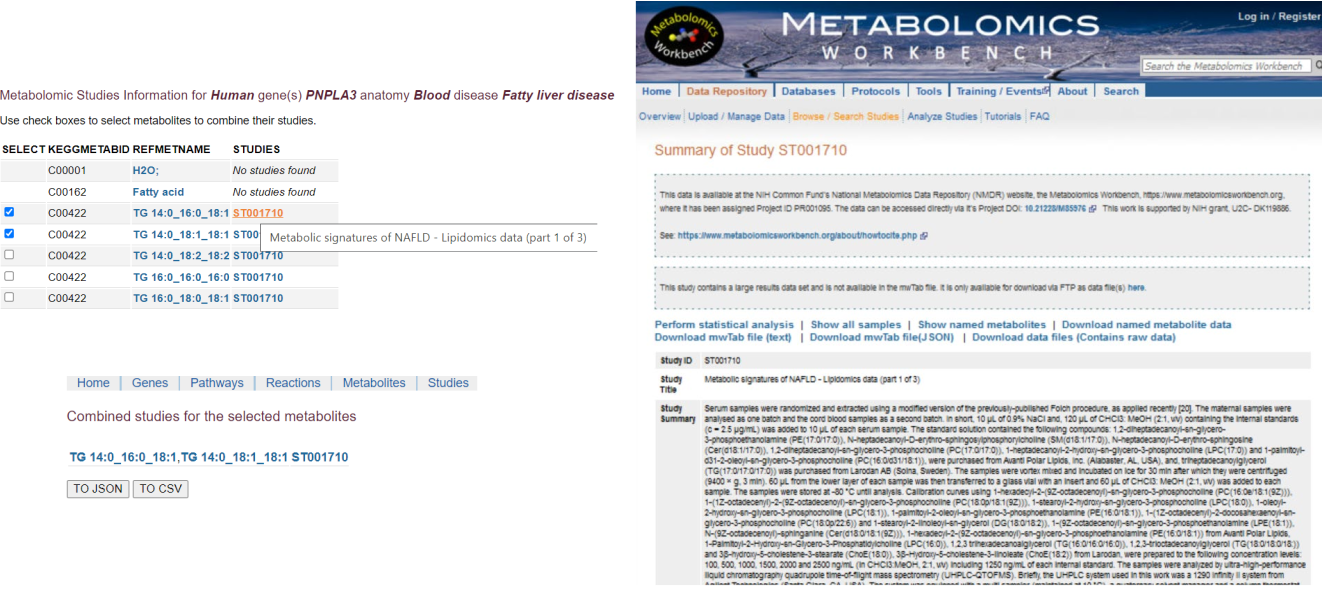

Supplement: giad089_Supplemental_File [file giad089_supplemental_file.zip › FigureA4_Supplementary.png]

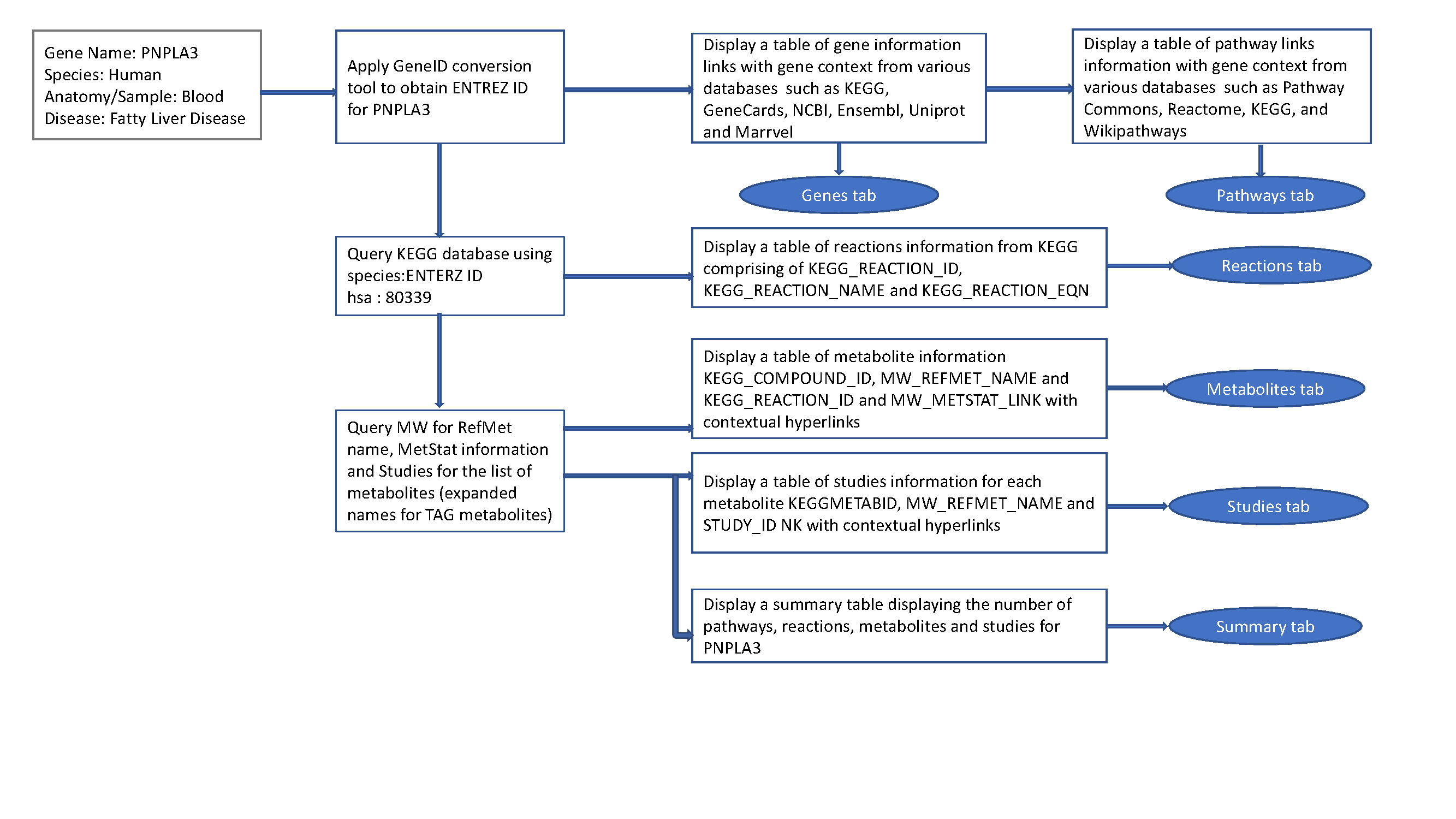

Supplement: giad089_Supplemental_File [file giad089_supplemental_file.zip › FigureA5_Supplementary.png]
