# Supplementary material for: MetGENE: gene-centric metabolomics information retrieval tool
Source: Gigascience. 2023 Nov 20;12:giad089. doi: 10.1093/gigascience/giad089 (PMC10659118; doi:10.1093/gigascience/giad089)
Supplement: giad089_GIGA-D-23-00021_Original_Submission [file giad089_giga-d-23-00021_original_submission.pdf]

GigaScience

MetGENE: Gene-centric Metabolomics Information Retrieval Tool

--Manuscript Draft--

**Manuscript Number:**	GIGA-D-23-00021
**Full Title:**	MetGENE: Gene-centric Metabolomics Information Retrieval Tool
**Article Type:**	Research
**Funding Information:**	National Institutes of Health (U2C-DK119886) — Dr Shankar Subramaniam<br>National Institutes of Health (OT2-OD030544) — Dr Shankar Subramaniam
**Abstract:**	Background Biomedical research often involves contextual integration of multi-modal and multi-omic data in search of mechanisms for improved diagnosis, treatment and monitoring. Researchers need to access information from diverse sources comprising data in various and sometimes incomplete formats. The downstream processing of the data, to decipher mechanisms by reconstructing networks and developing quantitative models, warrants considerable effort. Results MetGENE is a knowledge-based, gene-centric data aggregator that hierarchically retrieves information about the gene(s), their related pathway(s), reaction(s), metabolite(s), and metabolomic studies from standard data repositories under one dashboard to enable ease of access through centralization of relevant information. Further, the information can be contextualized by filtering along species, anatomy (tissue) and condition (disease or phenotype).<br>Conclusions MetGENE is an open source tool that aggregates metabolite information for a given gene(s) and presents them in different computable formats, e.g., JSON, for further integration with other omics studies. MetGENE is available at https://bdcw.org/MetGENE/index.php.
**Corresponding Author:**	Shankar Subramaniam, Ph.D.<br>University of California San Diego<br>La Jolla, CA UNITED STATES
**Corresponding Author Secondary Information:**	
**Corresponding Author's Institution:**	University of California San Diego
**Corresponding Author's Secondary Institution:**	
**First Author:**	Shankar Subramaniam, Ph.D.
**First Author Secondary Information:**	
**Order of Authors:**	Shankar Subramaniam, Ph.D.
	Sumana Srinivasan
	Mano Ram Maurya
	Srinivasan Ramachandran
	Eoin Fahy
**Order of Authors Secondary Information:**	
**Additional Information:**	
**Question**	**Response**
Are you submitting this manuscript to a special series or article collection?	No
**Experimental design and statistics**	Yes

Powered by Editorial Manager® and ProduXion Manager® from Aries Systems Corporation

Full details of the experimental design and statistical methods used should be given in the Methods section, as detailed in our Minimum Standards Reporting Checklist. Information essential to interpreting the data presented should be made available in the figure legends.<br><br>Have you included all the information requested in your manuscript?	
**Resources**<br><br>A description of all resources used, including antibodies, cell lines, animals and software tools, with enough information to allow them to be uniquely identified, should be included in the Methods section. Authors are strongly encouraged to cite Research Resource Identifiers (RRIDs) for antibodies, model organisms and tools, where possible.<br><br>Have you included the information requested as detailed in our Minimum Standards Reporting Checklist?	Yes
**Availability of data and materials**<br><br>All datasets and code on which the conclusions of the paper rely must be either included in your submission or deposited in publicly available repositories (where available and ethically appropriate), referencing such data using a unique identifier in the references and in the "Availability of Data and Materials" section of your manuscript.<br><br>Have you have met the above requirement as detailed in our Minimum Standards Reporting Checklist?	Yes

Powered by Editorial Manager® and ProduXion Manager® from Aries Systems Corporation

Main manuscript Latex file
Click here to access/download;LaTeX - Main Document (TeX file);main.tex

```
This is pdfTeX, Version 3.141592653-2.6-1.40.24 (TeX Live 2022)
(preloaded format=pdflatex 2022.12.28)  20 JAN 2023 13:35
entering extended mode
 restricted \write18 enabled.
 %&-line parsing enabled.
**main.tex
(./main.tex
LaTeX2e <2022-11-01> patch level 1
L3 programming layer <2022-12-17> (./oup-contemporary.cls
Document Class: oup-contemporary 2017/06/28, v1.1
(c:/TeXLive/2022/texmf-dist/tex/latex/base/article.cls
Document Class: article 2022/07/02 v1.4n Standard LaTeX document class
(c:/TeXLive/2022/texmf-dist/tex/latex/base/size10.clo
File: size10.clo 2022/07/02 v1.4n Standard LaTeX file (size option)
)
\c@part=\count185
\c@section=\count186
\c@subsection=\count187
\c@subsubsection=\count188
\c@paragraph=\count189
\c@subparagraph=\count190
\c@figure=\count191
\c@table=\count192
\abovecaptionskip=\skip48
\belowcaptionskip=\skip49
\bibindent=\dimen140
) (c:/TeXLive/2022/texmf-dist/tex/latex/base/inputenc.sty
Package: inputenc 2021/02/14 v1.3d Input encoding file
\inpenc@prehook=\toks16
\inpenc@posthook=\toks17
) (c:/TeXLive/2022/texmf-dist/tex/latex/base/fontenc.sty
Package: fontenc 2021/04/29 v2.0v Standard LaTeX package
) (c:/TeXLive/2022/texmf-dist/tex/generic/iftex/ifpdf.sty
Package: ifpdf 2019/10/25 v3.4 ifpdf legacy package. Use iftex instead.
(c:/TeXLive/2022/texmf-dist/tex/generic/iftex/iftex.sty
Package: iftex 2022/02/03 v1.0f TeX engine tests
)) (c:/TeXLive/2022/texmf-dist/tex/latex/microtype/microtype.sty
Package: microtype 2022/06/23 v3.0f Micro-typographical refinements (RS)
(c:/TeXLive/2022/texmf-dist/tex/latex/graphics/keyval.sty
Package: keyval 2022/05/29 v1.15 key=value parser (DPC)
\KV@toks@=\toks18
) (c:/TeXLive/2022/texmf-dist/tex/latex/etoolbox/etoolbox.sty
Package: etoolbox 2020/10/05 v2.5k e-TeX tools for LaTeX (JAW)
\etb@tempcnta=\count193
)
\MT@toks=\toks19
\MT@tempbox=\box51
\MT@count=\count194
LaTeX Info: Redefining \noprotrusionifhmode on input line 1045.
LaTeX Info: Redefining \leftprotrusion on input line 1046.
LaTeX Info: Redefining \rightprotrusion on input line 1056.
LaTeX Info: Redefining \textls on input line 1234.
\MT@outer@kern=\dimen141
LaTeX Info: Redefining \textmicrotypecontext on input line 1858.
```

```
\MT@listname@count=\count195
(c:/TeXLive/2022/texmf-dist/tex/latex/microtype/microtype-pdftex.def
File: microtype-pdftex.def 2022/06/23 v3.0f Definitions specific to
pdftex (RS)

LaTeX Info: Redefining \lsstyle on input line 900.
LaTeX Info: Redefining \lslig on input line 900.
\MT@outer@space=\skip50
)
Package microtype Info: Loading configuration file microtype.cfg.
(c:/TeXLive/2022/texmf-dist/tex/latex/microtype/microtype.cfg
File: microtype.cfg 2022/06/23 v3.0f microtype main configuration file
(RS)
)) (c:/TeXLive/2022/texmf-dist/tex/latex/euler/euler.sty
Package: euler 1995/03/05 v2.5
Package: `euler' v2.5 <1995/03/05> (FJ and FMi)
LaTeX Font Info:    Redeclaring symbol font `letters' on input line 35.
LaTeX Font Info:    Encoding `OML' has changed to `U' for symbol font
(Font)              `letters' in the math version `normal' on input line
35.
LaTeX Font Info:    Overwriting symbol font `letters' in version `normal'
(Font)                  OML/cmm/m/it --> U/eur/m/n on input line 35.
LaTeX Font Info:    Encoding `OML' has changed to `U' for symbol font
(Font)              `letters' in the math version `bold' on input line
35.
LaTeX Font Info:    Overwriting symbol font `letters' in version `bold'
(Font)                  OML/cmm/b/it --> U/eur/m/n on input line 35.
LaTeX Font Info:    Overwriting symbol font `letters' in version `bold'
(Font)                  U/eur/m/n --> U/eur/b/n on input line 36.
LaTeX Font Info:    Redeclaring math symbol \Gamma on input line 47.
LaTeX Font Info:    Redeclaring math symbol \Delta on input line 48.
LaTeX Font Info:    Redeclaring math symbol \Theta on input line 49.
LaTeX Font Info:    Redeclaring math symbol \Lambda on input line 50.
LaTeX Font Info:    Redeclaring math symbol \Xi on input line 51.
LaTeX Font Info:    Redeclaring math symbol \Pi on input line 52.
LaTeX Font Info:    Redeclaring math symbol \Sigma on input line 53.
LaTeX Font Info:    Redeclaring math symbol \Upsilon on input line 54.
LaTeX Font Info:    Redeclaring math symbol \Phi on input line 55.
LaTeX Font Info:    Redeclaring math symbol \Psi on input line 56.
LaTeX Font Info:    Redeclaring math symbol \Omega on input line 57.
\symEulerFraktur=\mathgroup4
LaTeX Font Info:    Overwriting symbol font `EulerFraktur' in version
`bold'
(Font)                  U/euf/m/n --> U/euf/b/n on input line 63.
LaTeX Info: Redefining \oldstylenums on input line 85.
\symEulerScript=\mathgroup5
LaTeX Font Info:    Overwriting symbol font `EulerScript' in version
`bold'
(Font)                  U/eus/m/n --> U/eus/b/n on input line 93.
LaTeX Font Info:    Redeclaring math symbol \aleph on input line 97.
LaTeX Font Info:    Redeclaring math symbol \Re on input line 98.
LaTeX Font Info:    Redeclaring math symbol \Im on input line 99.
LaTeX Font Info:    Redeclaring math delimiter \vert on input line 101.
```

```
LaTeX Font Info:    Redeclaring math delimiter \backslash on input line
103.
LaTeX Font Info:    Redeclaring math symbol \neg on input line 106.
LaTeX Font Info:    Redeclaring math symbol \wedge on input line 108.
LaTeX Font Info:    Redeclaring math symbol \vee on input line 110.
LaTeX Font Info:    Redeclaring math symbol \setminus on input line 112.
LaTeX Font Info:    Redeclaring math symbol \sim on input line 113.
LaTeX Font Info:    Redeclaring math symbol \mid on input line 114.
LaTeX Font Info:    Redeclaring math delimiter \arrowvert on input line
116.
LaTeX Font Info:    Redeclaring math symbol \mathsection on input line
117.
\symEulerExtension=\mathgroup6
LaTeX Font Info:    Redeclaring math symbol \coprod on input line 125.
LaTeX Font Info:    Redeclaring math symbol \prod on input line 125.
LaTeX Font Info:    Redeclaring math symbol \sum on input line 125.
LaTeX Font Info:    Redeclaring math symbol \intop on input line 130.
LaTeX Font Info:    Redeclaring math symbol \ointop on input line 131.
LaTeX Font Info:    Redeclaring math symbol \braceld on input line 132.
LaTeX Font Info:    Redeclaring math symbol \bracerd on input line 133.
LaTeX Font Info:    Redeclaring math symbol \bracelu on input line 134.
LaTeX Font Info:    Redeclaring math symbol \braceru on input line 135.
LaTeX Font Info:    Redeclaring math symbol \infty on input line 136.
LaTeX Font Info:    Redeclaring math symbol \nearrow on input line 153.
LaTeX Font Info:    Redeclaring math symbol \searrow on input line 154.
LaTeX Font Info:    Redeclaring math symbol \nwarrow on input line 155.
LaTeX Font Info:    Redeclaring math symbol \swarrow on input line 156.
LaTeX Font Info:    Redeclaring math symbol \Leftrightarrow on input line
157.
LaTeX Font Info:    Redeclaring math symbol \Leftarrow on input line 158.
LaTeX Font Info:    Redeclaring math symbol \Rightarrow on input line
159.
LaTeX Font Info:    Redeclaring math symbol \leftrightarrow on input line
160.
LaTeX Font Info:    Redeclaring math symbol \leftarrow on input line 161.
LaTeX Font Info:    Redeclaring math symbol \rightarrow on input line
163.
LaTeX Font Info:    Redeclaring math delimiter \uparrow on input line
166.
LaTeX Font Info:    Redeclaring math delimiter \downarrow on input line
168.
LaTeX Font Info:    Redeclaring math delimiter \updownarrow on input line
170.
LaTeX Font Info:    Redeclaring math delimiter \Uparrow on input line
172.
LaTeX Font Info:    Redeclaring math delimiter \Downarrow on input line
174.
LaTeX Font Info:    Redeclaring math delimiter \Updownarrow on input line
176.
LaTeX Font Info:    Redeclaring math symbol \leftharpoonup on input line
177.
LaTeX Font Info:    Redeclaring math symbol \leftharpoondown on input
line 178.
```

```
LaTeX Font Info:    Redeclaring math symbol \rightharpoonup on input line
179.
LaTeX Font Info:    Redeclaring math symbol \rightharpoondown on input
line 180
.
LaTeX Font Info:    Redeclaring math delimiter \lbrace on input line 182.
LaTeX Font Info:    Redeclaring math delimiter \rbrace on input line 184.
\symcmmigroup=\mathgroup7
LaTeX Font Info:    Overwriting symbol font `cmmigroup' in version `bold'
(Font)                  OML/cmm/m/it --> OML/cmm/b/it on input line 200.
LaTeX Font Info:    Redeclaring math accent \vec on input line 201.
LaTeX Font Info:    Redeclaring math symbol \triangleleft on input line
202.
LaTeX Font Info:    Redeclaring math symbol \triangleright on input line
203.
LaTeX Font Info:    Redeclaring math symbol \star on input line 204.
LaTeX Font Info:    Redeclaring math symbol \lhook on input line 205.
LaTeX Font Info:    Redeclaring math symbol \rhook on input line 206.
LaTeX Font Info:    Redeclaring math symbol \flat on input line 207.
LaTeX Font Info:    Redeclaring math symbol \natural on input line 208.
LaTeX Font Info:    Redeclaring math symbol \sharp on input line 209.
LaTeX Font Info:    Redeclaring math symbol \smile on input line 210.
LaTeX Font Info:    Redeclaring math symbol \frown on input line 211.
LaTeX Font Info:    Redeclaring math accent \grave on input line 245.
LaTeX Font Info:    Redeclaring math accent \acute on input line 246.
LaTeX Font Info:    Redeclaring math accent \tilde on input line 247.
LaTeX Font Info:    Redeclaring math accent \ddot on input line 248.
LaTeX Font Info:    Redeclaring math accent \check on input line 249.
LaTeX Font Info:    Redeclaring math accent \breve on input line 250.
LaTeX Font Info:    Redeclaring math accent \bar on input line 251.
LaTeX Font Info:    Redeclaring math accent \dot on input line 252.
LaTeX Font Info:    Redeclaring math accent \hat on input line 254.
) (c:/TeXLive/2022/texmf-dist/tex/latex/merriweather/merriweather.sty
Package: merriweather 2022/09/20 (Bob Tennent) Supports
Merriweather(Sans) font
s for all LaTeX engines.
(c:/TeXLive/2022/texmf-dist/tex/generic/iftex/ifxetex.sty
Package: ifxetex 2019/10/25 v0.7 ifxetex legacy package. Use iftex
instead.
) (c:/TeXLive/2022/texmf-dist/tex/generic/iftex/ifluatex.sty
Package: ifluatex 2019/10/25 v1.5 ifluatex legacy package. Use iftex
instead.
) (c:/TeXLive/2022/texmf-dist/tex/latex/base/textcomp.sty
Package: textcomp 2020/02/02 v2.0n Standard LaTeX package
) (c:/TeXLive/2022/texmf-dist/tex/latex/xkeyval/xkeyval.sty
Package: xkeyval 2022/06/16 v2.9 package option processing (HA)
(c:/TeXLive/2022/texmf-dist/tex/generic/xkeyval/xkeyval.tex
(c:/TeXLive/2022/te
xmf-dist/tex/generic/xkeyval/xkvutils.tex
\XKV@toks=\toks20
\XKV@tempa@toks=\toks21
)
\XKV@depth=\count196
File: xkeyval.tex 2014/12/03 v2.7a key=value parser (HA)
```

```
)) (c:/TeXLive/2022/texmf-dist/tex/latex/base/fontenc.sty
Package: fontenc 2021/04/29 v2.0v Standard LaTeX package
) (c:/TeXLive/2022/texmf-dist/tex/latex/fontaxes/fontaxes.sty
Package: fontaxes 2020/07/21 v1.0e Font selection axes
LaTeX Info: Redefining \upshape on input line 29.
LaTeX Info: Redefining \itshape on input line 31.
LaTeX Info: Redefining \slshape on input line 33.
LaTeX Info: Redefining \swshape on input line 35.
LaTeX Info: Redefining \scshape on input line 37.
LaTeX Info: Redefining \sscshape on input line 39.
LaTeX Info: Redefining \ulcshape on input line 41.
LaTeX Info: Redefining \textsw on input line 47.
LaTeX Info: Redefining \textssc on input line 48.
LaTeX Info: Redefining \textulc on input line 49.
)) (c:/TeXLive/2022/texmf-dist/tex/latex/mathastext/mathastext.sty
Package: mathastext 2022/11/04 v1.3y Use the text font in math mode (JFB)
\mst@exists@muskip=\muskip16
\mst@forall@muskip=\muskip17
\mst@prime@muskip=\muskip18
\mst@do@nonletters=\toks22
\mst@do@easynonletters=\toks23
\mst@do@az=\toks24
\mst@do@AZ=\toks25
\symmtoperatorfont=\mathgroup8
\symmtletterfont=\mathgroup9
** ! and ?
** punctuation: , . : ; and \colon
LaTeX Info: Redefining \relbar on input line 844.
LaTeX Info: Redefining \rightarrowfill on input line 847.
LaTeX Info: Redefining \leftarrowfill on input line 852.
** + and =
LaTeX Info: Redefining \Relbar on input line 943.
** adding = ; and + to \nfss@catcodes
** parentheses ( ) [ ] and slash /
** alldelims: < > \backslash \setminus | \vert \mid \{ and \}
LaTeX Font Info:    Redeclaring math delimiter \backslash on input line
989.
LaTeX Font Info:    Redeclaring math symbol \setminus on input line 1001.
LaTeX Info: Redefining \models on input line 1010.
** \# \mathdollar \% \&
** \imath and \jmath
LaTeX Font Info:    Overwriting math alphabet `\mathnormalbold' in
version `nor
mal'
(Font)                  T1/Merriwthr-OsF/b/it --> T1/Merriwthr-OsF/b/it
on inpu
t line 2370.
LaTeX Font Info:    Overwriting math alphabet `\mathnormalbold' in
version `bol
d'
(Font)                  T1/Merriwthr-OsF/b/it --> T1/Merriwthr-OsF/b/it
on inpu
t line 2370.
```

```
LaTeX Font Info:    Overwriting symbol font `mtletterfont' in version
`normal'
(Font)                  T1/Merriwthr-OsF/m/it --> T1/Merriwthr-OsF/m/it
on inpu
t line 2370.
LaTeX Font Info:    Overwriting symbol font `mtletterfont' in version
`bold'
(Font)                  T1/Merriwthr-OsF/m/it --> T1/Merriwthr-OsF/b/it
on inpu
t line 2370.
LaTeX Font Info:    Overwriting symbol font `mtoperatorfont' in version
`normal
'
(Font)                  T1/Merriwthr-OsF/m/n --> T1/Merriwthr-OsF/m/n on
input
line 2370.
LaTeX Font Info:    Overwriting symbol font `mtoperatorfont' in version
`bold'
(Font)                  T1/Merriwthr-OsF/m/n --> T1/Merriwthr-OsF/b/n on
input
line 2370.
LaTeX Font Info:    Overwriting math alphabet `\Mathbf' in version
`normal'
(Font)                  T1/Merriwthr-OsF/b/n --> T1/Merriwthr-OsF/b/n on
input
line 2370.
LaTeX Font Info:    Overwriting math alphabet `\Mathbf' in version `bold'
(Font)                  T1/Merriwthr-OsF/b/n --> T1/Merriwthr-OsF/b/n on
input
line 2370.
LaTeX Font Info:    Overwriting math alphabet `\Mathit' in version
`normal'
(Font)                  T1/Merriwthr-OsF/m/it --> T1/Merriwthr-OsF/m/it
on inpu
t line 2370.
LaTeX Font Info:    Overwriting math alphabet `\Mathit' in version `bold'
(Font)                  T1/Merriwthr-OsF/m/it --> T1/Merriwthr-OsF/b/it
on inpu
t line 2370.
LaTeX Font Info:    Overwriting math alphabet `\Mathsf' in version
`normal'
(Font)                  T1/MerriwthrSans-OsF/m/n --> T1/MerriwthrSans-
OsF/m/n o
n input line 2370.
LaTeX Font Info:    Overwriting math alphabet `\Mathsf' in version `bold'
(Font)                  T1/MerriwthrSans-OsF/m/n --> T1/MerriwthrSans-
OsF/b/n o
n input line 2370.
LaTeX Font Info:    Overwriting math alphabet `\Mathtt' in version
`normal'
(Font)                  T1/lmtt/m/n --> T1/lmtt/m/n on input line 2370.
LaTeX Font Info:    Overwriting math alphabet `\Mathtt' in version `bold'
(Font)                  T1/lmtt/m/n --> T1/lmtt/b/n on input line 2370.
** Latin letters in the `normal' (resp. `bold') math versions are now
```

```
** set up to use the fonts T1/Merriwthr-OsF/m(b)/it
** Other characters (digits, ...) and \log-like names will be
** typeset with the n shape.
** \hbar
** minus as endash
** \HUGE has been (re)-defined.
** mathastext has declared larger sizes for subscripts.
** To keep LaTeX defaults, use option `defaultmathsizes'.
) (c:/TeXLive/2022/texmf-dist/tex/latex/relsize/relsize.sty
Package: relsize 2013/03/29 ver 4.1
) (c:/TeXLive/2022/texmf-dist/tex/latex/ragged2e/ragged2e.sty
Package: ragged2e 2022/11/13 v3.2 ragged2e Package
\CenteringLeftskip=\skip51
\RaggedLeftLeftskip=\skip52
\RaggedRightLeftskip=\skip53
\CenteringRightskip=\skip54
\RaggedLeftRightskip=\skip55
\RaggedRightRightskip=\skip56
\CenteringParfillskip=\skip57
\RaggedLeftParfillskip=\skip58
\RaggedRightParfillskip=\skip59
\JustifyingParfillskip=\skip60
\CenteringParindent=\skip61
\RaggedLeftParindent=\skip62
\RaggedRightParindent=\skip63
\JustifyingParindent=\skip64
) (c:/TeXLive/2022/texmf-dist/tex/latex/xcolor/xcolor.sty
Package: xcolor 2022/06/12 v2.14 LaTeX color extensions (UK)
(c:/TeXLive/2022/texmf-dist/tex/latex/graphics-cfg/color.cfg
File: color.cfg 2016/01/02 v1.6 sample color configuration
)
Package xcolor Info: Driver file: pdftex.def on input line 227.
(c:/TeXLive/2022/texmf-dist/tex/latex/graphics-def/pdftex.def
File: pdftex.def 2022/09/22 v1.2b Graphics/color driver for pdftex
) (c:/TeXLive/2022/texmf-dist/tex/latex/graphics/mathcolor.ltx)
Package xcolor Info: Model `cmy' substituted by `cmy0' on input line
1353.
Package xcolor Info: Model `hsb' substituted by `rgb' on input line 1357.
Package xcolor Info: Model `RGB' extended on input line 1369.
Package xcolor Info: Model `HTML' substituted by `rgb' on input line
1371.
Package xcolor Info: Model `Hsb' substituted by `hsb' on input line 1372.
Package xcolor Info: Model `tHsb' substituted by `hsb' on input line
1373.
Package xcolor Info: Model `HSB' substituted by `hsb' on input line 1374.
Package xcolor Info: Model `Gray' substituted by `gray' on input line
1375.
Package xcolor Info: Model `wave' substituted by `hsb' on input line
1376.
) (c:/TeXLive/2022/texmf-dist/tex/latex/colortbl/colortbl.sty
Package: colortbl 2022/06/20 v1.0f Color table columns (DPC)
(c:/TeXLive/2022/texmf-dist/tex/latex/tools/array.sty
Package: array 2022/09/04 v2.5g Tabular extension package (FMi)
\col@sep=\dimen142
```

```
\ar@mcellbox=\box52
\extrarowheight=\dimen143
\NC@list=\toks26
\extratabsurround=\skip65
\backup@length=\skip66
\ar@cellbox=\box53
)
\everycr=\toks27
\minrowclearance=\skip67
\rownum=\count197
) (c:/TeXLive/2022/texmf-dist/tex/latex/graphics/graphicx.sty
Package: graphicx 2021/09/16 v1.2d Enhanced LaTeX Graphics (DPC,SPQR)
(c:/TeXLive/2022/texmf-dist/tex/latex/graphics/graphics.sty
Package: graphics 2022/03/10 v1.4e Standard LaTeX Graphics (DPC,SPQR)
(c:/TeXLive/2022/texmf-dist/tex/latex/graphics/trig.sty
Package: trig 2021/08/11 v1.11 sin cos tan (DPC)
) (c:/TeXLive/2022/texmf-dist/tex/latex/graphics-cfg/graphics.cfg
File: graphics.cfg 2016/06/04 v1.11 sample graphics configuration
)
Package graphics Info: Driver file: pdftex.def on input line 107.
)
\Gin@req@height=\dimen144
\Gin@req@width=\dimen145
) (c:/TeXLive/2022/texmf-dist/tex/latex/xpatch/xpatch.sty
(c:/TeXLive/2022/texm
f-dist/tex/latex/l3kernel/expl3.sty
Package: expl3 2023-01-16 L3 programming layer (loader)

! LaTeX Error: Mismatched LaTeX support files detected.
(LaTeX)        Loading 'expl3.sty' aborted!
(LaTeX)
(LaTeX)        The L3 programming layer in the LaTeX format
(LaTeX)        is dated 2022-12-17, but in your TeX tree the files
require
(LaTeX)        at least 2023-01-16.

For immediate help type H <return>.
 ...

l.77      \ExplLoaderFileDate{expl3.sty}}
                                          %

The most likely causes are:
 - A recent format generation failed;
 - A stray format file in the user tree which needs to be removed or
rebuilt;
 - You are running a manually installed version of expl3.sty
   which is incompatible with the version in LaTeX.

LaTeX will abort loading the incompatible support files but this may lead
to
later errors. Please ensure that your LaTeX format is correctly
regenerated.
```

```
)
Package: xpatch 2020/03/25 v0.3a Extending etoolbox patching commands
(c:/TeXLive/2022/texmf-dist/tex/latex/l3packages/xparse/xparse.sty
Package: xparse 2022-12-17 L3 Experimental document command parser
)) (c:/TeXLive/2022/texmf-dist/tex/latex/environ/environ.sty
Package: environ 2014/05/04 v0.3 A new way to define environments
(c:/TeXLive/2022/texmf-dist/tex/latex/trimspaces/trimspaces.sty
Package: trimspaces 2009/09/17 v1.1 Trim spaces around a token list
)
\@envbody=\toks28
) (c:/TeXLive/2022/texmf-dist/tex/latex/lastpage/lastpage.sty
Package: lastpage 2021/09/03 v1.2n Refers to last page's name (HMM; JPG)
) (c:/TeXLive/2022/texmf-dist/tex/latex/graphics/rotating.sty
Package: rotating 2016/08/11 v2.16d rotated objects in LaTeX
(c:/TeXLive/2022/texmf-dist/tex/latex/base/ifthen.sty
Package: ifthen 2022/04/13 v1.1d Standard LaTeX ifthen package (DPC)
)
\c@r@tfl@t=\count198
\rotFPtop=\skip68
\rotFPbot=\skip69
\rot@float@box=\box54
\rot@mess@toks=\toks29
) (c:/TeXLive/2022/texmf-dist/tex/latex/graphics/lscape.sty
Package: lscape 2020/05/28 v3.02 Landscape Pages (DPC)
) (c:/TeXLive/2022/texmf-dist/tex/latex/tools/afterpage.sty
Package: afterpage 2014/10/28 v1.08 After-Page Package (DPC)
\AP@output=\toks30
\AP@partial=\box55
\AP@footins=\box56
) (c:/TeXLive/2022/texmf-dist/tex/latex/textpos/textpos.sty
Package: textpos 2022/07/23 v1.10.1
Package textpos Info: choosing support for LaTeX3 on input line 60.
\TP@textbox=\box57
\TP@holdbox=\box58
\TPHorizModule=\dimen146
\TPVertModule=\dimen147
\TP@margin=\dimen148
\TP@absmargin=\dimen149
Grid set 16 x 16 = 37.34424pt x 52.81541pt
\TPboxrulesize=\dimen150
\TP@ox=\dimen151
\TP@oy=\dimen152
\TP@tbargs=\toks31
TextBlockOrigin set to 0pt x 0pt
) (c:/TeXLive/2022/texmf-dist/tex/latex/url/url.sty
\Urlmuskip=\muskip19
Package: url 2013/09/16  ver 3.4  Verb mode for urls, etc.
) (c:/TeXLive/2022/texmf-dist/tex/latex/newfloat/newfloat.sty
Package: newfloat 2019/09/02 v1.1l Defining new floating environments
(AR)
Package newfloat Info: `rotating' package detected.
) (c:/TeXLive/2022/texmf-dist/tex/latex/mdframed/mdframed.sty
Package: mdframed 2013/07/01 1.9b: mdframed
```

```
(c:/TeXLive/2022/texmf-dist/tex/latex/kvoptions/kvoptions.sty
Package: kvoptions 2022-06-15 v3.15 Key value format for package options
(HO)
(c:/TeXLive/2022/texmf-dist/tex/generic/ltxcmds/ltxcmds.sty
Package: ltxcmds 2020-05-10 v1.25 LaTeX kernel commands for general use
(HO)
) (c:/TeXLive/2022/texmf-dist/tex/latex/kvsetkeys/kvsetkeys.sty
Package: kvsetkeys 2022-10-05 v1.19 Key value parser (HO)
)) (c:/TeXLive/2022/texmf-dist/tex/latex/zref/zref-abspage.sty
Package: zref-abspage 2022-04-07 v2.34 Module abspage for zref (HO)
(c:/TeXLive/2022/texmf-dist/tex/latex/zref/zref-base.sty
Package: zref-base 2022-04-07 v2.34 Module base for zref (HO)
(c:/TeXLive/2022/texmf-dist/tex/generic/infwarerr/infwarerr.sty
Package: infwarerr 2019/12/03 v1.5 Providing info/warning/error messages
(HO)
) (c:/TeXLive/2022/texmf-dist/tex/generic/kvdefinekeys/kvdefinekeys.sty
Package: kvdefinekeys 2019-12-19 v1.6 Define keys (HO)
) (c:/TeXLive/2022/texmf-dist/tex/generic/pdftexcmds/pdftexcmds.sty
Package: pdftexcmds 2020-06-27 v0.33 Utility functions of pdfTeX for
LuaTeX (HO
)
Package pdftexcmds Info: \pdf@primitive is available.
Package pdftexcmds Info: \pdf@ifprimitive is available.
Package pdftexcmds Info: \pdfdraftmode found.
) (c:/TeXLive/2022/texmf-dist/tex/generic/etexcmds/etexcmds.sty
Package: etexcmds 2019/12/15 v1.7 Avoid name clashes with e-TeX commands
(HO)
) (c:/TeXLive/2022/texmf-dist/tex/latex/auxhook/auxhook.sty
Package: auxhook 2019-12-17 v1.6 Hooks for auxiliary files (HO)
)
Package zref Info: New property list: main on input line 767.
Package zref Info: New property: default on input line 768.
Package zref Info: New property: page on input line 769.
) (c:/TeXLive/2022/texmf-dist/tex/latex/base/atbegshi-ltx.sty
Package: atbegshi-ltx 2021/01/10 v1.0c Emulation of the original atbegshi
package with kernel methods
)
\c@abspage=\count199
Package zref Info: New property: abspage on input line 65.
) (c:/TeXLive/2022/texmf-dist/tex/latex/needspace/needspace.sty
Package: needspace 2010/09/12 v1.3d reserve vertical space
)
\mdf@templength=\skip70
\c@mdf@globalstyle@cnt=\count266
\mdf@skipabove@length=\skip71
\mdf@skipbelow@length=\skip72
\mdf@leftmargin@length=\skip73
\mdf@rightmargin@length=\skip74
\mdf@innerleftmargin@length=\skip75
\mdf@innerrightmargin@length=\skip76
\mdf@innertopmargin@length=\skip77
\mdf@innerbottommargin@length=\skip78
\mdf@splittopskip@length=\skip79
\mdf@splitbottomskip@length=\skip80
```

```
\mdf@outermargin@length=\skip81
\mdf@innermargin@length=\skip82
\mdf@linewidth@length=\skip83
\mdf@innerlinewidth@length=\skip84
\mdf@middlelinewidth@length=\skip85
\mdf@outerlinewidth@length=\skip86
\mdf@roundcorner@length=\skip87
\mdf@footenotedistance@length=\skip88
\mdf@userdefinedwidth@length=\skip89
\mdf@needspace@length=\skip90
\mdf@frametitleaboveskip@length=\skip91
\mdf@frametitlebelowskip@length=\skip92
\mdf@frametitlerulewidth@length=\skip93
\mdf@frametitleleftmargin@length=\skip94
\mdf@frametitlerightmargin@length=\skip95
\mdf@shadowsize@length=\skip96
\mdf@extratopheight@length=\skip97
\mdf@subtitleabovelinewidth@length=\skip98
\mdf@subtitlebelowlinewidth@length=\skip99
\mdf@subtitleaboveskip@length=\skip100
\mdf@subtitlebelowskip@length=\skip101
\mdf@subtitleinneraboveskip@length=\skip102
\mdf@subtitleinnerbelowskip@length=\skip103
\mdf@subsubtitleabovelinewidth@length=\skip104
\mdf@subsubtitlebelowlinewidth@length=\skip105
\mdf@subsubtitleaboveskip@length=\skip106
\mdf@subsubtitlebelowskip@length=\skip107
\mdf@subsubtitleinneraboveskip@length=\skip108
\mdf@subsubtitleinnerbelowskip@length=\skip109
(c:/TeXLive/2022/texmf-dist/tex/latex/mdframed/md-frame-0.mdf
File: md-frame-0.mdf 2013/07/01\ 1.9b: md-frame-0
)
\mdf@frametitlebox=\box59
\mdf@footnotebox=\box60
\mdf@splitbox@one=\box61
\mdf@splitbox@two=\box62
\mdf@splitbox@save=\box63
\mdfsplitboxwidth=\skip110
\mdfsplitboxtotalwidth=\skip111
\mdfsplitboxheight=\skip112
\mdfsplitboxdepth=\skip113
\mdfsplitboxtotalheight=\skip114
\mdfframetitleboxwidth=\skip115
\mdfframetitleboxtotalwidth=\skip116
\mdfframetitleboxheight=\skip117
\mdfframetitleboxdepth=\skip118
\mdfframetitleboxtotalheight=\skip119
\mdffootnoteboxwidth=\skip120
\mdffootnoteboxtotalwidth=\skip121
\mdffootnoteboxheight=\skip122
\mdffootnoteboxdepth=\skip123
\mdffootnoteboxtotalheight=\skip124
\mdftotallinewidth=\skip125
\mdfboundingboxwidth=\skip126
```

```
\mdfboundingboxtotalwidth=\skip127
\mdfboundingboxheight=\skip128
\mdfboundingboxdepth=\skip129
\mdfboundingboxtotalheight=\skip130
\mdf@freevspace@length=\skip131
\mdf@horizontalwidthofbox@length=\skip132
\mdf@verticalmarginwhole@length=\skip133
\mdf@horizontalspaceofbox=\skip134
\mdfsubtitleheight=\skip135
\mdfsubsubtitleheight=\skip136
\c@mdfcountframes=\count267

****** mdframed patching \endmdf@trivlist

****** -- success******

\mdf@envdepth=\count268
\c@mdf@env@i=\count269
\c@mdf@env@ii=\count270
\c@mdf@zref@counter=\count271
Package zref Info: New property: mdf@pagevalue on input line 895.
) (c:/TeXLive/2022/texmf-dist/tex/latex/titlesec/titlesec.sty
Package: titlesec 2021/07/05 v2.14 Sectioning titles
\ttl@box=\box64
\beforetitleunit=\skip137
\aftertitleunit=\skip138
\ttl@plus=\dimen153
\ttl@minus=\dimen154
\ttl@toksa=\toks32
\titlewidth=\dimen155
\titlewidthlast=\dimen156
\titlewidthfirst=\dimen157
) (c:/TeXLive/2022/texmf-dist/tex/latex/koma-script/scrextend.sty
Package: scrextend 2022/10/12 v3.38 KOMA-Script package (extend other
classes w
ith features of KOMA-Script classes)
(c:/TeXLive/2022/texmf-dist/tex/latex/koma-script/scrkbase.sty
Package: scrkbase 2022/10/12 v3.38 KOMA-Script package (KOMA-Script-
dependent b
asics and keyval usage)
(c:/TeXLive/2022/texmf-dist/tex/latex/koma-script/scrbase.sty
Package: scrbase 2022/10/12 v3.38 KOMA-Script package (KOMA-Script-
independent
basics and keyval usage)
(c:/TeXLive/2022/texmf-dist/tex/latex/koma-script/scrlfile.sty
Package: scrlfile 2022/10/12 v3.38 KOMA-Script package (file load hooks)
(c:/TeXLive/2022/texmf-dist/tex/latex/koma-script/scrlfile-hook.sty
Package: scrlfile-hook 2022/10/12 v3.38 KOMA-Script package (using LaTeX
hooks)

(c:/TeXLive/2022/texmf-dist/tex/latex/koma-script/scrlogo.sty
Package: scrlogo 2022/10/12 v3.38 KOMA-Script package (logo)
)))
```

```
Applying: [2021/05/01] Usage of raw or classic option list on input line
252.
Already applied: [0000/00/00] Usage of raw or classic option list on
input line
 368.
))
Package scrextend Info: unexpected definition of `\@makefnmark'.
(scrextend)             Trying to patch it on input line 1709.
Package scrextend Info: patch seems to be successfull on input line 1709.
)

LaTeX Font Warning: Font shape `T1/cmr/m/n' in size <7.5> not available
(Font)              size <7> substituted on input line 65.

(c:/TeXLive/2022/texmf-dist/tex/latex/tools/calc.sty
Package: calc 2017/05/25 v4.3 Infix arithmetic (KKT,FJ)
\calc@Acount=\count272
\calc@Bcount=\count273
\calc@Adimen=\dimen158
\calc@Bdimen=\dimen159
\calc@Askip=\skip139
\calc@Bskip=\skip140
LaTeX Info: Redefining \setlength on input line 80.
LaTeX Info: Redefining \addtolength on input line 81.
\calc@Ccount=\count274
\calc@Cskip=\skip141
) (c:/TeXLive/2022/texmf-dist/tex/latex/geometry/geometry.sty
Package: geometry 2020/01/02 v5.9 Page Geometry
(c:/TeXLive/2022/texmf-dist/tex/generic/iftex/ifvtex.sty
Package: ifvtex 2019/10/25 v1.7 ifvtex legacy package. Use iftex instead.
)
\Gm@cnth=\count275
\Gm@cntv=\count276
\c@Gm@tempcnt=\count277
\Gm@bindingoffset=\dimen160
\Gm@wd@mp=\dimen161
\Gm@odd@mp=\dimen162
\Gm@even@mp=\dimen163
\Gm@layoutwidth=\dimen164
\Gm@layoutheight=\dimen165
\Gm@layouthoffset=\dimen166
\Gm@layoutvoffset=\dimen167
\Gm@dimlist=\toks33
) (c:/TeXLive/2022/texmf-dist/tex/latex/hyperref/hyperref.sty
Package: hyperref 2022-11-13 v7.00u Hypertext links for LaTeX
(c:/TeXLive/2022/texmf-dist/tex/generic/pdfescape/pdfescape.sty
Package: pdfescape 2019/12/09 v1.15 Implements pdfTeX's escape features
(HO)
) (c:/TeXLive/2022/texmf-dist/tex/latex/hycolor/hycolor.sty
Package: hycolor 2020-01-27 v1.10 Color options for hyperref/bookmark
(HO)
) (c:/TeXLive/2022/texmf-dist/tex/latex/letltxmacro/letltxmacro.sty
Package: letltxmacro 2019/12/03 v1.6 Let assignment for LaTeX macros (HO)
) (c:/TeXLive/2022/texmf-dist/tex/latex/hyperref/nameref.sty
```

```
Package: nameref 2022-05-17 v2.50 Cross-referencing by name of section
(c:/TeXLive/2022/texmf-dist/tex/latex/refcount/refcount.sty
Package: refcount 2019/12/15 v3.6 Data extraction from label references
(HO)
) (c:/TeXLive/2022/texmf-
dist/tex/generic/gettitlestring/gettitlestring.sty
Package: gettitlestring 2019/12/15 v1.6 Cleanup title references (HO)
)
\c@section@level=\count278
)
\@linkdim=\dimen168
\Hy@linkcounter=\count279
\Hy@pagecounter=\count280
(c:/TeXLive/2022/texmf-dist/tex/latex/hyperref/pd1enc.def
File: pd1enc.def 2022-11-13 v7.00u Hyperref: PDFDocEncoding definition
(HO)
Now handling font encoding PD1 ...
... no UTF-8 mapping file for font encoding PD1
) (c:/TeXLive/2022/texmf-dist/tex/generic/intcalc/intcalc.sty
Package: intcalc 2019/12/15 v1.3 Expandable calculations with integers
(HO)
)
\Hy@SavedSpaceFactor=\count281
(c:/TeXLive/2022/texmf-dist/tex/latex/hyperref/puenc.def
File: puenc.def 2022-11-13 v7.00u Hyperref: PDF Unicode definition (HO)
Now handling font encoding PU ...
... no UTF-8 mapping file for font encoding PU
)
Package hyperref Info: Option `colorlinks' set `true' on input line 4045.
Package hyperref Info: Hyper figures OFF on input line 4162.
Package hyperref Info: Link nesting OFF on input line 4167.
Package hyperref Info: Hyper index ON on input line 4170.
Package hyperref Info: Plain pages OFF on input line 4177.
Package hyperref Info: Backreferencing OFF on input line 4182.
Package hyperref Info: Implicit mode ON; LaTeX internals redefined.
Package hyperref Info: Bookmarks ON on input line 4410.
\c@Hy@tempcnt=\count282
LaTeX Info: Redefining \url on input line 4748.
\XeTeXLinkMargin=\dimen169
(c:/TeXLive/2022/texmf-dist/tex/generic/bitset/bitset.sty
Package: bitset 2019/12/09 v1.3 Handle bit-vector datatype (HO)
(c:/TeXLive/2022/texmf-dist/tex/generic/bigintcalc/bigintcalc.sty
Package: bigintcalc 2019/12/15 v1.5 Expandable calculations on big
integers (HO
)
))
\Fld@menulength=\count283
\Field@Width=\dimen170
\Fld@charsize=\dimen171
Package hyperref Info: Hyper figures OFF on input line 6027.
Package hyperref Info: Link nesting OFF on input line 6032.
Package hyperref Info: Hyper index ON on input line 6035.
Package hyperref Info: backreferencing OFF on input line 6042.
Package hyperref Info: Link coloring ON on input line 6045.
```

```
Package hyperref Info: Link coloring with OCG OFF on input line 6052.
Package hyperref Info: PDF/A mode OFF on input line 6057.
\Hy@abspage=\count284
\c@Item=\count285
\c@Hfootnote=\count286
)
Package hyperref Info: Driver (autodetected): hpdftex.
(c:/TeXLive/2022/texmf-dist/tex/latex/hyperref/hpdftex.def
File: hpdftex.def 2022-11-13 v7.00u Hyperref driver for pdfTeX
(c:/TeXLive/2022/texmf-dist/tex/latex/base/atveryend-ltx.sty
Package: atveryend-ltx 2020/08/19 v1.0a Emulation of the original
atveryend pac
kage
with kernel methods
)
\HyAnn@Count=\count287
\Fld@listcount=\count288
\c@bookmark@seq@number=\count289
(c:/TeXLive/2022/texmf-dist/tex/latex/rerunfilecheck/rerunfilecheck.sty
Package: rerunfilecheck 2022-07-10 v1.10 Rerun checks for auxiliary files
(HO)
(c:/TeXLive/2022/texmf-dist/tex/generic/uniquecounter/uniquecounter.sty
Package: uniquecounter 2019/12/15 v1.4 Provide unlimited unique counter
(HO)
)
Package uniquecounter Info: New unique counter `rerunfilecheck' on input
line 2
85.
)
\Hy@SectionHShift=\skip142
) (c:/TeXLive/2022/texmf-dist/tex/latex/preprint/authblk.sty
Package: authblk 2001/02/27 1.3 (PWD)
\affilsep=\skip143
\@affilsep=\skip144
\c@Maxaffil=\count290
\c@authors=\count291
\c@affil=\count292
) (c:/TeXLive/2022/texmf-dist/tex/latex/footmisc/footmisc.sty
Package: footmisc 2022/03/08 v6.0d a miscellany of footnote facilities
\FN@temptoken=\toks34
\footnotemargin=\dimen172
\@outputbox@depth=\dimen173
Package footmisc Info: Declaring symbol style bringhurst on input line
695.
Package footmisc Info: Declaring symbol style chicago on input line 703.
Package footmisc Info: Declaring symbol style wiley on input line 712.
Package footmisc Info: Declaring symbol style lamport-robust on input
line 723.

Package footmisc Info: Declaring symbol style lamport* on input line 743.
Package footmisc Info: Declaring symbol style lamport*-robust on input
line 764
.
) (c:/TeXLive/2022/texmf-dist/tex/latex/fancyhdr/fancyhdr.sty
```

```
Package: fancyhdr 2022/11/09 v4.1 Extensive control of page headers and
footers

\f@nch@headwidth=\skip145
\f@nch@O@elh=\skip146
\f@nch@O@erh=\skip147
\f@nch@O@olh=\skip148
\f@nch@O@orh=\skip149
\f@nch@O@elf=\skip150
\f@nch@O@erf=\skip151
\f@nch@O@olf=\skip152
\f@nch@O@orf=\skip153
) (c:/TeXLive/2022/texmf-dist/tex/generic/alphalph/alphalph.sty
Package: alphalph 2019/12/09 v2.6 Convert numbers to letters (HO)
)
\c@authorfn=\count293
(c:/TeXLive/2022/texmf-dist/tex/latex/abstract/abstract.sty
Package: abstract 2009/06/08 v1.2a configurable abstracts
\abstitleskip=\skip154
\absleftindent=\skip155
\absrightindent=\skip156
\absparindent=\skip157
\absparsep=\skip158
)
Package newfloat Info: New float `keypoints' with options
`placement=t!,name=kp
t' on input line 286.
\c@keypoints=\count294
\newfloat@ftype=\count295
Package newfloat Info: float type `keypoints'=8 on input line 286.
(c:/TeXLive/2022/texmf-dist/tex/latex/enumitem/enumitem.sty
Package: enumitem 2019/06/20 v3.9 Customized lists
\labelindent=\skip159
\enit@outerparindent=\dimen174
\enit@toks=\toks35
\enit@inbox=\box65
\enit@count@id=\count296
\enitdp@description=\count297
) (c:/TeXLive/2022/texmf-dist/tex/latex/quoting/quoting.sty
Package: quoting 2014/01/28 v0.1c Consolidated environment for displayed
text
\quo@toppartop=\skip160
) (c:/TeXLive/2022/texmf-dist/tex/latex/sttools/stfloats.sty
Package: stfloats 2017/03/27 v3.3 Improve float mechanism and
baselineskip sett
ings
\@dblbotnum=\count298
\c@dblbotnumber=\count299
) (c:/TeXLive/2022/texmf-dist/tex/latex/booktabs/booktabs.sty
Package: booktabs 2020/01/12 v1.61803398 Publication quality tables
\heavyrulewidth=\dimen175
\lightrulewidth=\dimen176
\cmidrulewidth=\dimen177
\belowrulesep=\dimen178
```

```
\belowbottomsep=\dimen179
\aboverulesep=\dimen180
\abovetopsep=\dimen181
\cmidrulesep=\dimen182
\cmidrulekern=\dimen183
\defaultaddspace=\dimen184
\@cmidla=\count300
\@cmidlb=\count301
\@aboverulesep=\dimen185
\@belowrulesep=\dimen186
\@thisruleclass=\count302
\@lastruleclass=\count303
\@thisrulewidth=\dimen187
) (c:/TeXLive/2022/texmf-dist/tex/latex/tools/tabularx.sty
Package: tabularx 2020/01/15 v2.11c `tabularx' package (DPC)
\TX@col@width=\dimen188
\TX@old@table=\dimen189
\TX@old@col=\dimen190
\TX@target=\dimen191
\TX@delta=\dimen192
\TX@cols=\count304
\TX@ftn=\toks36
)
\enitdp@tablenotes=\count305
(c:/TeXLive/2022/texmf-dist/tex/latex/caption/caption.sty
Package: caption 2022/03/01 v3.6b Customizing captions (AR)
(c:/TeXLive/2022/texmf-dist/tex/latex/caption/caption3.sty
Package: caption3 2022/03/17 v2.3b caption3 kernel (AR)
\caption@tempdima=\dimen193
\captionmargin=\dimen194
\caption@leftmargin=\dimen195
\caption@rightmargin=\dimen196
\caption@width=\dimen197
\caption@indent=\dimen198
\caption@parindent=\dimen199
\caption@hangindent=\dimen256
Package caption Info: Standard document class detected.
)
\c@caption@flags=\count306
\c@continuedfloat=\count307
Package caption Info: hyperref package is loaded.
Package caption Info: rotating package is loaded.
) (c:/TeXLive/2022/texmf-dist/tex/latex/natbib/natbib.sty
Package: natbib 2010/09/13 8.31b (PWD, AO)
\bibhang=\skip161
\bibsep=\skip162
LaTeX Info: Redefining \cite on input line 694.
\c@NAT@ctr=\count308
)) (c:/TeXLive/2022/texmf-dist/tex/latex/caption/subcaption.sty
Package: subcaption 2022/01/07 v1.5 Sub-captions (AR)
\c@subfigure=\count309
\c@subtable=\count310
\c@subkeypoints=\count311
) (c:/TeXLive/2022/texmf-dist/tex/latex/siunitx/siunitx.sty
```

```
Package: siunitx 2023-01-03 v3.2.0 A comprehensive (SI) units package
\l__siunitx_angle_tmp_dim=\dimen257
\l__siunitx_angle_marker_box=\box66
\l__siunitx_angle_unit_box=\box67
\l__siunitx_compound_count_int=\count312
(c:/TeXLive/2022/texmf-dist/tex/latex/translations/translations.sty
Package: translations 2022/02/05 v1.12 internationalization of LaTeX2e
packages
 (CN)
)
\l__siunitx_number_exponent_fixed_int=\count313
\l__siunitx_number_min_decimal_int=\count314
\l__siunitx_number_min_integer_int=\count315
\l__siunitx_number_round_precision_int=\count316
\l__siunitx_number_lower_threshold_int=\count317
\l__siunitx_number_upper_threshold_int=\count318
\l__siunitx_number_group_first_int=\count319
\l__siunitx_number_group_size_int=\count320
\l__siunitx_number_group_minimum_int=\count321
(c:/TeXLive/2022/texmf-dist/tex/latex/amsmath/amstext.sty
Package: amstext 2021/08/26 v2.01 AMS text
(c:/TeXLive/2022/texmf-dist/tex/latex/amsmath/amsgen.sty
File: amsgen.sty 1999/11/30 v2.0 generic functions
\@emptytoks=\toks37
\ex@=\dimen258
))
\l__siunitx_table_tmp_box=\box68
\l__siunitx_table_tmp_dim=\dimen259
\l__siunitx_table_column_width_dim=\dimen260
\l__siunitx_table_integer_box=\box69
\l__siunitx_table_decimal_box=\box70
\l__siunitx_table_uncert_box=\box71
\l__siunitx_table_before_box=\box72
\l__siunitx_table_after_box=\box73
\l__siunitx_table_before_dim=\dimen261
\l__siunitx_table_carry_dim=\dimen262
\l__siunitx_unit_tmp_int=\count322
\l__siunitx_unit_position_int=\count323
\l__siunitx_unit_total_int=\count324
)
Package translations Info: No language package found. I am going to use
`englis
h' as default language. on input line 50.
LaTeX Font Info:    Trying to load font information for T1+Merriwthr-OsF
on inp
ut line 50.
(c:/TeXLive/2022/texmf-dist/tex/latex/merriweather/T1Merriwthr-OsF.fd
File: T1Merriwthr-OsF.fd 2020/08/30 (autoinst) Font definitions for
T1/Merriwth
r-OsF.
)
LaTeX Font Info:    Font shape `T1/Merriwthr-OsF/m/n' will be
(Font)              scaled to size 7.5pt on input line 50.
(c:/TeXLive/2022/texmf-dist/tex/latex/l3backend/l3backend-pdftex.def
```

```
File: l3backend-pdftex.def 2023-01-16 L3 backend support: PDF output
(pdfTeX)
\l__color_backend_stack_int=\count325
\l__pdf_internal_box=\box74
) (./main.aux)
\openout1 = `main.aux'.

LaTeX Font Info:    Checking defaults for OML/cmm/m/it on input line 50.
LaTeX Font Info:    ... okay on input line 50.
LaTeX Font Info:    Checking defaults for OMS/cmsy/m/n on input line 50.
LaTeX Font Info:    ... okay on input line 50.
LaTeX Font Info:    Checking defaults for OT1/cmr/m/n on input line 50.
LaTeX Font Info:    ... okay on input line 50.
LaTeX Font Info:    Checking defaults for T1/cmr/m/n on input line 50.
LaTeX Font Info:    ... okay on input line 50.
LaTeX Font Info:    Checking defaults for TS1/cmr/m/n on input line 50.
LaTeX Font Info:    ... okay on input line 50.
LaTeX Font Info:    Checking defaults for OMX/cmex/m/n on input line 50.
LaTeX Font Info:    ... okay on input line 50.
LaTeX Font Info:    Checking defaults for U/cmr/m/n on input line 50.
LaTeX Font Info:    ... okay on input line 50.
LaTeX Font Info:    Checking defaults for PD1/pdf/m/n on input line 50.
LaTeX Font Info:    ... okay on input line 50.
LaTeX Font Info:    Checking defaults for PU/pdf/m/n on input line 50.
LaTeX Font Info:    ... okay on input line 50.
LaTeX Info: Redefining \microtypecontext on input line 50.
Package microtype Info: Applying patch `item' on input line 50.
Package microtype Info: Applying patch `toc' on input line 50.
Package microtype Info: Applying patch `eqnum' on input line 50.

Package microtype Warning: Unable to apply patch `footnote' on input line
50.

Package microtype Info: Generating PDF output.
Package microtype Info: Character protrusion enabled (level 2).
Package microtype Info: Using default protrusion set `alltext'.
Package microtype Info: Automatic font expansion enabled (level 2),
(microtype)             stretch: 20, shrink: 20, step: 1, non-selected.
Package microtype Info: Using default expansion set `alltext-nott'.
LaTeX Info: Redefining \showhyphens on input line 50.
Package microtype Info: No adjustment of tracking.
Package microtype Info: No adjustment of interword spacing.
Package microtype Info: No adjustment of character kerning.
Package microtype Info: Loading generic protrusion settings for font
family
(microtype)             `Merriwthr-OsF' (encoding: T1).
(microtype)             For optimal results, create family-specific
settings.
(microtype)             See the microtype manual for details.
LaTeX Font Info:    Redeclaring symbol font `operators' on input line 50.
LaTeX Font Info:    Encoding `OT1' has changed to `T1' for symbol font
(Font)              `operators' in the math version `normal' on input
line 50.
```

```
LaTeX Font Info:    Overwriting symbol font `operators' in version
`normal'
(Font)                  OT1/cmr/m/n --> T1/Merriwthr-OsF/m/up on input
line 50.

LaTeX Font Info:    Encoding `OT1' has changed to `T1' for symbol font
(Font)              `operators' in the math version `bold' on input line
50.
LaTeX Font Info:    Overwriting symbol font `operators' in version `bold'
(Font)                  OT1/cmr/bx/n --> T1/Merriwthr-OsF/m/up on input
line 50
.
LaTeX Font Info:    Overwriting symbol font `operators' in version `bold'
(Font)                  T1/Merriwthr-OsF/m/up --> T1/Merriwthr-OsF/b/up
on inpu
t line 50.
LaTeX Font Info:    Redeclaring math alphabet \mathbf on input line 50.
LaTeX Font Info:    Overwriting math alphabet `\mathbf' in version
`normal'
(Font)                  OT1/cmr/bx/n --> T1/Merriwthr-OsF/b/up on input
line 50
.
LaTeX Font Info:    Overwriting math alphabet `\mathbf' in version `bold'
(Font)                  OT1/cmr/bx/n --> T1/Merriwthr-OsF/b/up on input
line 50
.
LaTeX Font Info:    Redeclaring math alphabet \mathsf on input line 50.
LaTeX Font Info:    Overwriting math alphabet `\mathsf' in version
`normal'
(Font)                  OT1/cmss/m/n --> T1/MerriwthrSans-OsF/m/up on
input lin
e 50.
LaTeX Font Info:    Overwriting math alphabet `\mathsf' in version `bold'
(Font)                  OT1/cmss/bx/n --> T1/MerriwthrSans-OsF/m/up on
input li
ne 50.
LaTeX Font Info:    Redeclaring math alphabet \mathit on input line 50.
LaTeX Font Info:    Overwriting math alphabet `\mathit' in version
`normal'
(Font)                  OT1/cmr/m/it --> T1/Merriwthr-OsF/m/it on input
line 50
.
LaTeX Font Info:    Overwriting math alphabet `\mathit' in version `bold'
(Font)                  OT1/cmr/bx/it --> T1/Merriwthr-OsF/m/it on input
line 5
0.
LaTeX Font Info:    Redeclaring math alphabet \mathtt on input line 50.
LaTeX Font Info:    Overwriting math alphabet `\mathtt' in version
`normal'
(Font)                  OT1/cmtt/m/n --> T1/lmtt/m/up on input line 50.
LaTeX Font Info:    Overwriting math alphabet `\mathtt' in version `bold'
(Font)                  OT1/cmtt/m/n --> T1/lmtt/m/up on input line 50.
LaTeX Font Info:    Overwriting math alphabet `\mathsf' in version `bold'
```

```
(Font)                  T1/MerriwthrSans-OsF/m/up --> T1/MerriwthrSans-
OsF/b/up
 on input line 50.
LaTeX Font Info:    Overwriting math alphabet `\mathit' in version `bold'
(Font)                  T1/Merriwthr-OsF/m/it --> T1/Merriwthr-OsF/b/it
on inpu
t line 50.
\c@mv@tabular=\count326
\c@mv@boldtabular=\count327
(c:/TeXLive/2022/texmf-dist/tex/context/base/mkii/supp-pdf.mkii
[Loading MPS to PDF converter (version 2006.09.02).]
\scratchcounter=\count328
\scratchdimen=\dimen263
\scratchbox=\box75
\nofMPsegments=\count329
\nofMParguments=\count330
\everyMPshowfont=\toks38
\MPscratchCnt=\count331
\MPscratchDim=\dimen264
\MPnumerator=\count332
\makeMPintoPDFobject=\count333
\everyMPtoPDFconversion=\toks39
) (c:/TeXLive/2022/texmf-dist/tex/latex/epstopdf-pkg/epstopdf-base.sty
Package: epstopdf-base 2020-01-24 v2.11 Base part for package epstopdf
Package epstopdf-base Info: Redefining graphics rule for `.eps' on input
line 4
85.
(c:/TeXLive/2022/texmf-dist/tex/latex/latexconfig/epstopdf-sys.cfg
File: epstopdf-sys.cfg 2010/07/13 v1.3 Configuration of (r)epstopdf for
TeX Liv
e
))
Package lastpage Info: Please have a look at the pageslts package at
(lastpage)             https://www.ctan.org/pkg/pageslts
(lastpage)             ! on input line 50.
*geometry* driver: auto-detecting
*geometry* detected driver: pdftex
*geometry* verbose mode - [ preamble ] result:
* driver: pdftex
* paper: a4paper
* layout: <same size as paper>
* layoutoffset:(h,v)=(0.0pt,0.0pt)
* modes: includefoot twoside
* h-part:(L,W,R)=(54.64pt, 488.22787pt, 54.64pt)
* v-part:(T,H,B)=(66.0pt, 745.04684pt, 34.0pt)
* \paperwidth=597.50787pt
* \paperheight=845.04684pt
* \textwidth=488.22787pt
* \textheight=715.04684pt
* \oddsidemargin=-17.62999pt
* \evensidemargin=-17.62999pt
* \topmargin=-47.76999pt
* \headheight=17.5pt
* \headsep=24.0pt
```

```
* \topskip=10.0pt
* \footskip=30.0pt
* \marginparwidth=48.0pt
* \marginparsep=10.0pt
* \columnsep=18.0pt
* \skip\footins=22.0pt plus 2.0pt
* \hoffset=0.0pt
* \voffset=0.0pt
* \mag=1000
* \@twocolumntrue
* \@twosidetrue
* \@mparswitchtrue
* \@reversemarginfalse
* (1in=72.27pt=25.4mm, 1cm=28.453pt)

Package hyperref Info: Link coloring ON on input line 50.
(./main.out) (./main.out)
\@outlinefile=\write3
\openout3 = `main.out'.

\@gscitedetails=\box76
\@gscitedetailsheight=\skip163
\@gsheadbox=\box77
\@gsheadboxheight=\skip164
LaTeX Font Info:    Font shape `T1/Merriwthr-OsF/b/n' will be
(Font)              scaled to size 6.5pt on input line 50.
LaTeX Font Info:    Calculating math sizes for size <7.5> on input line
50.

LaTeX Font Warning: Font shape `T1/Merriwthr-OsF/m/up' undefined
(Font)              using `T1/Merriwthr-OsF/m/n' instead on input line
50.

LaTeX Font Info:    Font shape `T1/Merriwthr-OsF/m/up' will be
(Font)              scaled to size 6.24973pt on input line 50.
LaTeX Font Info:    Font shape `T1/Merriwthr-OsF/m/up' will be
(Font)              scaled to size 5.24997pt on input line 50.
LaTeX Font Info:    Trying to load font information for U+eur on input
line 50.

(c:/TeXLive/2022/texmf-dist/tex/latex/amsfonts/ueur.fd
File: ueur.fd 2013/01/14 v3.01 Euler Roman
) (c:/TeXLive/2022/texmf-dist/tex/latex/microtype/mt-eur.cfg
File: mt-eur.cfg 2006/07/31 v1.1 microtype config. file: AMS Euler Roman
(RS)
)

LaTeX Font Warning: Font shape `OMS/cmsy/m/n' in size <7.5> not available
(Font)              size <7> substituted on input line 50.

LaTeX Font Info:    External font `cmex10' loaded for size
(Font)              <7.5> on input line 50.
LaTeX Font Info:    External font `cmex10' loaded for size
(Font)              <6.24973> on input line 50.
```

```
LaTeX Font Info:    External font `cmex10' loaded for size
(Font)              <5.24997> on input line 50.
LaTeX Font Info:    Trying to load font information for U+euf on input
line 50.

(c:/TeXLive/2022/texmf-dist/tex/latex/amsfonts/ueuf.fd
File: ueuf.fd 2013/01/14 v3.01 Euler Fraktur
) (c:/TeXLive/2022/texmf-dist/tex/latex/microtype/mt-euf.cfg
File: mt-euf.cfg 2006/07/03 v1.1 microtype config. file: AMS Euler
Fraktur (RS)

)
LaTeX Font Info:    Trying to load font information for U+eus on input
line 50.

(c:/TeXLive/2022/texmf-dist/tex/latex/amsfonts/ueus.fd
File: ueus.fd 2013/01/14 v3.01 Euler Script
) (c:/TeXLive/2022/texmf-dist/tex/latex/microtype/mt-eus.cfg
File: mt-eus.cfg 2006/07/28 v1.2 microtype config. file: AMS Euler Script
(RS)
)
LaTeX Font Info:    Trying to load font information for U+euex on input
line 50
.
(c:/TeXLive/2022/texmf-dist/tex/latex/amsfonts/ueuex.fd
File: ueuex.fd 2013/01/14 v3.01 Euler extra symbols
)

LaTeX Font Warning: Font shape `OML/cmm/m/it' in size <7.5> not available
(Font)              size <7> substituted on input line 50.

LaTeX Font Info:    Font shape `T1/Merriwthr-OsF/m/n' will be
(Font)              scaled to size 6.24973pt on input line 50.
LaTeX Font Info:    Font shape `T1/Merriwthr-OsF/m/n' will be
(Font)              scaled to size 5.24997pt on input line 50.
LaTeX Font Info:    Font shape `T1/Merriwthr-OsF/m/it' will be
(Font)              scaled to size 7.5pt on input line 50.
LaTeX Font Info:    Font shape `T1/Merriwthr-OsF/m/it' will be
(Font)              scaled to size 6.24973pt on input line 50.
LaTeX Font Info:    Font shape `T1/Merriwthr-OsF/m/it' will be
(Font)              scaled to size 5.24997pt on input line 50.
LaTeX Font Info:    Font shape `T1/Merriwthr-OsF/m/n' will be
(Font)              scaled to size 8.0pt on input line 50.
LaTeX Font Info:    Font shape `T1/Merriwthr-OsF/m/it' will be
(Font)              scaled to size 8.0pt on input line 50.
LaTeX Font Info:    Font shape `T1/Merriwthr-OsF/b/it' will be
(Font)              scaled to size 8.0pt on input line 50.
Package caption Info: Begin \AtBeginDocument code.
Package caption Info: End \AtBeginDocument code.

(c:/TeXLive/2022/texmf-dist/tex/latex/translations/translations-basic-
dictionar
y-english.trsl
```

```
File: translations-basic-dictionary-english.trsl (english translation
file `tra
nslations-basic-dictionary')
)
Package translations Info: loading dictionary `translations-basic-
dictionary' f
or `english'. on input line 50.
TextBlockOrigin set to 4pc+6.64pt x 4pc+6pt
<oup.pdf, id=85, 49.18375pt x 48.18pt>
File: oup.pdf Graphic file (type pdf)
<use oup.pdf>
Package pdftex.def Info: oup.pdf  used on input line 61.
(pdftex.def)             Requested size: 59.24683pt x 58.038pt.
<gigascience-logo.pdf, id=86, 99.37125pt x 33.12375pt>
File: gigascience-logo.pdf Graphic file (type pdf)
<use gigascience-logo.pdf>
Package pdftex.def Info: gigascience-logo.pdf  used on input line 61.
(pdftex.def)             Requested size: 126.00902pt x 42.0pt.

Overfull \hbox (54.64pt too wide) in paragraph at lines 61--61
[][]
 []

LaTeX Font Info:    Font shape `T1/Merriwthr-OsF/m/n' will be
(Font)              scaled to size 14.0pt on input line 61.
LaTeX Font Info:    Font shape `T1/Merriwthr-OsF/m/n' will be
(Font)              scaled to size 8.99997pt on input line 61.
LaTeX Font Info:    Calculating math sizes for size <14> on input line
61.
LaTeX Font Info:    Font shape `T1/Merriwthr-OsF/m/up' will be
(Font)              scaled to size 14.0pt on input line 61.
LaTeX Font Info:    Font shape `T1/Merriwthr-OsF/m/up' will be
(Font)              scaled to size 11.66617pt on input line 61.
LaTeX Font Info:    Font shape `T1/Merriwthr-OsF/m/up' will be
(Font)              scaled to size 9.79996pt on input line 61.
LaTeX Font Info:    External font `cmex10' loaded for size
(Font)              <14> on input line 61.
LaTeX Font Info:    External font `cmex10' loaded for size
(Font)              <11.66617> on input line 61.
LaTeX Font Info:    External font `cmex10' loaded for size
(Font)              <9.79996> on input line 61.
LaTeX Font Info:    Font shape `T1/Merriwthr-OsF/m/n' will be
(Font)              scaled to size 11.66617pt on input line 61.
LaTeX Font Info:    Font shape `T1/Merriwthr-OsF/m/n' will be
(Font)              scaled to size 9.79996pt on input line 61.
LaTeX Font Info:    Font shape `T1/Merriwthr-OsF/m/it' will be
(Font)              scaled to size 14.0pt on input line 61.
LaTeX Font Info:    Font shape `T1/Merriwthr-OsF/m/it' will be
(Font)              scaled to size 11.66617pt on input line 61.
LaTeX Font Info:    Font shape `T1/Merriwthr-OsF/m/it' will be
(Font)              scaled to size 9.79996pt on input line 61.
LaTeX Font Info:    Font shape `T1/Merriwthr-OsF/b/n' will be
(Font)              scaled to size 18.0pt on input line 61.
LaTeX Font Info:    Font shape `T1/Merriwthr-OsF/m/n' will be
```

```
(Font)              scaled to size 13.0pt on input line 61.
LaTeX Font Info:    Calculating math sizes for size <13> on input line
61.
LaTeX Font Info:    Font shape `T1/Merriwthr-OsF/m/up' will be
(Font)              scaled to size 13.0pt on input line 61.
LaTeX Font Info:    Font shape `T1/Merriwthr-OsF/m/up' will be
(Font)              scaled to size 10.83287pt on input line 61.
LaTeX Font Info:    Font shape `T1/Merriwthr-OsF/m/up' will be
(Font)              scaled to size 9.09996pt on input line 61.

LaTeX Font Warning: Font shape `OMS/cmsy/m/n' in size <13> not available
(Font)              size <12> substituted on input line 61.

LaTeX Font Info:    External font `cmex10' loaded for size
(Font)              <13> on input line 61.
LaTeX Font Info:    External font `cmex10' loaded for size
(Font)              <10.83287> on input line 61.
LaTeX Font Info:    External font `cmex10' loaded for size
(Font)              <9.09996> on input line 61.

LaTeX Font Warning: Font shape `OML/cmm/m/it' in size <13> not available
(Font)              size <12> substituted on input line 61.

LaTeX Font Info:    Font shape `T1/Merriwthr-OsF/m/n' will be
(Font)              scaled to size 10.83287pt on input line 61.
LaTeX Font Info:    Font shape `T1/Merriwthr-OsF/m/n' will be
(Font)              scaled to size 9.09996pt on input line 61.
LaTeX Font Info:    Font shape `T1/Merriwthr-OsF/m/it' will be
(Font)              scaled to size 13.0pt on input line 61.
LaTeX Font Info:    Font shape `T1/Merriwthr-OsF/m/it' will be
(Font)              scaled to size 10.83287pt on input line 61.
LaTeX Font Info:    Font shape `T1/Merriwthr-OsF/m/it' will be
(Font)              scaled to size 9.09996pt on input line 61.
LaTeX Font Info:    Trying to load font information for TS1+Merriwthr-OsF
on in
put line 61.
(c:/TeXLive/2022/texmf-dist/tex/latex/merriweather/TS1Merriwthr-OsF.fd
File: TS1Merriwthr-OsF.fd 2020/08/30 (autoinst) Font definitions for
TS1/Merriw
thr-OsF.
)
LaTeX Font Info:    Font shape `TS1/Merriwthr-OsF/m/n' will be
(Font)              scaled to size 10.83287pt on input line 61.
Package microtype Info: Loading generic protrusion settings for font
family
(microtype)             `Merriwthr-OsF' (encoding: TS1).
(microtype)             For optimal results, create family-specific
settings.
(microtype)             See the microtype manual for details.
LaTeX Font Info:    Font shape `T1/Merriwthr-OsF/m/n' will be
(Font)              scaled to size 9.0pt on input line 61.
LaTeX Font Info:    Font shape `T1/Merriwthr-OsF/m/up' will be
(Font)              scaled to size 9.0pt on input line 61.
LaTeX Font Info:    Font shape `T1/Merriwthr-OsF/m/up' will be
```

```
(Font)              scaled to size 7.0pt on input line 61.
LaTeX Font Info:    Font shape `T1/Merriwthr-OsF/m/up' will be
(Font)              scaled to size 5.0pt on input line 61.
LaTeX Font Info:    External font `cmex10' loaded for size
(Font)              <9> on input line 61.
LaTeX Font Info:    External font `cmex10' loaded for size
(Font)              <7> on input line 61.
LaTeX Font Info:    External font `cmex10' loaded for size
(Font)              <5> on input line 61.
LaTeX Font Info:    Font shape `T1/Merriwthr-OsF/m/n' will be
(Font)              scaled to size 7.0pt on input line 61.
LaTeX Font Info:    Font shape `T1/Merriwthr-OsF/m/n' will be
(Font)              scaled to size 5.0pt on input line 61.
LaTeX Font Info:    Font shape `T1/Merriwthr-OsF/m/it' will be
(Font)              scaled to size 9.0pt on input line 61.
LaTeX Font Info:    Font shape `T1/Merriwthr-OsF/m/it' will be
(Font)              scaled to size 7.0pt on input line 61.
LaTeX Font Info:    Font shape `T1/Merriwthr-OsF/m/it' will be
(Font)              scaled to size 5.0pt on input line 61.
LaTeX Font Info:    Font shape `T1/Merriwthr-OsF/m/n' will be
(Font)              scaled to size 6.5pt on input line 61.
LaTeX Font Info:    Calculating math sizes for size <6.5> on input line
61.
LaTeX Font Info:    Font shape `T1/Merriwthr-OsF/m/up' will be
(Font)              scaled to size 6.5pt on input line 61.
LaTeX Font Info:    Font shape `T1/Merriwthr-OsF/m/up' will be
(Font)              scaled to size 5.41643pt on input line 61.
LaTeX Font Info:    Font shape `T1/Merriwthr-OsF/m/up' will be
(Font)              scaled to size 4.54997pt on input line 61.

LaTeX Font Warning: Font shape `OMS/cmsy/m/n' in size <6.5> not available
(Font)              size <6> substituted on input line 61.


LaTeX Font Warning: Font shape `OMS/cmsy/m/n' in size <5.41643> not
available
(Font)              size <5> substituted on input line 61.


LaTeX Font Warning: Font shape `OMS/cmsy/m/n' in size <4.54997> not
available
(Font)              size <5> substituted on input line 61.

LaTeX Font Info:    External font `cmex10' loaded for size
(Font)              <6.5> on input line 61.
LaTeX Font Info:    External font `cmex10' loaded for size
(Font)              <5.41643> on input line 61.
LaTeX Font Info:    External font `cmex10' loaded for size
(Font)              <4.54997> on input line 61.

LaTeX Font Warning: Font shape `OML/cmm/m/it' in size <6.5> not available
(Font)              size <6> substituted on input line 61.
```

```
LaTeX Font Warning: Font shape `OML/cmm/m/it' in size <5.41643> not
available
(Font)              size <5> substituted on input line 61.


LaTeX Font Warning: Font shape `OML/cmm/m/it' in size <4.54997> not
available
(Font)              size <5> substituted on input line 61.

LaTeX Font Info:    Font shape `T1/Merriwthr-OsF/m/n' will be
(Font)              scaled to size 5.41643pt on input line 61.
LaTeX Font Info:    Font shape `T1/Merriwthr-OsF/m/n' will be
(Font)              scaled to size 4.54997pt on input line 61.
LaTeX Font Info:    Font shape `T1/Merriwthr-OsF/m/it' will be
(Font)              scaled to size 6.5pt on input line 61.
LaTeX Font Info:    Font shape `T1/Merriwthr-OsF/m/it' will be
(Font)              scaled to size 5.41643pt on input line 61.
LaTeX Font Info:    Font shape `T1/Merriwthr-OsF/m/it' will be
(Font)              scaled to size 4.54997pt on input line 61.
LaTeX Font Info:    Font shape `TS1/Merriwthr-OsF/m/n' will be
(Font)              scaled to size 5.41643pt on input line 61.

Overfull \hbox (54.64pt too wide) in paragraph at lines 61--61
[][][]
 []

LaTeX Font Info:    Font shape `T1/Merriwthr-OsF/b/n' will be
(Font)              scaled to size 10.0pt on input line 61.
LaTeX Font Info:    Font shape `T1/Merriwthr-OsF/b/n' will be
(Font)              scaled to size 8.0pt on input line 61.
LaTeX Font Info:    Trying to load font information for T1+lmtt on input
line 6
1.
(c:/TeXLive/2022/texmf-dist/tex/latex/lm/t1lmtt.fd
File: t1lmtt.fd 2015/05/01 v1.6.1 Font defs for Latin Modern
)
Package microtype Info: Loading generic protrusion settings for font
family
(microtype)             `lmtt' (encoding: T1).
(microtype)             For optimal results, create family-specific
settings.
(microtype)             See the microtype manual for details.
LaTeX Font Info:    Font shape `T1/Merriwthr-OsF/m/up' will be
(Font)              scaled to size 8.0pt on input line 61.
LaTeX Font Info:    Font shape `T1/Merriwthr-OsF/m/up' will be
(Font)              scaled to size 6.0pt on input line 61.
LaTeX Font Info:    External font `cmex10' loaded for size
(Font)              <8> on input line 61.
LaTeX Font Info:    External font `cmex10' loaded for size
(Font)              <6> on input line 61.
LaTeX Font Info:    Font shape `T1/Merriwthr-OsF/m/n' will be
(Font)              scaled to size 6.0pt on input line 61.
LaTeX Font Info:    Font shape `T1/Merriwthr-OsF/m/it' will be
(Font)              scaled to size 6.0pt on input line 61.
```

```
Overfull \hbox (54.64pt too wide) in paragraph at lines 61--61
[][][]
 []

Package mdframed Info: mdframed works in twoside mode on input line 64.
LaTeX Font Info:    Font shape `T1/Merriwthr-OsF/b/n' will be
(Font)              scaled to size 8.2pt on input line 64.
LaTeX Font Info:    Font shape `TS1/Merriwthr-OsF/m/n' will be
(Font)              scaled to size 7.5pt on input line 66.
Package mdframed Info: mdframed inside float
 mdframed uses option nobreak mdframed on input line 70.
Package mdframed Info: mdframed inside a box
 mdframed uses option nobreak mdframed on input line 70.

Underfull \vbox (badness 10000) has occurred while \output is active []


Underfull \vbox (badness 4531) has occurred while \output is active []

LaTeX Font Info:    Font shape `T1/Merriwthr-OsF/m/n' will be
(Font)              scaled to size 7.8pt on input line 76.
LaTeX Font Info:    Font shape `T1/Merriwthr-OsF/b/n' will be
(Font)              scaled to size 7.8pt on input line 76.
[1{c:/TeXLive/2022/texmf-var/fonts/map/pdftex/updmap/pdftex.map}


 <./oup.pdf> <./gigascience-logo.pdf>]
<Figure1_Main.png, id=116, 425.6703pt x 292.6935pt>
File: Figure1_Main.png Graphic file (type png)
<use Figure1_Main.png>
Package pdftex.def Info: Figure1_Main.png  used on input line 80.
(pdftex.def)             Requested size: 235.11394pt x 161.6697pt.
LaTeX Font Info:    Font shape `T1/Merriwthr-OsF/b/n' will be
(Font)              scaled to size 6.0pt on input line 81.
<Figure2_Main.png, id=121, 849.8952pt x 486.3771pt>
File: Figure2_Main.png Graphic file (type png)
<use Figure2_Main.png>
Package pdftex.def Info: Figure2_Main.png  used on input line 88.
(pdftex.def)             Requested size: 212.47328pt x 121.59398pt.

Underfull \hbox (badness 1496) in paragraph at lines 85--92
[]\T1/Merriwthr-OsF/m/n/7.5 (+20) It is de-signed as a web-based ap-pli-
ca-tion
 us-ing PHP and
 []


Underfull \vbox (badness 1371) has occurred while \output is active []

LaTeX Font Info:    Font shape `T1/Merriwthr-OsF/b/n' will be
(Font)              scaled to size 8.5pt on input line 95.
<Figure3_Main.png, id=124, 465.23813pt x 352.81813pt>
File: Figure3_Main.png Graphic file (type png)
```

```
<use Figure3_Main.png>
Package pdftex.def Info: Figure3_Main.png  used on input line 98.
(pdftex.def)             Requested size: 232.61848pt x 176.40863pt.
<Figure4_Main.png, id=127, 460.72125pt x 304.63812pt>
File: Figure4_Main.png Graphic file (type png)
<use Figure4_Main.png>
Package pdftex.def Info: Figure4_Main.png  used on input line 104.
(pdftex.def)             Requested size: 230.36006pt x 152.31868pt.
LaTeX Font Info:    Font shape `T1/Merriwthr-OsF/m/it' will be
(Font)              scaled to size 7.8pt on input line 107.
[2 <./Figure1_Main.png> <./Figure2_Main.png> <./Figure3_Main.png>]
<Figure5A_Main.png, id=140, 462.72874pt x 248.93pt>
File: Figure5A_Main.png Graphic file (type png)
<use Figure5A_Main.png>
Package pdftex.def Info: Figure5A_Main.png  used on input line 112.
(pdftex.def)             Requested size: 219.70105pt x 118.18979pt.
LaTeX Font Info:    Font shape `T1/Merriwthr-OsF/m/n' will be
(Font)              scaled to size 6.8438pt on input line 113.
LaTeX Font Info:    Font shape `T1/Merriwthr-OsF/b/n' will be
(Font)              scaled to size 6.8438pt on input line 113.
<Figure5B_Main.png, id=141, 463.7325pt x 179.67125pt>
File: Figure5B_Main.png Graphic file (type png)
<use Figure5B_Main.png>
Package pdftex.def Info: Figure5B_Main.png  used on input line 118.
(pdftex.def)             Requested size: 219.70105pt x 85.12265pt.
<Figure6A_Main.png, id=154, 307.64937pt x 245.91875pt>
File: Figure6A_Main.png Graphic file (type png)
<use Figure6A_Main.png>
Package pdftex.def Info: Figure6A_Main.png  used on input line 133.
(pdftex.def)             Requested size: 219.70105pt x 175.62042pt.
<Figure6B_Main.png, id=155, 307.1475pt x 403.5075pt>
File: Figure6B_Main.png Graphic file (type png)
<use Figure6B_Main.png>
Package pdftex.def Info: Figure6B_Main.png  used on input line 139.
(pdftex.def)             Requested size: 219.70105pt x 288.62881pt.
<Figure7A_Main.png, id=160, 408.02437pt x 370.88562pt>
File: Figure7A_Main.png Graphic file (type png)
<use Figure7A_Main.png>
Package pdftex.def Info: Figure7A_Main.png  used on input line 156.
(pdftex.def)             Requested size: 219.70105pt x 199.70366pt.
<Figure7B_Main.png, id=161, 315.1775pt x 96.36pt>
File: Figure7B_Main.png Graphic file (type png)
<use Figure7B_Main.png>
Package pdftex.def Info: Figure7B_Main.png  used on input line 162.
(pdftex.def)             Requested size: 219.70105pt x 67.17068pt.

Underfull \vbox (badness 10000) has occurred while \output is active []

[3 <./Figure4_Main.png> <./Figure5A_Main.png> <./Figure5B_Main.png>]
Underfull \vbox (badness 4291) has occurred while \output is active []

[4 <./Figure6A_Main.png> <./Figure6B_Main.png> <./Figure7A_Main.png>
<./Figure7
B_Main.png>]
```

```
LaTeX Font Info:    Font shape `T1/Merriwthr-OsF/m/up' will be
(Font)              scaled to size 7.5pt on input line 177.

Underfull \hbox (badness 1178) in paragraph at lines 184--184
|\T1/Merriwthr-OsF/b/n/10 (+20) Availability of Sup-port-ing Code and Re-
quire-

 []


Underfull \vbox (badness 10000) has occurred while \output is active []


Underfull \hbox (badness 10000) in paragraph at lines 193--194
[]\T1/Merriwthr-OsF/m/up/7.5 (+20) Source code: [][]$\T1/lmtt/m/n/7.5
https : /
 / github . com / metabolomicsworkbench /
 []

[5] (./main.bbl
Underfull \hbox (badness 1014) in paragraph at lines 86--90
\T1/Merriwthr-OsF/m/up/7.5 (+20) search 2022;50(1):D988--D995.
[][]$\T1/lmtt/m
/n/7.5 https : / / doi . org / 10 . 1093 /
 []

)
AED: lastpage setting LastPage
[6] (./main.aux)

LaTeX Font Warning: Size substitutions with differences
(Font)              up to 1.0pt have occurred.


LaTeX Font Warning: Some font shapes were not available, defaults
substituted.

Package rerunfilecheck Info: File `main.out' has not changed.
(rerunfilecheck)             Checksum:
CA45DC1EDD43D0B7FCD9997625FD1F16;3388.
 )
Here is how much of TeX's memory you used:
 23867 strings out of 476093
 465594 string characters out of 5795582
 1888787 words of memory out of 5000000
 43302 multiletter control sequences out of 15000+600000
 1792588 words of font info for 477 fonts, out of 8000000 for 9000
 1141 hyphenation exceptions out of 8191
 123i,12n,131p,2930b,935s stack positions out of
10000i,1000n,20000p,200000b,200000s
{c:/TeXLive/2022/texmf-
dist/fonts/enc/dvips/merriweather/merriwthr_posqbl.enc
}{c:/TeXLive/2022/texmf-dist/fonts/enc/dvips/lm/lm-
ec.enc}{c:/TeXLive/2022/texm
```

```
f-
dist/fonts/enc/dvips/merriweather/merriwthr_owzwzj.enc}<c:/TeXLive/2022/t
exmf
-dist/fonts/type1/sorkin/merriweather/Merriwthr-
Bold.pfb><c:/TeXLive/2022/texmf
-dist/fonts/type1/sorkin/merriweather/Merriwthr-
BoldItalic.pfb><c:/TeXLive/2022
/texmf-dist/fonts/type1/sorkin/merriweather/Merriwthr-
Italic.pfb><c:/TeXLive/20
22/texmf-dist/fonts/type1/sorkin/merriweather/Merriwthr-
Regular.pfb><c:/TeXLive
/2022/texmf-
dist/fonts/type1/public/amsfonts/euler/euex8.pfb><c:/TeXLive/2022/t
exmf-dist/fonts/type1/public/lm/lmtt8.pfb>
Output written on main.pdf (6 pages, 1068553 bytes).
PDF statistics:
 275 PDF objects out of 1000 (max. 8388607)
 228 compressed objects within 3 object streams
 53 named destinations out of 1000 (max. 500000)
 195805 words of extra memory for PDF output out of 221844 (max.
10000000)
```

Click here to access/download;LaTeX - Figure (eps, ps, etc.);example-grid-100x100pt.png

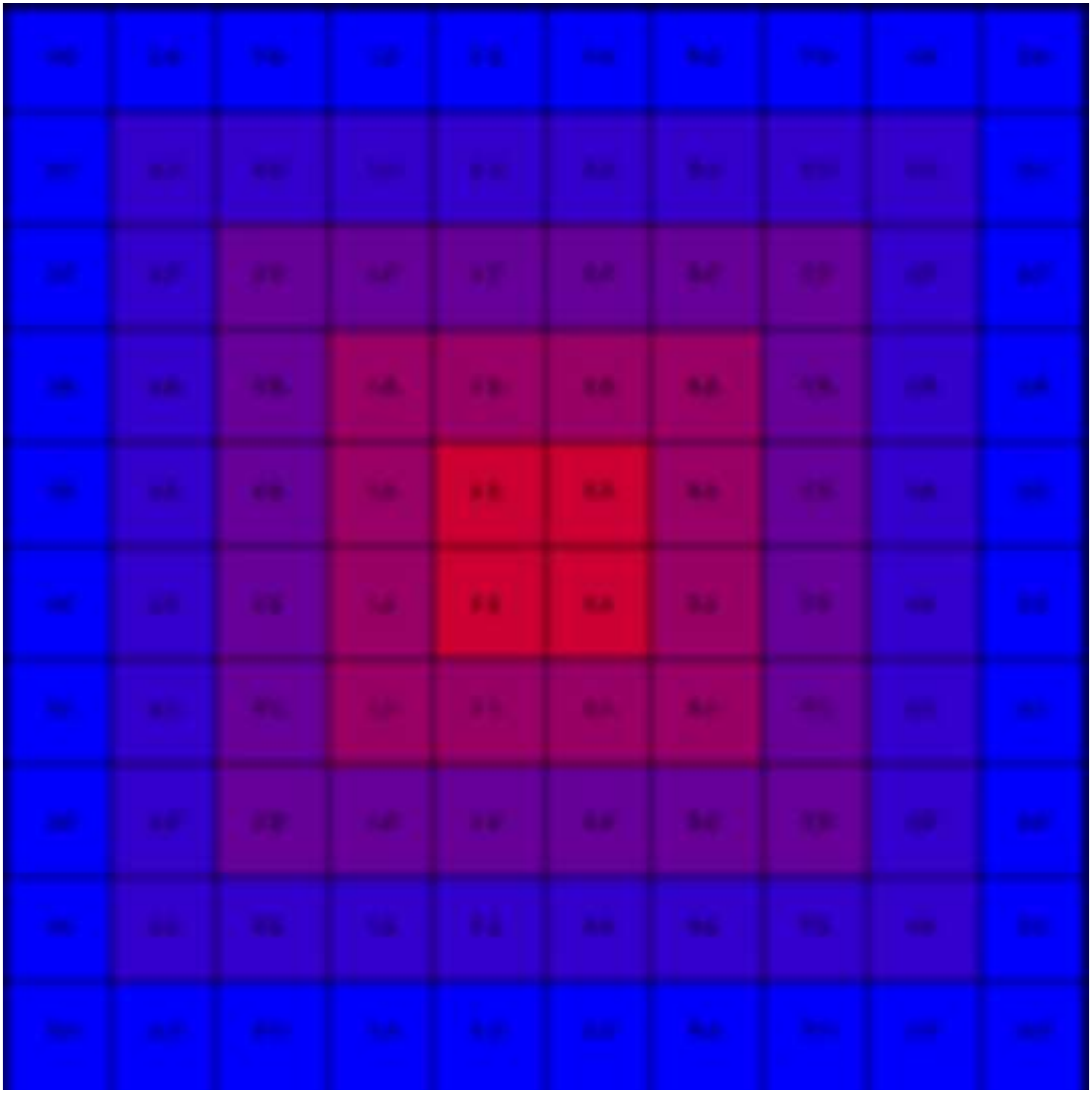

Click here to access/download;LaTeX - Figure (eps, ps, etc.);example-image.png

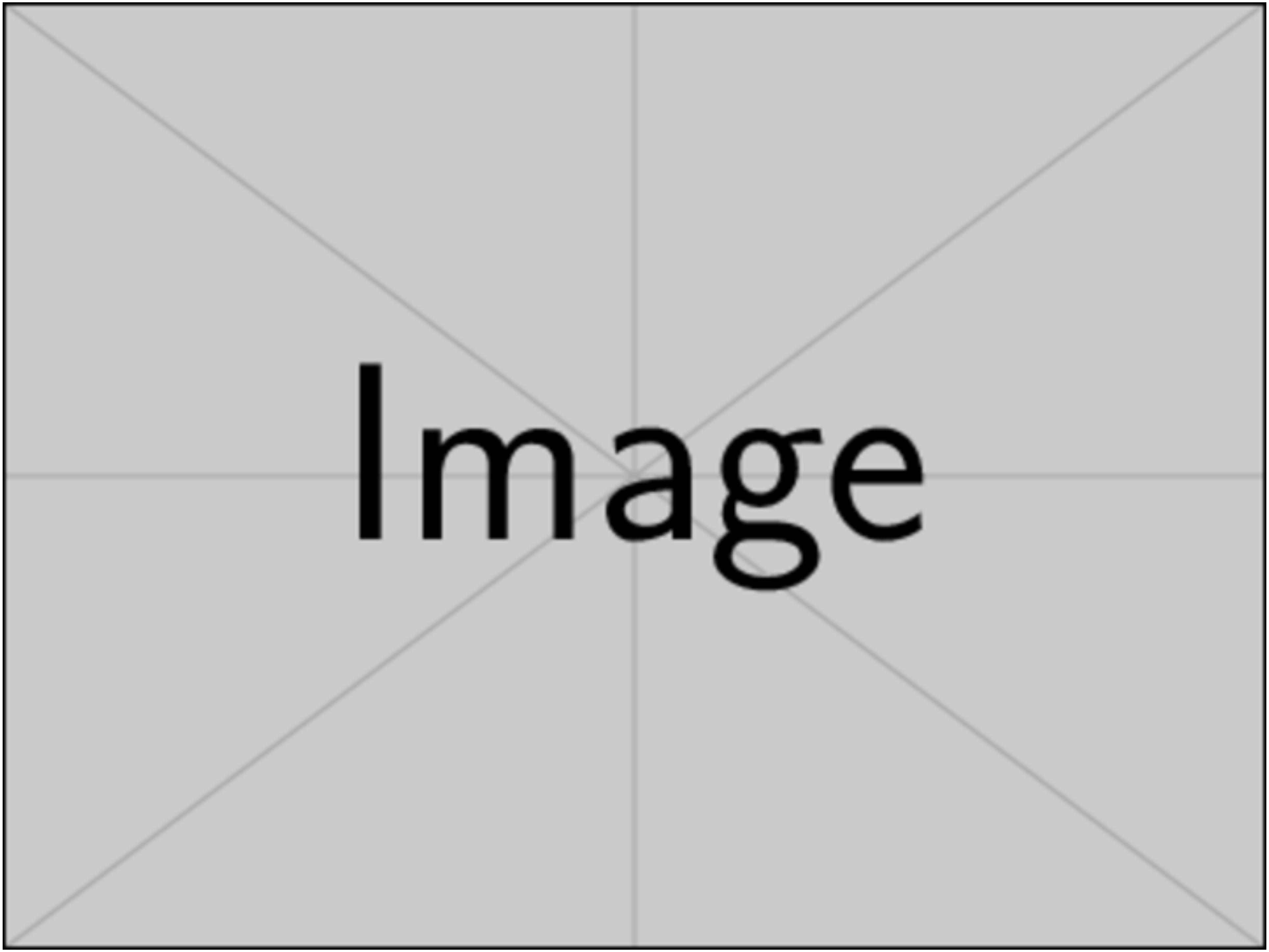

Reference PDF Click here to access/download;Manuscript;Reference PDF.pdf

OXFORD (GIGA)^n SCIENCE

GigaScience, 2023, 1–6

doi: xx.xxxx/xxxx
Manuscript in Preparation
Paper

PAPER

MetGENE: Gene-centric Metabolomics Information Retrieval Tool

Sumana Srinivasan[1,*], Mano R. Maurya[1,*], Srinivasan Ramachandran[1], Eoin Fahy[1] and Shankar Subramaniam[1,†]

[1]Department of Bioengineering, University of California, San Diego, La Jolla, CA 92093

[†]shsubramaniam@ucsd.edu
[*]Contributed equally.

Abstract

Background Biomedical research often involves contextual integration of multi-modal and multi-omic data in search of mechanisms for improved diagnosis, treatment and monitoring. Researchers need to access information from diverse sources comprising data in various and sometimes incomplete formats. The downstream processing of the data, to decipher mechanisms by reconstructing networks and developing quantitative models, warrants considerable effort. **Results** MetGENE is a knowledge-based, gene-centric data aggregator that hierarchically retrieves information about the gene(s), their related pathway(s), reaction(s), metabolite(s), and metabolomic studies from standard data repositories under one dashboard to enable ease of access through centralization of relevant information. Further, the information can be contextualized by filtering along species, anatomy (tissue) and condition (disease or phenotype). **Conclusions** MetGENE is an open source tool that aggregates metabolite information for a given gene(s) and presents them in different computable formats, e.g., JSON, for further integration with other omics studies. MetGENE is available at https://bdcw.org/MetGENE/index.php.

Key words: metabolomics workbench; gene-centric; data aggregator; web application

Introduction

Recent advances in high-throughput technologies have led to many high- resolution multiomic measurements available to bio-medical researchers. However, obtaining biological insights remains challenging since considerable effort is required to find and access data from diverse data sources, deal with various and sometimes incomplete data formats, and tease out the connections within those high-dimensional datasets. This has led to an initiative by the US National Institutes of Health (NIH) called the Common Fund Data Ecosystem (CFDE), which aims to provide a single portal that makes data findable, accessible, interoperable and re-usable across the data repositories maintained by Data Coordination Centers (DCCs). Some examples of DCCs, include the Metabolomics Workbench (MW), which is a national metabolomics data repository [1], Genotype-Tissue Expression (GTEx) Project, a comprehensive resource to study tissue-specific gene expression and regulation [2], and the Library of Integrated Network-Based Cellular Signatures (LINCS) with the goal of generating a large-scale and comprehensive catalogue of perturbation-response signatures by utilizing a diverse collection of perturbations across many model systems and assay types [3]. MW is a comprehensive resource hosting more than 2000 curated metabolomics studies and provides an integrated environment for data analysis and visualization through a suite of tools and interfaces to facilitate gaining biological insights.

A gene is a fundamental unit of query in the multi-omics data hierarchy. One of the goals of CFDE is to make every DCC support gene-centric querying within their repositories. Currently, MW supports a limited capability to perform gene-centric queries on the studies. MetGENE was designed to bridge this gap and enhance the capability by allowing a user to specify a gene or a set of genes as a search term and, in return, fetch the relevant information from sources like the Kyoto Encyclopedia of Genes and Genomes (KEGG) [4] and the MW. Given one or more genes, the MetGENE tool identifies associations between the gene(s) and the metabolites (biosynthesized/catabolized or transported by proteins coded by the

Compiled on: January 19, 2023.
Draft manuscript prepared by the author.

Key Points

- Knowledge-based data aggregator.
- Gene-centric query.
- Metabolomics Workbench studies.

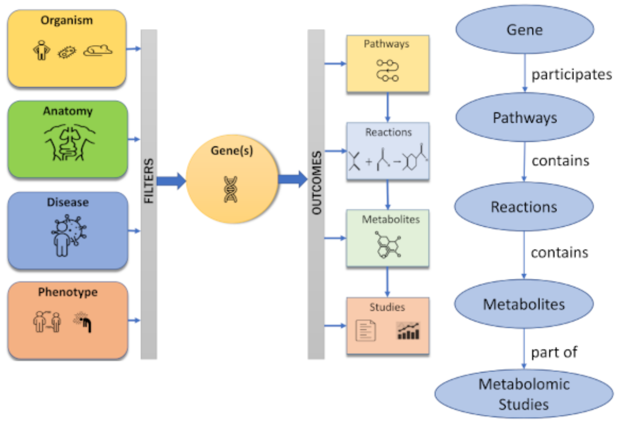

Figure 1. Schematic of MetGENE and the underlying knowledge graph. Gene(s) search is contextualized by organism name. The gene-associated pathways, reactions, metabolites and their corresponding metabolomic studies are reported as outcomes. Metabolites and Studies information can be filtered using anatomy (sample source), disease and phenotype.

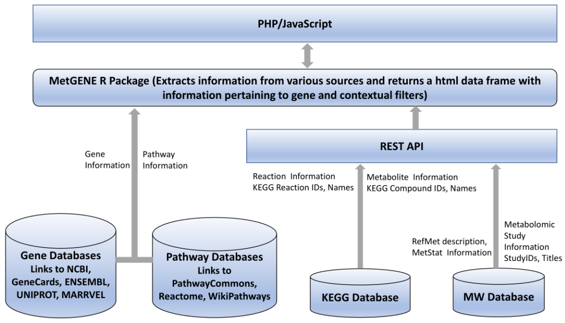

Figure 2. The Architecture of MetGENE comprises server-side PHP and JavaScript interacting with R scripts that use REST APIs to extract information from KEGG and MW databases. The gene and pathway information links are generated for specific repositories.

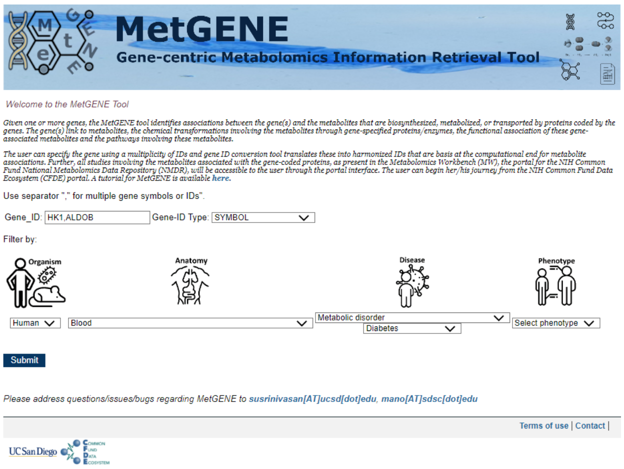

Figure 3. MetGENE Query page with organism, anatomy (sample source), disease and phenotype-specific filters.

genes), and the reactions and pathways involving these metabolites. For each metabolite, studies containing the metabolite are identified from the MW. The results are organized in the form of a gene landing page or a Dashboard with all the information presented in a user-friendly manner to enable further analyses.

Methods

MetGENE is a hierarchical, knowledge-based gene-centric information retrieval tool. Given a gene or a set of genes as a search term, MetGENE returns entities associated with the gene(s), namely pathways, reactions, metabolites and metabolomic studies in MW as shown in 1. MetGENE also contextualizes the search by allowing the users to specify filters based on organism name, anatomy or tissue name (broadly, sample source), disease and phenotype as a part of its query interface, as shown in Figure 1.

It is designed as a web-based application using PHP and JavaScript as the front-end and at the back-end, R scripts with wrapper functions to retrieve information from various data repositories such as KEGG [4] (for reaction and metabolite/compound IDs) and Metabolomics Workbench (for metabolite study IDs and RefMet names) as shown in Figure 2. RefMet names provide a standardized reference nomenclature for both discrete metabolite structures and metabolite species identified in metabolomic experiments. This is an essential prerequisite for the ability to compare and contrast metabolite data across different experiments and studies. MetGENE maintains session variables for species ID and organism name; ENTREZ gene ID and gene symbol; anatomy, disease and phenotype terms, and the previous values of these terms to enable server-side caching of pages and thus avoid unnecessary and time-consuming fetching of data across the network. The MetGENE back-end R functions are packaged into a library called metgene and will be available on GitHub for download. For programmatic ease of access, we provide REST APIs that output each table of the information displayed in JSON or text formats. The REST APIs are developed using Smart/OpenAPI format [5].

Results

In this section, we describe the user experience starting from the MetGENE Query Page and ending with MetGENE Studies Page containing metabolomic studies corresponding to the gene in the Metabolomics Workbench, incorporating various intermediate views of interest based on the knowledge graph described earlier.

MetGENE Query Interface

The user can input the gene information as a gene ID in any of the available formats. The format of the query page is as shown in 3. MetGENE supports SYMBOL, ENTREZ ID, RefSeq, UniProt, Ensembl and ALIAS (SYMBOL_OR_ALIAS) formats and converts IDs using an in-house Gene ID Conversion Tool (GICT). The GICT uses R Bioconductor packages org.Xy.eg.db (e.g., org.Hs.eg.db for human) and

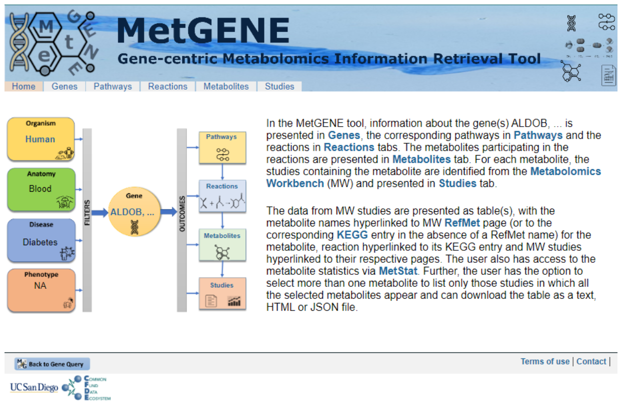

Figure 4. MetGENE landing page with context-sensitive display and access to Gene, Pathway, Reactions, Metabolites and Study information.

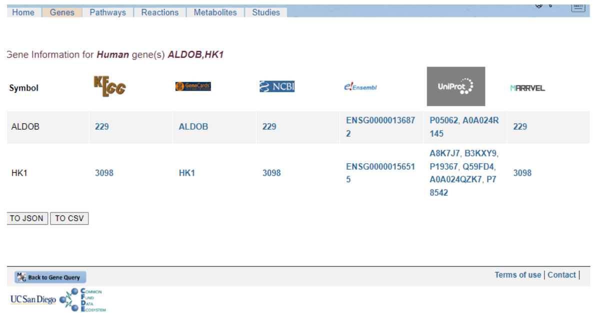

(a) Genes tab view.

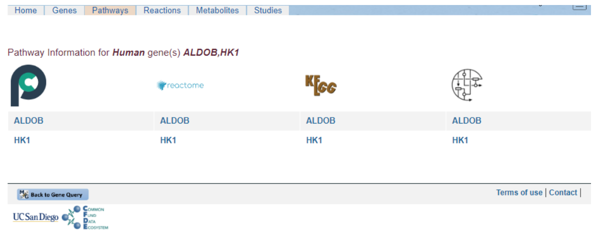

(b) Pathways tab view.

Figure 5. (a) MetGENE gene information page comprising Gene IDs in various formats corresponding to the searched gene(s) hyperlinked to various online resources. (b) MetGENE pathway information page comprising Gene IDs hyperlinked to various pathway resources.

NCBI gene_info table to convert the gene IDs. If the ID type for the input term is SYMBOL_OR_ALIAS, then the term is first searched in SYMBOL. If not found, then it is searched in ALIAS. The gene search input is validated on the client-side to allow only alphanumeric symbols. Invalid gene IDs are recognized, and appropriate error messages are displayed. MetGENE uses terms (e.g., Human, Mouse) for taxonomy filtering as per the NCBI taxonomy database (Coordinators, 2000). For filtering the information on metabolites and studies by anatomy/tissue (e.g., Liver, Blood), disease (e.g., Diabetes, Fatty liver disease) and phenotype (in-progress), terms from Metabolomics Workbench are used. Internally, in the MW database, both disease and phenotype are recorded under the metadata field, "disease". Hence, phenotype will be searched as a disease term internally. The JSON files for each of these categories are curated and updated regularly and used to generate a pull-down menu. For disease, a two-step selection menu with slim (or disease class) terms in the first level and fine-grained terms in the second level is used for ease of presenting the options to the user. The user inputs from this page (main landing page) are submitted as a form and a second page for MetGENE (as shown in Figure 4) is populated with the context-specific filtering terms. The second page comprises of tabs for the search term associated entities, "Genes", "Pathways", "Reactions", "Metabolites", and "Studies".

Gene and Pathway Information Pages

The gene information page shown in Figure 5a presents gene IDs in different formats hyperlinked to the corresponding web pages pointing to repositories such as KEGG [4], GeneCards [6], NCBI [7], Ensembl [8], UniProt [9] and Marrvel [10]. The URLs to these repositories for the specific genes are constructed based on their base URLs and the respective supported gene ID types. This information is obtained from the REST API of the GICT and converted from JSON to a HTML table format for display purposes.

The pathway information page (Figure 5b) displays gene symbols hyperlinked with species and gene ID or symbol information as appropriate to various well-maintained pathway databases such as Pathway Commons [11], Reactome [12], KEGG [4] and Wikipathways [13]. MetGENE provides context-specific ease of access to these online resources.

Reaction and Metabolite Information Pages

The KEGG database provides the KEGG REST API to access information from the KEGG database. Given the three-letter KEGG organism code and the ENTREZ gene ID of the gene, the R KEGGREST API provides a way to access all the information such as pathway IDs, reaction IDs and compound (metabolites) IDs as a data frame object. The reaction IDs and their names for the given gene or set of genes are displayed in a tabular format as shown in Figure 6a, with the reaction IDs hyperlinked to the corresponding KEGG reaction information page. The Metabolomics Workbench provides REST APIs to access the RefMet names corresponding to the KEGG compound IDs.

In the metabolites information page, as shown in Figure 6b, a unique list of metabolites across all reactions corresponding to a given species, along with their respective RefMet names, KEGG reaction IDs of all the reactions the metabolite participates in, are displayed. Further, the MW MetStat link provides access to the statistics about the metabolite measured across various studies in the MW database, filtered by anatomy (sample source), disease and phenotype names. This tool generates a report for any given metabolite in MW comprising all the unique studies containing that metabolite and their median value of the relative standard deviation (RSD) across all those studies. The KEGG compound IDs that do not have RefMet names in the MW database display only the KEGG compound name and reaction IDs. MetGENE allows users to download the tables directly from the displayed page in JSON or CSV formats for further analysis. MW also provides REST APIs to access all the study IDs, titles, and RefMet names for a given KEGG compound ID in JSON, text and HTML formats.

MetGENE Metabolomics Study Information Page

In the Studies page (as shown in Figure 7a), a tabular view of the unique list of metabolites for the queried gene(s), their RefMet names hyperlinked to their corresponding description page in MW, and a comma separated list of study IDs in which the metabolite participates (with each study ID linked to its corresponding study description page in MW) is presented. Further, a helpful text hover feature displaying the study title corresponding to a particular study ID is also provided to the user. MetGENE also allows the user to select metabolites of interest and combine their studies for download and further analysis, as shown in Figure 7b.

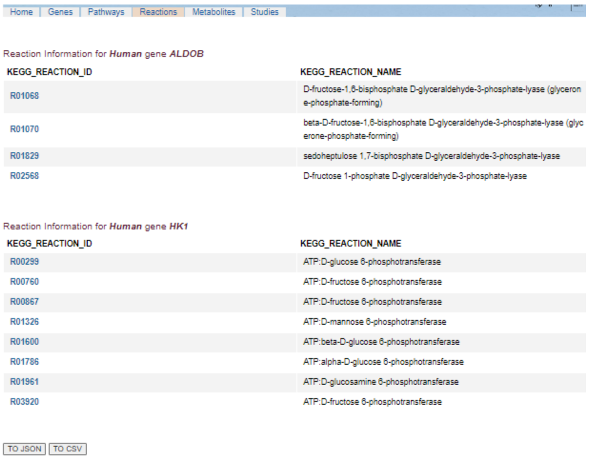

(a) Reactions tab view.

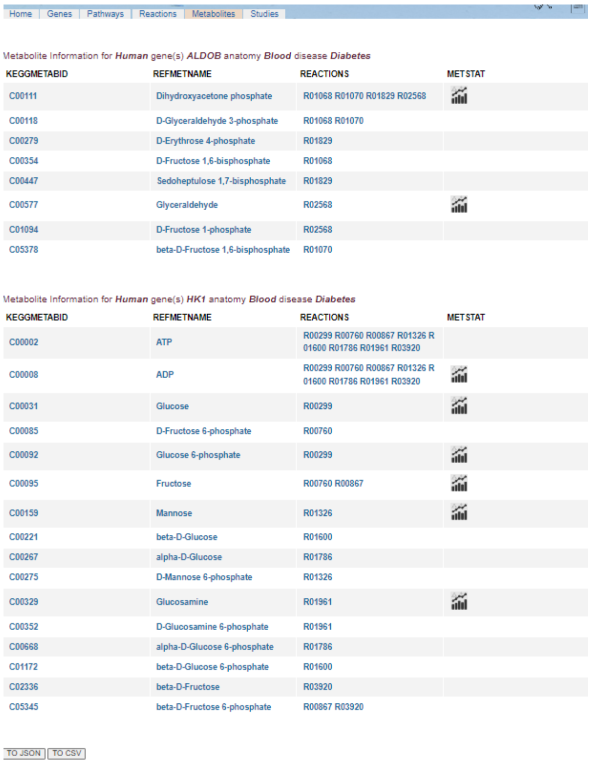

(b) Metabolites tab view.

Figure 6. (a) MetGENE reaction information page comprising KEGG Reaction IDs hyperlinked to KEGG reaction information page and reaction descriptions in a tabular format. (b) MetGENE metabolite information page comprises KEGG compound IDs, MW RefMet names, reaction IDs in which a metabolite participates in, hyperlinked to the KEGG reaction information page and MetStat link for the metabolite in a tabular format.

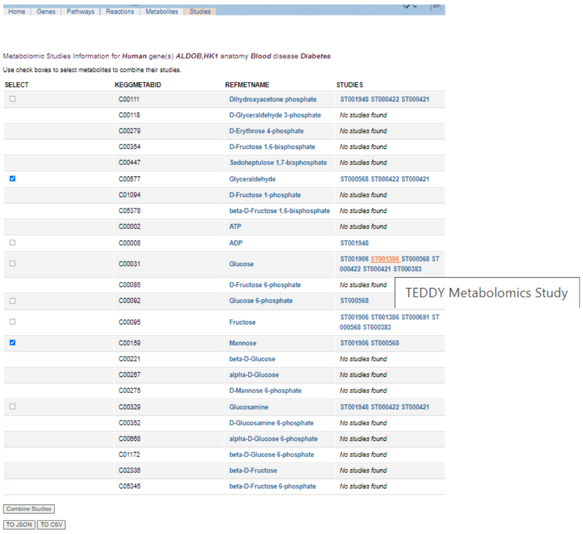

(a) Studies tab view.

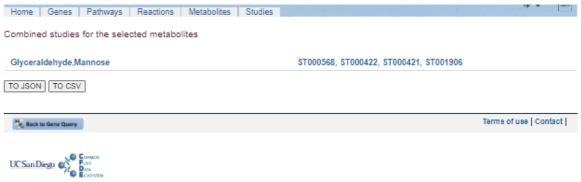

(b) Combined studies tab view.

Figure 7. (a) MetGENE metabolomics studies information page comprises KEGG compound IDs, RefMet names, MW study IDs corresponding to a metabolite in a tabular format. (b) MetGENE allows users to combine studies for a selected set of metabolites.

Case Study: Exploring gene PNPLA3 using MetGENE

Here we demonstrate a use case that shows the utility of MetGENE as a one-stop tool to obtain all metabolomic information associated with a gene(s) in a specific disease condition. The protein Adiponutrin, encoded by the gene PNPLA3, is a multi-functional enzyme that belongs to the IPLA2/lipase family, which has both triacylglycerol lipase and acylglycerol O-acyltransferase activities. PNPLA3 is predominantly expressed in adipocytes and liver cells. It regulates the development of adipocytes and the metabolism of fats (lipogenesis and lipolysis). Diseases associated with PNPLA3 mutations include Fatty Liver Disease and Non-Alcoholic Steatohepatitis (NASH) [14],[15], [16] and [17].

To obtain information about this gene and associated entities like enzymes, reactions, pathways and existing metabolomic studies in MW in a given context (fatty liver disease in humans), a user needs to identify very specific key terms, perform a search on the internet, sift through the results to identify literature about various metabolomic studies and download the studies to perform downstream analyses. These steps are sometimes time-consuming and misleading depending on the search terms' specificity. However, with MetGENE, the user can specify gene ID in any of the popular formats, apply the filters pertaining to organism, anatomy, disease and phenotype and directly obtain gene, pathways, reaction, metabolite and studies information consolidated from various data resources, at one go. Supplementary Figure A1 shows the Gene tab for the PNLPA3 Gene. Links to various online resources (GeneCards, KEGG, NCBI, Uniprot and Marrvel) with genomic, transcriptomic, proteomic, clinical and functional information (via GeneCards link), module and pathway information (via KEGG), transcript and region information (via NCBI link), sequence, variant, gene expression information (via Ensembl link), protein sequence and functional information (via Uniprot link) and gene variants associated with

PNLPA3 (via Marrvel link) are provided in the gene information table. Supplementary Figure A2 represents the Pathway tab for the PNLPA3 gene with links to interactions with and pathways that involve PNPLA3 (via Pathway Commons), protein and reaction location information (via Reactome), pathway definitions (via KEGG), and pathway related collaborative information (via Wikipathways). Supplementary Figure A3 represents the Reactions and Metabolites tabs for PNPLA3. It delineates two hydrolysis reactions where diacylglycerol (DAG) is hydrolyzed to 2-monoacylglycerol (2-MAG), and triacylglycerol (TAG) is hydrolyzed to DAG by PNPLA3. Three generic compounds (TAG, DAG, and Fatty acid) participate in the reactions. The Metabolites tab for PNPLA3 lists all the metabolites (with substitutions for TAG and DAG) along with corresponding RefMet names or KEGG metabolite names (in the absence of RefMet names) along with a MetStat link that points to metabolite statistics information such as a histogram of the RSD (Relative Standard Deviation) metabolite data, ANOVA results for the metabolite with a cut- off p-value in MW via MetStat, with the anatomy and disease filters applied. Supplementary Figure A4 depicts the Studies tab in MetGENE for the gene PNPLA3. Each metabolite would display the corresponding Study IDs if measured/listed in any studies deposited in MW, hyperlinked to the study description. A hover text displays the study title. The tool provides the user with the ability to combine studies for metabolites of interest to a consolidated view as shown in Supplementary Figure A4. All of MetGENE tables can be downloaded in formats such as JSON and CSV directly from the respective pages in the browser or via the REST API. The REST API supports JSON and text formats. They are deposited in the Smart API repository along with the accompanying documentation.

Discussion

Given one or more genes, the MetGENE tool identifies associations between the gene(s) and the metabolites that are biosynthesized, catabolized, or transported by proteins coded by the genes. It is a knowledge-based data aggregator accessing and integrating data from resources such as the KEGG and Metabolomics Workbench to name a few. The gene(s) link to metabolites, the chemical transformations involving the metabolites through gene-specified proteins/enzymes, the functional association of these gene-associated metabolites and the pathways involving these metabolites with context-based filtering based on anatomy (sample source), disease and phenotype. The user can specify the gene using a multiplicity of IDs and the gene ID conversion tool translates these into harmonized IDs that are basis for metabolite associations. Further, all studies involving the metabolites associated with the gene-coded proteins, as present in the Metabolomics Workbench (MW) will be accessible to the user, as a stand-alone tool or via the portal interface for the NIH Common Fund National Metabolomics Data Repository (NMDR). The user can begin their journey either from the main web page for MetGENE (see Availability) or from the NIH Common Fund Data Ecosystem (CFDE) portal (https://app.nih-cfde.org/; the steps are: Data Browser → Vocabulary → Gene).

Potential implications

Features from MetGENE will contribute to integration of other omics data with metabolomics data, with genes serving as the bridging molecules or nodes. For example, tools such as MetGENE will assist a researcher to interpret results of multi-omics data integration holistically where they can consider both gene-related data and metabolomics data in a metabolic pathway.

Availability of Supporting Code and Requirements

MetGENE is an open source collaborative initiative available at GitHub. The main website of MetGENE and the Smart APIs are available as shown below.

- Project Name : MetGENE
- Project Home Page: https://bdcw.org/MetGENE/index.php
- Operating System: Platform independent
- Programming languages: R and PHP
- Smart APIs: https://smart-api.info/registry/?q=MetGENE
- Source code: https://github.com/metabolomicsworkbench/MetGENE

Additional Files

- Supplementary Figure A1: The gene information tab for PNPLA3 in MetGENE has links to various online resources that provide different type of information pertaining to the gene including sequence, gene expression, protein structure, functional and disease information.
- Supplementary Figure A2: The pathway information tab for PNPLA3 in MetGENE has links to various online resources that provide different type of information pertaining to the pathways in which PNPLA3 participates.
- Supplementary Figure A3: The reaction and metabolite information tabs for PNPLA3 in MetGENE has links to reactions controlled by PNLPA3 with Reaction IDs linked to KeggKEGG reaction definition page. RefMet provides the metabolite details and MetStat link provides the context specific metabolite measurement statistics.
- Supplementary Figure A4: The studies information tab for PNPLA3 in MetGENE has links to studies in MW pertaining to metabolites listed. Studies can be combined for selected metabolites for further analysis.

Declarations

List of abbreviations

- CFDE - Common Fund Data Ecosystem
- DCC - Data Coordination Centers
- GTEx - Gene Tissue Expression
- LINCS - Library of Integrated Network-Based Cellular Signatures
- GICT - Gene ID Conversion tool
- MW - Metabolomics Workbench
- NMDR - National Metabolomics Data Repository

Ethical Approval

Not Applicable.

Consent for publication

Not Applicable.

Competing Interests

Not Applicable.

Funding

This work has been supported by the National Institutes of Health Grants (Metabolomics Workbench, U2C-DK119886 and Common Fund Data Ecosystem (CFDE) OT2-OD030544).

Author's Contributions

This work was conceptualised by Sh.S., Su.S., M.R.M. and S.R. The funding acquisition was by Sh.S. The methodology, software development and visualization for MetGENE were developed by Su.S. and M.R.M. Validation and testing was performed by Su.S., M.R.M., S.R. and Sh.S. The disease ontology terms and structuring was provided by S.R. The Metabolomics Workbench REST APIs that are used in this work were developed by E.F. The original draft for the paper was prepared by Su.S and reviewing and editing was done by Sh.S, M.R.M. and S.R. The project administration was done by Sh.S and M.R.M.

Acknowledgements

We thank the Common Fund Data Ecosystem Gene Working Group for useful discussions.

References

1. Sud M, Fahy E, Cotter D, Azam K, Vadivelu I, Burant C, et al. Metabolomics Workbench: An international repository for metabolomics data, metadata and metabolite standards, protocols, tutorials and training, and analysis tools. Nucleic acids research 2016;44:D463–70. http://www.metabolomicsworkbench.org.
2. GTExConsortium. Human genomics. The Genotype-Tissue Expression (GTEx) pilot analysis: multitissue gene regulation in humans. Science 2015;348(6235):648–660. https://gtexportal.org/home/.
3. V S, J T, A K, D V, D C, M FN, et al. LINCS Data Portal 2.0: next generation access point for perturbation-response signatures. Nucleic Acids Research 2020;48:D431–D439.
4. Kanehisa M, Goto S. KEGG: kyoto encyclopedia of genes and genomes. Nucleic Acids Research 2000;28(1):27–30. https://www.genome.jp/kegg/kegg1.html.
5. Schloerke B, Allen J. plumber: An API Generator for R; 2022, https://www.rplumber.io, https://github.com/rstudio/plumber.
6. Safran M, Rosen N, Twik M, BarShir R, Stein TI, Dahary D, et al. In: The GeneCards Suite Singapore: Springer Singapore; 2021. p. 27–56. https://doi.org/10.1007/978-981-16-5812-9_2.
7. S F. The NCBI Taxonomy database. Nucleic acids research. Nucleic Acids Research 2012;40:D136–D143. https://doi.org/10.1093/nar/gkr1178.
8. Cunningham F, Allen JE, Allen J, Alvarez-Jarreta J, Amode MR, Armean IM, et al. Ensembl 2022. Nucleic Acids Research 2022;50(1):D988–D995. https://doi.org/10.1093/nar/gkab1049.
9. Consortium TU. UniProt: the universal protein knowledgebase in 2021. Nucleic Acids Research 2021;49(D1):D480–D489. https://doi.org/10.1093/nar/gkaa1100.
10. Wang J, Al-Ouran, R, Hu, Y, Kim, et al. MARRVEL: Integration of Human and Model Organism Genetic Resources to Facilitate Functional Annotation of the Human Genome. American journal of human genetics 2021;100(6):843–853. https://doi.org/10.1016/j.ajhg.2017.04.010.
11. Cerami EG, Gross BE, Demir E, Rodchenkov I, Babur O, Anwar N, et al. Pathway Commons, a web resource for biological pathway data. Nucleic Acids Research 2010;39:D685–D690. https://www.pathwaycommons.org/.
12. Gillespie M, Jassal B, Stephan R, Milacic M, Rothfels K, Senff-Ribeiro A, et al. The reactome pathway knowledgebase 2022. Nucleic Acids Research 2021;50(D1):D687–D692. https://reactome.org/.
13. M M, A A, A R, A W, DN S, K H, et al. WikiPathways: connecting communities. Nucleic Acids Research 2021;49:D613–D621. https://doi.org/10.1093/nar/gkaa1024.
14. Cohen J, Horton J, HH H. Human fatty liver disease: old questions and new insights. Science 2011;332(6037):1519–23.
15. Gorden D, Myers D, Ivanova P, Fahy E, Maurya M, Gupta S, et al. Human fatty liver disease: old questions and new insights. Science 2011;332(6037):1519–23.
16. P P, S R. The role of PNPLA3 in health and disease. Biochem Biophys Acta Mol Cell Biol Lipids 2019;1864(6):900–906.
17. XC D. PNPLA3—A Potential Therapeutic Target for Personalized Treatment of Chronic Liver Disease. Front Med 2019;6(304).

Figure A1

Click here to access/download
Supplementary Material
FigureA1_Supplementary.png

Figure A2

Click here to access/download
Supplementary Material
FigureA2_Supplementary.png

Figure A3

Click here to access/download
Supplementary Material
FigureA3_Supplementary.png

Figure A4

Click here to access/download
Supplementary Material
FigureA4_Supplementary.png

Cover letter
Click here to access/download;Personal Cover;MetGENE_EditorLetter_GigaSci-Final.pdf

UNIVERSITY OF CALIFORNIA, SAN DIEGO UCSD

BERKELEY • DAVIS • IRVINE • LOS ANGELES • MERCED • RIVERSIDE • SAN DIEGO • SAN FRANCISCO SANTA BARBARA • SANTA CRUZ

Shankar Subramaniam
Joan and Irwin Jacobs Professor of Bioengineering & Systems Biology
Department of Bioengineering
University of California at San Diego
9500 Gilman Drive
La Jolla, CA 92093-0427
SHANKAR@UCSD.EDU
Tel: (858) 822-0986

Dr. Scott Edmunds
Editor-in-Chief, GigaScience

January 19, 2023

Sub: Submission of a manuscript to GigaScience

Dear Editor,

We would like to submit a manuscript entitled "*MetGENE: Gene-centric Metabolomics Information Retrieval Tool*" for consideration in the esteemed journal *GigaScience*.

Metabolomics is emerging as an important area of biomedical research since metabolites offer the capability to characterize cells and tissues and are end points of physiological phenotypes. Modern biomedical research heavily relies on contextual integration of multi-modal and multi-omic data in search of mechanisms for improved diagnosis, treatment and monitoring. Researchers need to access information from diverse sources comprising data in various and sometimes incomplete formats. The downstream processing of the data, to decipher mechanisms by reconstructing networks and developing quantitative models, warrants considerable effort. In the context of metabolomics data, association of genes with metabolites serves as a natural first step in the integration of metabolomics data with other omics data and knowledge. To address this need we have developed a web-based tool called MetGENE and we describe the resource in this manuscript.

MetGENE is a knowledge-based, gene-centric data aggregator that hierarchically retrieves information about the gene(s), their related pathway(s), reaction(s), metabolite(s), and metabolomic studies from the National Metabolomics Data Repository (NMDR) under one dashboard to enable ease of access through centralization of relevant information. Further, the information can be contextualized by filtering along species, anatomy (tissue) and condition (disease or phenotype).

In the manuscript, we provide exemplar use-cases that will inform the end-user of the potential applications supported by MetGENE. MetGENE is available as a web-based tool (https://bdcw.org/MetGENE/index.php) and as a SmartAPI (more information provided at https://github.com/metabolomicsworkbench/MetGENE), making its integration into other tools quite easy. It is our view that MetGENE will encourage further development of gene-metabolite data integration tools.

Further, we declare the followings:

An explanation of any issues relating to journal policies: None

A declaration of any potential competing interests: No competing interests

Confirmation that all authors have approved the manuscript for submission: Yes

Confirmation that the content of the manuscript has not been published, or submitted for publication elsewhere: Yes

I hope that the manuscript will be suitable for consideration in the journal GigaScience.

We thank you for your consideration.

Sincerely,

Shankar Subramaniam, Ph.D.
