# Supplementary material for: MetGENE: gene-centric metabolomics information retrieval tool
Source: Gigascience. 2023 Nov 20;12:giad089. doi: 10.1093/gigascience/giad089 (PMC10659118; doi:10.1093/gigascience/giad089)
Supplement: giad089_GIGA-D-23-00021_Revision_1 [file giad089_giga-d-23-00021_revision_1.pdf]

GigaScience

MetGENE: Gene-centric Metabolomics Information Retrieval Tool

--Manuscript Draft--

**Manuscript Number:**	GIGA-D-23-00021R1
**Full Title:**	MetGENE: Gene-centric Metabolomics Information Retrieval Tool
**Article Type:**	Research
**Funding Information:**	National Institutes of Health (U2C-DK119886) — Dr Shankar Subramaniam<br>National Institutes of Health (OT2-OD030544) — Dr Shankar Subramaniam
**Abstract:**	Background Biomedical research often involves contextual integration of multi-modal and multi-omic data in search of mechanisms for improved diagnosis, treatment and monitoring. Researchers need to access information from diverse sources, comprising data in various and sometimes incongruent formats. The downstream processing of the data to decipher mechanisms by reconstructing networks and developing quantitative models warrants considerable effort. Results MetGENE is a knowledge-based, gene-centric data aggregator that hierarchically retrieves information about the gene(s), their related pathway(s), reaction(s), metabolite(s), and metabolomic studies from standard data repositories under one dashboard to enable ease of access through centralization of relevant information. We note that MetGENE focuses only on those genes that encode for proteins directly associated with metabolites. All other gene-metabolite associations are beyond the current scope of MetGENE. Further, the information can be contextualized by filtering by species, anatomy (tissue) and condition (disease or phenotype). Conclusions MetGENE is an open-source tool that aggregates metabolite information for a given gene(s) and presents them in different computable formats, e.g., JSON, for further integration with other omics studies. MetGENE is available at https://bdcw.org/MetGENE/index.php
**Corresponding Author:**	Shankar Subramaniam, Ph.D.<br>University of California San Diego<br>La Jolla, CA UNITED STATES
**Corresponding Author Secondary Information:**	
**Corresponding Author's Institution:**	University of California San Diego
**Corresponding Author's Secondary Institution:**	
**First Author:**	Shankar Subramaniam, Ph.D.
**First Author Secondary Information:**	
**Order of Authors:**	Shankar Subramaniam, Ph.D.<br>Sumana Srinivasan<br>Mano Ram Maurya<br>Srinivasan Ramachandran<br>Eoin Fahy
**Order of Authors Secondary Information:**	
**Response to Reviewers:**	Main comments<br><br>Reviewer 1:<br><br>1. Overall the knowledge-base is well designed, with a description of the data retrieval mechanism on the query page. However, the term "associated" metabolites to a gene is rather open-ended. It seems that the association is via the encoded protein to a gene, and its known enzymatic activity. This then links the gene to a metabolite, and

Powered by Editorial Manager® and ProduXion Manager® from Aries Systems Corporation

known pathways where the enzyme is active. There are many other ways that a gene may have an "association" with a small molecule metabolite however - such as via regulation of the gene or its protein product with small molecules metabolites, literature-based connections, gene expression-metabolite associations etc. It may help the user to know what associations are not a part of the analysis, so the user knows what to expect in terms of how information is retrieved from any given query.

Response: We thank the reviewer for this important comment. We have added the following line in the Abstract section, “MetGENE focuses only on those genes that encode for proteins directly associated with metabolites. All other gene-metabolite associations are beyond the current scope of MetGENE.” We have also elaborated on this point in the Introduction section, “There are many other ways a gene may have an "association" with a small molecule metabolite, such as via gene regulation (e.g., TF-target relationship) or its protein product, gene expression-metabolite association, protein-protein-metabolite association, etc. However, in MetGENE, we only focus on those genes that encode for proteins directly associated with metabolites; these include metabolic enzymes, transporters and receptors.”. We have included the number of associations supported by MetGENE in Figure1C.

2. Related to the point above, a key linkage of a gene to a metabolite is the encoded protein that has catalytic activity. Although it is possible to get to the information on the protein and its enzymatic activity via the Uniprot link via a couple of clicks by the user, it seems that the Uniprot link could be displayed more prominently on the initial results page after the query is entered. This may be a more efficient way for users to get information on the protein, as a key linkage point to the metabolite/pathway information.

Response: We thank the reviewer for this suggestion, and we agree that providing Uniprot link in the main screen may be of help to the user. However, for a given gene, there are several Uniprot IDs and displaying all this information in the main screen would lead to visual cluttering ad hence we decided to display this in the Genes tab along with other useful set of links such as KEGG, Ensembl, etc. which may provide more information to the user.

3. It is understood that the "phenotype" query is not available yet. However, it's unclear what type of queries will be available here. Could the authors provide some information on the options for phenotypes that they plan to make available?

Response: In the current version on MetGENE, we decided to completely do away with the Phenotype menu in the query screen. We have combined both the Disease as well as the Phenotype into a consolidated two step cascading menu list. We have updated Figure 3 in the manuscript to reflect this change. The Metabolomics Workbench does not recognize “Phenotype” as a separate search term and hence we decided to consolidate both disease and phenotype into a single list. However, in the future, if MW decides to support querying based on the Phenotype terms from databases such as Human Phenotype Ontology, we will support it.

4. They authors should be more clear on exactly how many organisms can be queried - at this point it looks like it is a relatively limited number (human, mouse, rat). Are there plans to include any more organisms - such as pathogenic bacteria or other organisms related to human health that might be of interest?

Response: We agree with the reviewer and our apologies for not making it clear in our manuscript. We will include microorganisms relevant to human health and disease in a phased manner. We plan to include E. coli (K12), C. elegans, D. melanogaster, and A. gambiae soon and have mentioned this in the Result sections of the manuscript.

5. In test runs it might be nice to show the number of returned results under "Metabolites", "Reactions", "Pathways" in the first window. Some may be empty and it seems inefficient for the user to have to click through each category only to find out there are not any results?

Response: We thank the reviewer for an excellent suggestion. Since the time taken to compute the number of results can be high in the case of multiple genes or genes who

have large number of metabolites associated with them, adding this feature to the main landing page would slow down the loading of the page which is undesirable. We have created a “Summary” tab that displays the number of pathways, reactions, metabolites, and the studies both as a downloadable table as well as a pie chart graphic for better visualization (Figure 4). In addition, we have also added a feature where the tool warns the user about empty results if the gene does not encode a protein directly related to metabolites.

6. The "clear query" button to start a new query is a bit small - this could be more obvious to the user to clear the form and start again.

Response: We have increased the size and the graphic on the button to clear the query to address this issue.

Reviewer 2:

1. There are many methods/databases/tools linking genes to pathways, reactions, and metabolites. For example, MetaCyc or HumanCyc provides web and local interfaces, where the search can start from genes, proteins, pathways, or metabolites. Related information can also be easily fetched (e.g., from genes to related reactions or metabolites). Those databases tend to be curated, which increases accuracy but can probably miss possible connections. This manuscript provides database searching that is not curated which can possibly provide more connections. However, such comparisons are not stated in the manuscript. In addition, many of the databases (KEGG, GeneCards) the authors used and mentioned have blocks of genes, pathways, and metabolites. The authors need to explain the significance and improvement of MetGENE upon other databases or curated tools. Is MetGENE going to provide more complete results than any of them? How do MetGENE results tradeoff between coverage and accuracy compared with others?

Response: MetGENE uses a knowledge graph to relate metabolic genes to the metabolites and then downstream to metabolomic studies. The main value proposition of MetGENE is to get studies from MW by providing a gene centric front end. MetGENE is not a database and does not compete with other databases that provides Gene Pathways Reactions Metabolite connections but merely provides a context by becoming a one stop place for accessing other databases in context. Hence, we are not in a position to provide trade-off result comparing to other databases.

2. It seems that the interface with the metabolic workbench is one of the major innovations of this manuscript. The tool and visualization provided for this functionality are however limited. It's mainly through the block of metabolites (METSTAT) and studies, which indeed seem to provide interesting information. Can the author show how MetGENE helps improve users' understanding or efficiency through such integration with a specific biological example? How does integrating results of multiple workbench studies show something not directly visible in one? Some additional functionality in this part can be helpful to the users (e.g., a table of quantification and corresponding metabolites approaches of the study through API OR a summary information table for the study included in the Study page).

Response: At the moment, we are not providing any more extra insights by combining studies from MW. However, in the context of quantification of metabolites, METSTAT already provides summary (such as distribution of values of metabolites) based on information from multiple studies. Study summarization with respect to different types of metadata can be done (e.g., type of chromatography, MS/NMR etc). However, integration of information based on metadata (e.g., 6 studies were GC-MS, 4 Studies were LC-MS, etc.) may not be that useful. If we come across a use case that requires integration of multiple studies, we will incorporate it into MetGENE in the future.

3. The presentation of this paper has been mainly qualitative. More quantitative evaluation and presentations can help. For example, what's the size of the search space of genes for MetGENE to have any results? How many reactions, metabolites, pathways, and studies are connected?
Response: We thank the reviewer for the suggestion. Now, we support 1069 metabolic genes. We have represented statistics of the associations in Figure 1C.

Powered by Editorial Manager® and ProduXion Manager® from Aries Systems Corporation

	4. Only allowing starting from genes seems limited. The search should also be able to start with metabolites or reactions.<br>Response: We agree with the suggestion but expanding it to start from Reaction or Pathway is out of the current scope of MetGENE. Also, it would be hard for users to specify the Reaction/Pathway name or description as a starting point to query. We also note that the Metabolomics Workbench (https://www.metabolomicsworkbench.org/databases/refmet/refmet.php) already enables connecting metabolites to genes, reactions, and pathways.<br><br>5. Even though explained in the method, it's not clear to me what kind of connections between genes, pathways, reactions, and metabolites the authors used for the results. A supplementary figure showing the logical process of searching starting from one gene can be helpful. Particularly, the user can present a network starting from one specific gene (e.g., PNPLA3) and present how different parts (e.g., metabolites, pathways) are connected in the result.<br>Response: As per the suggestion of the reviewer, we have added an additional figure in the Supplementary Material (Figure A5) that depicts the information flow from query to the results for the use case of the gene PNPLA3.<br><br><br>Minor comments:<br><br>6. The pathway result is puzzling as it seems to be links connected to different databases searching for information related to the genes. When I click those links, the information is not solely on pathways. Supplying all the information in a table rather than links can be much easier for the user. The WikiPathways link seems also not working. Similarly, the author can add more information for the tables of reactions (showing the reaction equation itself).<br>Response: The reason MetGENE is designed to display a list of Pathway database links as opposed to the pathways themselves is to enable the user to have access to different types of information each of these databases provide. One could potentially scrape this information and display consolidated information but to decide what information is useful to display or not would be difficult to assess. As per the reviewer's suggestion, we have included the reaction equation as part of the Reactions table (Figure 6a). We have also updated our REST and Smart APIs to reflect this.<br><br>7. It seems white space after the comma when searching for two genes will cause problems.<br>Response: We have fixed this issue in the current version of the tool. We thank the reviewer for pointing it out.<br><br>8. There are many technical details in the result which can be put into methods or supplements. For example, the sentence "Given the three-letter KEGG organism code and the ENTREZ gene ID of the gene, the R KEGGREST API provides a way to access all the information such as pathway IDs, reaction IDs and compound (metabolites) IDs as a data frame objects" can be simplified without mention code length, specific ID, specific API methods, etc. The "MetGENE Query Interface" section of the result also seems too detailed and technical. The result part can be rewritten and reduced to improve accessibility to general readers.<br><br>Response: We appreciate the reviewer's feedback regarding simplifying the writing. We have simplified the writing in this revised version of the manuscript (Methods section).
**Additional Information:**	
**Question**	**Response**
Are you submitting this manuscript to a special series or article collection?	No
**Experimental design and statistics**	Yes

Powered by Editorial Manager® and ProduXion Manager® from Aries Systems Corporation

Full details of the experimental design and statistical methods used should be given in the Methods section, as detailed in our Minimum Standards Reporting Checklist. Information essential to interpreting the data presented should be made available in the figure legends.<br><br>Have you included all the information requested in your manuscript?	
**Resources**<br><br>A description of all resources used, including antibodies, cell lines, animals and software tools, with enough information to allow them to be uniquely identified, should be included in the Methods section. Authors are strongly encouraged to cite Research Resource Identifiers (RRIDs) for antibodies, model organisms and tools, where possible.<br><br>Have you included the information requested as detailed in our Minimum Standards Reporting Checklist?	Yes
**Availability of data and materials**<br><br>All datasets and code on which the conclusions of the paper rely must be either included in your submission or deposited in publicly available repositories (where available and ethically appropriate), referencing such data using a unique identifier in the references and in the "Availability of Data and Materials" section of your manuscript.<br><br>Have you have met the above requirement as detailed in our Minimum Standards Reporting Checklist?	Yes

Manuscript
Click here to access/download;Manuscript;FinalManuscript.pdf

OXFORD
(GIGA)n SCIENCE

GigaScience, 2023, 1–7

doi: xx.xxxx/xxxx
Manuscript in Preparation
Paper

PAPER

MetGENE: Gene-centric Metabolomics Information Retrieval Tool

Sumana Srinivasan[1,*], Mano R. Maurya[1,*], Srinivasan Ramachandran[1], Eoin Fahy[1] and Shankar Subramaniam[1,†]

[1]Department of Bioengineering, University of California, San Diego, La Jolla, CA 92093

[†]shsubramaniam@ucsd.edu
[*]Contributed equally.

Abstract

Background Biomedical research often involves contextual integration of multi-modal and multi-omic data in search of mechanisms for improved diagnosis, treatment and monitoring. Researchers need to access information from diverse sources, comprising data in various and sometimes incongruent formats. The downstream processing of the data to decipher mechanisms by reconstructing networks and developing quantitative models warrants considerable effort. **Results** MetGENE is a knowledge-based, gene-centric data aggregator that hierarchically retrieves information about the gene(s), their related pathway(s), reaction(s), metabolite(s), and metabolomic studies from standard data repositories under one dashboard to enable ease of access through centralization of relevant information. We note that MetGENE focuses only on those genes that encode for proteins directly associated with metabolites. All other gene-metabolite associations are beyond the current scope of MetGENE. Further, the information can be contextualized by filtering by species, anatomy (tissue) and condition (disease or phenotype). **Conclusions** MetGENE is an open-source tool that aggregates metabolite information for a given gene(s) and presents them in different computable formats, e.g., JSON, for further integration with other omics studies. MetGENE is available at https://bdcw.org/MetGENE/index.php.

Key words: metabolomics workbench; gene-centric; data aggregator; web application

Introduction

Recent advances in high-throughput technologies have led to many high-resolution multiomic measurements available to biomedical researchers. However, obtaining biological insights remains challenging since considerable effort is required to find and access data from diverse sources, deal with diverse and sometimes incomplete data formats, and tease out the connections within those high-dimensional datasets. This has led to an initiative by the US National Institutes of Health (NIH) called the Common Fund Data Ecosystem (CFDE), which aims to provide a single portal that makes data findable, accessible, interoperable and re-usable (FAIR) across the data repositories maintained by Data Coordination Centers (DCCs). Some examples of DCCs, include the Metabolomics Workbench (MW), which is a national metabolomics data repository [1], Genotype-Tissue Expression (GTEx) Project, a comprehensive resource to study tissue-specific gene expression and regulation [2], and the Library of Integrated Network-Based Cellular Signatures (LINCS) with the goal of generating a large-scale and comprehensive catalog of perturbation-response signatures by utilizing a diverse collection of perturbations across many model systems and assay types [3]. MW is a comprehensive resource hosting more than 2000 curated metabolomics studies and provides an integrated environment for data analysis and visualization through a suite of tools and interfaces to facilitate gaining biological insights.

A gene is a fundamental unit of query in the multi-omics data hierarchy. One of the goals of CFDE is to make every DCC support gene-centric querying within their repositories. Currently, MW supports a limited capability to perform gene-centric queries on the studies. MetGENE was designed to bridge this gap and enhance the capability by allowing a user to specify a gene or a set of genes that code for the metabolic enzyme(s) as a search term and, in re-

Compiled on: June 14, 2023.
Draft manuscript prepared by the author.

Key Points

- Knowledge-based data aggregator.
- Gene-centric query.
- Metabolomics Workbench studies.

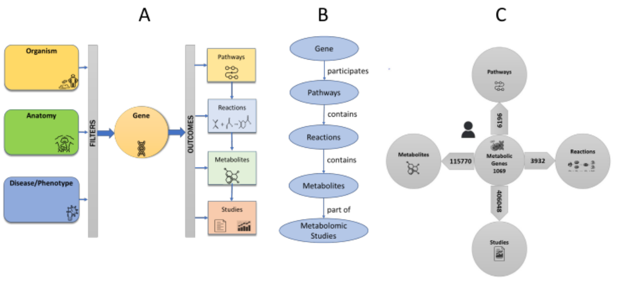

Figure 1. A. Gene(s) search is contextualized by the organism (species). The gene-associated pathways, reactions, metabolites and their corresponding metabolomic studies are reported as outcomes. Metabolites and Studies information can be filtered using anatomy (sample source), disease or phenotype. B. The knowledge graph underlying MetGENE. C. The number of associations for each relation in MetGENE.

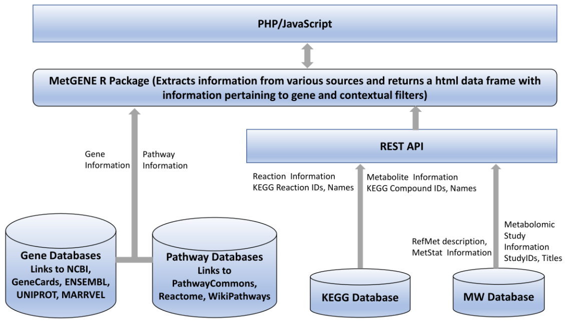

Figure 2. The Architecture of MetGENE comprises server-side PHP and JavaScript interacting with R scripts that use REST APIs to extract information from KEGG and MW databases. The gene and pathway information links are generated for specific repositories.

turn, fetch the relevant information from sources like the Kyoto Encyclopedia of Genes and Genomes (KEGG) [4] and the MW. Given one or more genes, the MetGENE tool identifies associations between the gene(s) and the metabolites (biosynthesized/catabolized or transported by proteins coded by the genes) and the reactions and pathways involving these metabolites. For each metabolite, studies containing the metabolite are identified from the MW. The results are organized as a gene landing page or a Dashboard, with all the information presented in a user-friendly manner to enable further analyses. There are many other ways a gene may have an "association" with a small molecule metabolite, such as via gene regulation (e.g., TF-target relationship) or its protein product, gene expression-metabolite association, protein-protein-metabolite association, etc. However, in MetGENE, we only focus on those genes that encode for proteins directly associated with metabolites; these include metabolic enzymes, transporters and receptors. .

Methods

MetGENE is a hierarchical, knowledge-based gene-centric information retrieval tool. Given a gene or a set of genes as a search term, MetGENE returns entities associated with the gene(s), namely pathways, reactions, metabolites and metabolomic studies in MW, as shown in Figure 1. MetGENE contextualizes the search by allowing the users to specify filters based on organism name, anatomy or tissue name (broadly, sample source), disease/phenotype as a part of its query interface, as shown in Figure 1A. A knowledge graph represents a network of entities, such as objects or concepts, and depicts their relationship. The knowledge graph that underlies information retrieval in MetGENE is depicted in Figure 1B. Further, for the human species, the number of metabolic genes, gene-pathway, gene-reaction, gene-metabolite and gene-metabolomic study associations found in MW are enumerated in Figure 1C.

MetGENE is designed as a web-based application using PHP and JavaScript as the front-end. The back-end contains R scripts with wrapper functions to retrieve information from various data repositories, such as KEGG [4] (for reaction and metabolite/compound IDs) and Metabolomics Workbench (for metabolite study IDs and RefMet names), as shown in Figure 2. The KEGG database provides the KEGG REST API to access information from the KEGG database. For any given gene, MetGENE supports SYMBOL, ENTREZ ID, RefSeq, UniProt, Ensembl and ALIAS (SYMBOL_OR_ALIAS) formats and converts IDs using an in-house Gene ID Conversion Tool (GICT). The GICT uses R Bioconductor packages, org.Xy.eg.db (e.g., org.Hs.eg.db for human) and NCBI gene_info table to convert the gene IDs. If the ID type for the input term is SYMBOL_OR_ALIAS, then the term is first searched in SYMBOL. If not found, then it is searched in ALIAS. Given the three-letter KEGG organism code and the ENTREZ gene ID (of the input gene term from the GICT), the R KEGGREST API provides a way to access all the information, such as pathway IDs, reaction IDs and compound (metabolites) IDs as a data frame object which is parsed further to display relevant information. The KEGG compound ID, along with the filter information pertaining to the species, anatomy and disease/phenotype, is used to extract information such as RefMet names and Study IDs using the MW REST API. RefMet names provide a standardized reference nomenclature for both discrete metabolite structures and metabolite species identified in metabolomic experiments. This is an essential prerequisite for comparing and contrasting metabolite data across different experiments and studies. For efficiency and to speed up the display, MetGENE caches the number of pathways, reactions, metabolites and studies associated with a particular gene which is updated weekly to accommodate new studies being deposited into MW.

MetGENE maintains session variables for species ID and organism name; ENTREZ gene ID and gene symbol; anatomy, disease/phenotype terms, and the previous values of these terms to enable server-side caching of pages and thus avoid unnecessary and time-consuming fetching of data across the network. The MetGENE back-end R functions are packaged into a library called metgene and will be available on GitHub for download. For programmatic ease of access, we provide REST APIs that output each information table displayed in JSON or CSV formats. The REST APIs are developed using Smart/Open API format [5].

Results

In this section, we describe the user experience starting from the MetGENE Query Page and ending with MetGENE Studies Page containing metabolomic studies corresponding to the gene in the

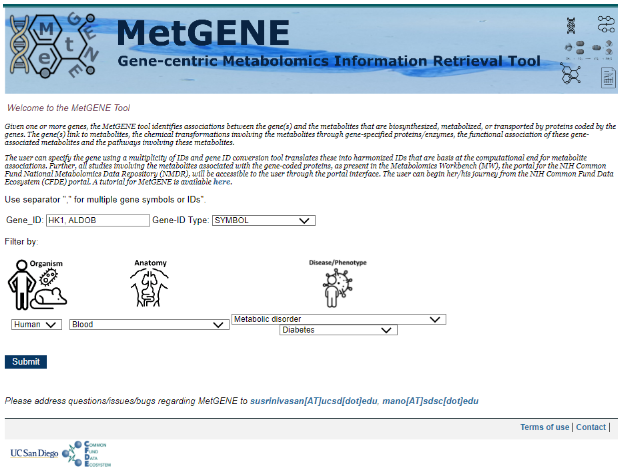

Figure 3. MetGENE Query page with the organism, anatomy (sample source), disease/phenotype-specific filters.

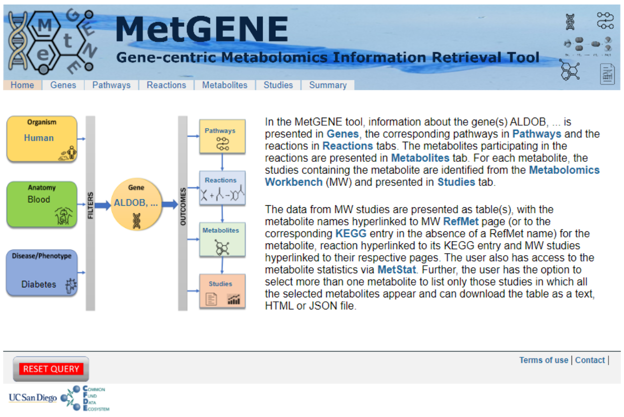

Figure 4. MetGENE landing page with context-sensitive display and access to Gene, Pathway, Reactions, Metabolites, Study and Summary information.

Metabolomics Workbench, incorporating various intermediate views of interest based on the knowledge graph described earlier.

MetGENE Query Interface

The user can input the gene information as a gene ID in any one of the formats described in the previous section. The format of the query page is as shown in Figure 3. The gene search input is validated on the client-side to allow only alphanumeric symbols. Invalid gene IDs are recognized, and appropriate error messages are displayed. MetGENE uses terms (e.g., Human, Mouse) for taxonomy filtering as per the NCBI taxonomy database (Coordinators, 2000). Currently, MetGENE supports human (*H.sapiens*), mouse (*M. musculus*) and rat (*R.norvegicus*) species, and we plan to add *E.coli* (K12), *C.elegans*, common fruit fly (*D.melanogaster*), and mosquito (*A.gambiae*) in the near future. For filtering the information on metabolites and studies by anatomy/tissue (e.g., Liver, Blood) and disease/phenotype (e.g., Diabetes, Fatty liver disease), terms from Metabolomics Workbench are used. Internally, the MW database records disease and phenotype under the metadata field, *disease*. Hence, the phenotype is searched as a disease term internally. The JSON files for each filter/category are curated and updated regularly and used to generate a pull-down menu. For the disease/phenotype filter, a two-step selection menu with slim (or disease class) terms in the first level and fine-grained terms in the second level is used for ease of presenting the options to the user. The user inputs from this page (main landing page) are submitted as a form, and a second page for MetGENE (as shown in Figure 4) is populated with the context-specific filtering terms. The second page comprises tabs for the search term associated entities, "Genes", "Pathways", "Reactions", "Metabolites", "Studies" and "Summary". The Summary tab displays the total number of pathways, reactions, metabolites and studies corresponding to each gene in the query. As MetGENE supports only those genes that encode for proteins directly associated with metabolites, a warning is issued if the queried gene or a set of queried genes does not encode for such proteins.

Gene and Pathway Information Pages

The gene information page shown in Figure 5a presents gene IDs in different formats hyperlinked to the corresponding web pages pointing to repositories such as KEGG [4], GeneCards [6], NCBI [7], Ensembl [8], UniProt [9] and Marrvel [10]. The URLs to these repositories for the specific genes are constructed based on their base URLs and the respective supported gene ID types. This information

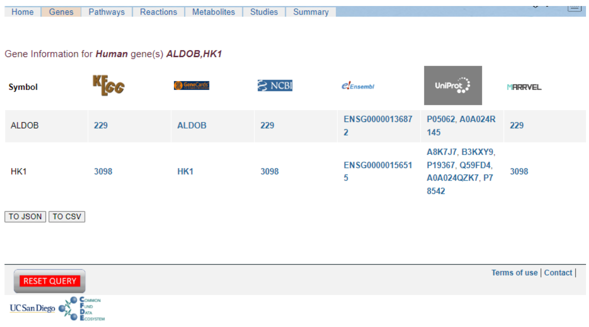

(a) Genes tab view.

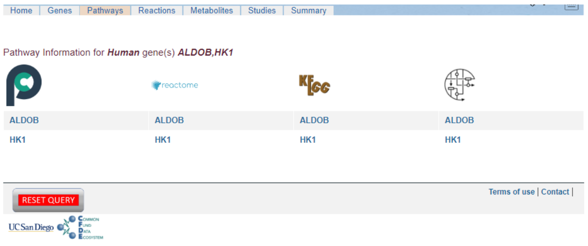

(b) Pathways tab view.

Figure 5. (a) MetGENE gene information page comprising Gene IDs in various formats corresponding to the searched gene(s) hyperlinked to various online resources. (b) MetGENE pathway information page comprising Gene IDs hyperlinked to various pathway resources.

is obtained from the REST API of the GICT and converted from JSON to a HTML table format for display purposes.

The pathway information page (Figure 5b) displays gene symbols hyperlinked with species and gene ID or symbol information as appropriate to various well-maintained pathway databases such as Pathway Commons [11], Reactome [12], KEGG [4] and Wikipathways [13]. MetGENE provides context-specific ease of access to these online resources.

Reaction and Metabolite Information Pages

The KEGG database provides the KEGG REST API to access information from the KEGG database. Given the organism code and the gene ID, the R KEGGREST API provides a way to access all the information, such as pathway IDs, reaction IDs, reaction names, reaction equations and compound (metabolites) IDs which are displayed in a tabular format. Figure 6a depicts the reaction information tab corresponding to the metabolic gene(s) of interest in a table view (one per gene) comprising reaction IDs hyperlinked to the corre-

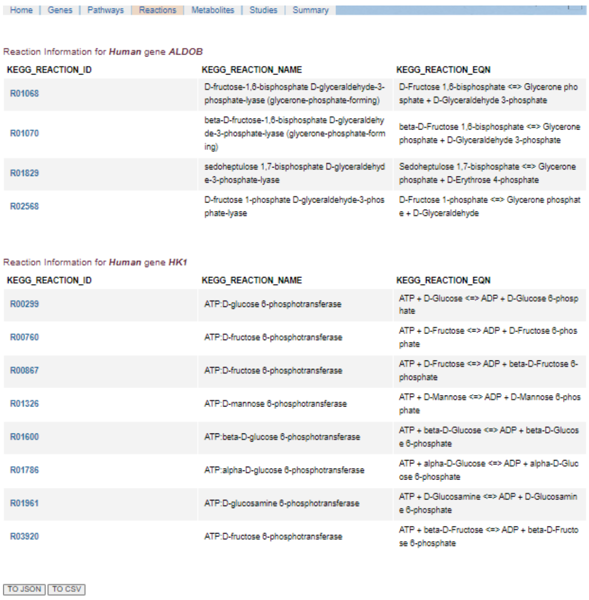

Home | Genes | Pathways | Reactions | Metabolites | Studies | Summary

Reaction Information for *Human* gene *ALDOB*

KEGG_REACTION_ID	KEGG_REACTION_NAME	KEGG_REACTION_EQN
R01068	D-fructose-1,6-bisphosphate D-glyceraldehyde-3-phosphate-lyase (glycerone-phosphate-forming)	D-Fructose 1,6-bisphosphate <=> Glycerone phosphate + D-Glyceraldehyde 3-phosphate
R01070	beta-D-fructose-1,6-bisphosphate D-glyceraldehyde-3-phosphate-lyase (glycerone-phosphate-forming)	beta-D-Fructose 1,6-bisphosphate <=> Glycerone phosphate + D-Glyceraldehyde 3-phosphate
R01829	sedoheptulose 1,7-bisphosphate D-glyceraldehyde-3-phosphate-lyase	Sedoheptulose 1,7-bisphosphate <=> Glycerone phosphate + D-Erythrose 4-phosphate
R02568	D-fructose 1-phosphate D-glyceraldehyde-3-phosphate-lyase	D-Fructose 1-phosphate <=> Glycerone phosphate + D-Glyceraldehyde

Reaction Information for *Human* gene *HK1*

KEGG_REACTION_ID	KEGG_REACTION_NAME	KEGG_REACTION_EQN
R00299	ATP:D-glucose 6-phosphotransferase	ATP + D-Glucose <=> ADP + D-Glucose 6-phosphate
R00760	ATP:D-fructose 6-phosphotransferase	ATP + D-Fructose <=> ADP + D-Fructose 6-phosphate
R00867	ATP:D-fructose 6-phosphotransferase	ATP + D-Fructose <=> ADP + beta-D-Fructose 6-phosphate
R01326	ATP:D-mannose 6-phosphotransferase	ATP + D-Mannose <=> ADP + D-Mannose 6-phosphate
R01600	ATP:beta-D-glucose 6-phosphotransferase	ATP + beta-D-Glucose <=> ADP + beta-D-Glucose 6-phosphate
R01786	ATP:alpha-D-glucose 6-phosphotransferase	ATP + alpha-D-Glucose <=> ADP + alpha-D-Glucose 6-phosphate
R01961	ATP:D-glucosamine 6-phosphotransferase	ATP + D-Glucosamine <=> ADP + D-Glucosamine 6-phosphate
R03920	ATP:D-fructose 6-phosphotransferase	ATP + beta-D-Fructose <=> ADP + beta-D-Fructose 6-phosphate

TO JSON | TO CSV

(a) Reactions tab view.

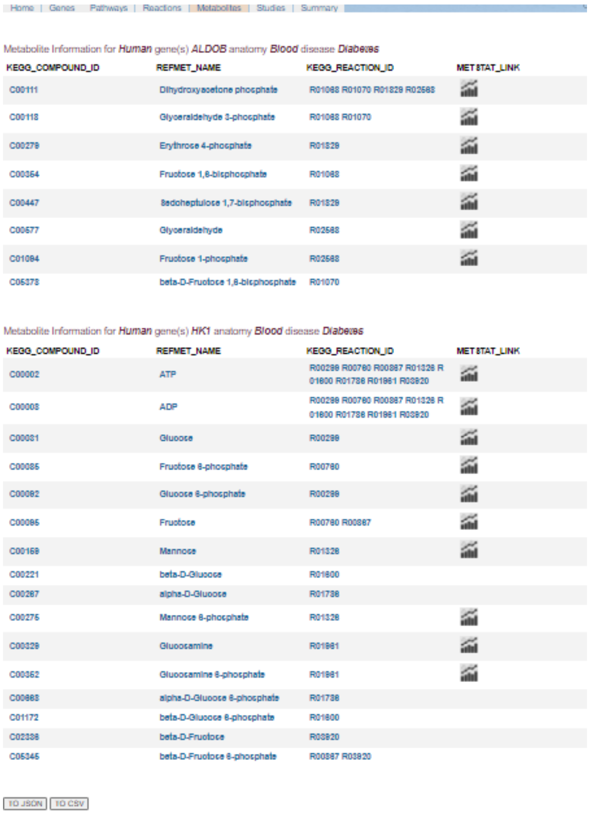

Home | Genes | Pathways | Reactions | Metabolites | Studies | Summary

Metabolite Information for *Human* gene(s) *ALDOB* anatomy *Blood* disease *Diabetes*

KEGG_COMPOUND_ID	REFMET_NAME	KEGG_REACTION_ID	METSTAT_LINK
C00111	Dihydroxyacetone phosphate	R01068 R01070 R01829 R02568	
C00118	Glyceraldehyde 3-phosphate	R01068 R01070	
C00279	Erythrose 4-phosphate	R01829	
C00354	Fructose 1,6-bisphosphate	R01068	
C00447	Sedoheptulose 1,7-bisphosphate	R01829	
C00577	Glyceraldehyde	R02568	
C01094	Fructose 1-phosphate	R02568	
C05378	beta-D-Fructose 1,6-bisphosphate	R01070	

Metabolite Information for *Human* gene(s) *HK1* anatomy *Blood* disease *Diabetes*

KEGG_COMPOUND_ID	REFMET_NAME	KEGG_REACTION_ID	METSTAT_LINK
C00002	ATP	R00299 R00760 R00867 R01326 R01600 R01786 R01961 R03920	
C00008	ADP	R00299 R00760 R00867 R01326 R01600 R01786 R01961 R03920	
C00031	Glucose	R00299	
C00085	Fructose 6-phosphate	R00760	
C00092	Glucose 6-phosphate	R00299	
C00095	Fructose	R00760 R00867	
C00159	Mannose	R01326	
C00221	beta-D-Glucose	R01600	
C00267	alpha-D-Glucose	R01786	
C00275	Mannose 6-phosphate	R01326	
C00329	Glucosamine	R01961	
C00352	Glucosamine 6-phosphate	R01961	
C00668	alpha-D-Glucose 6-phosphate	R01786	
C01172	beta-D-Glucose 6-phosphate	R01600	
C02336	beta-D-Fructose	R03920	
C05345	beta-D-Fructose 6-phosphate	R00867 R03920	

TO JSON | TO CSV

(b) Metabolites tab view.

Figure 6. (a) MetGENE reaction information page comprising KEGG Reaction IDs hyperlinked to KEGG reaction information page and reaction descriptions in a tabular format. (b) MetGENE metabolite information page comprises KEGG compound IDs, MW RefMet names, reaction IDs in which a metabolite participates in, hyperlinked to the KEGG reaction information page and MetStat link for the metabolite in a tabular format.

sponding KEGG reaction information page, reaction names and the reaction equation.

In the metabolites information tab, as shown in Figure 6b, a unique list of metabolites across all reactions corresponding to a given gene, along with their respective RefMet names (MW provides REST APIs to access the RefMet name corresponding to a KEGG compound ID), KEGG reaction IDs of all the reactions the metabolite participates in, are displayed. Further, the MW MetStat link provides access to the statistics about the metabolite measured across various studies in the MW database, filtered by anatomy (sample source) and disease/phenotype. This tool generates a report for any given metabolite in MW, comprising all the unique studies containing that metabolite and their median value of the relative standard deviation (RSD) across all those studies. The KEGG compound IDs that do not have RefMet names in the MW database display only the KEGG compound name and reaction IDs. The KEGG compound IDs are hyperlinked to the corresponding KEGG compound information page. MetGENE allows users to download the tables directly from the displayed page in JSON or CSV formats for further analysis. MW also provides REST APIs to access all the study IDs, titles, and RefMet names for a given KEGG compound ID in JSON, text and HTML formats.

MetGENE Metabolomics Study Information Page

In the Studies page (as shown in Figure 7a), a tabular view of the unique list of metabolites for the queried gene(s), their RefMet names hyperlinked to their corresponding description page in MW, and a comma-separated list of study IDs in which the metabolite participates (with each study ID linked to its corresponding study description page in MW) is presented. Further, a helpful text hover feature displaying the study title corresponding to a particular study ID is also provided to the user. MetGENE also allows users to select metabolites of interest and combine their studies for download and further analysis, as shown in Figure 7b.

MetGENE Summary Page

In the Summary page (as shown in Figure 8), MetGENE displays the total number of pathways, reactions, metabolites and metabolomic studies for each queried gene and displays the information in a tabular format and a pie chart. This information is available for download both in JSON and CSV formats.

Case Study: Exploring gene PNPLA3 using MetGENE

Here, we demonstrate a use case that shows the utility of MetGENE as a one-stop tool to obtain all metabolomic information associated with a gene(s) in a specific disease condition. The protein Adiponutrin, encoded by the gene PNPLA3, is a multi-functional enzyme that belongs to the IPLA2/lipase family, which has both triacylglycerol lipase and acylglycerol O-acyltransferase activities. PNPLA3 is predominantly expressed in adipocytes and liver cells. It regulates the development of adipocytes and the metabolism of fats (lipogenesis and lipolysis). Diseases associated with PNPLA3 mutations include Fatty Liver Disease and Non-Alcoholic Steatohepatitis (NASH) [14],[15], [16] and [17].

To obtain information about a gene(s) and associated entities like enzymes, reactions, pathways and existing metabolomic studies in MW for a given context (fatty liver disease in humans), a user needs to identify precise key terms, perform a search on the internet, sift through the results to identify literature about various metabolomic studies and download the studies to perform downstream analyses. These steps are sometimes time-consuming and misleading, depending on the search terms' specificity. However, with MetGENE, the user can specify a gene ID(s) in any popular for-

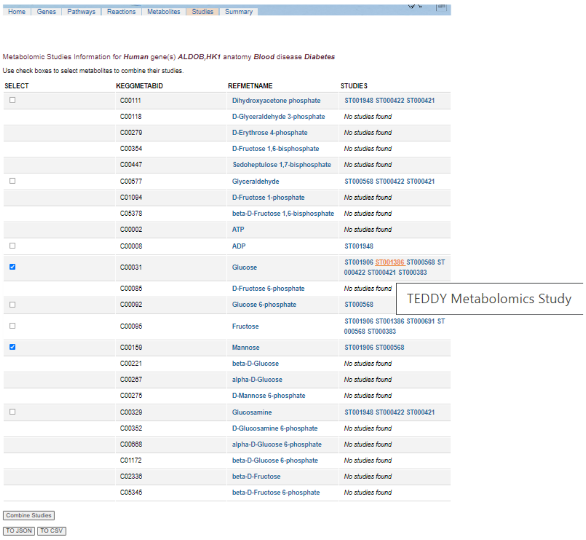

(a) Studies tab view.

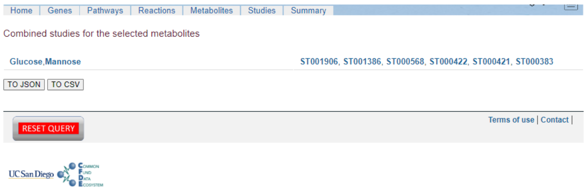

(b) Combined studies tab view.

Figure 7. (a) MetGENE metabolomics studies information page comprises KEGG compound IDs, RefMet names, and MW study IDs corresponding to a metabolite in a tabular format. (b) MetGENE allows users to combine studies for a selected set of metabolites.

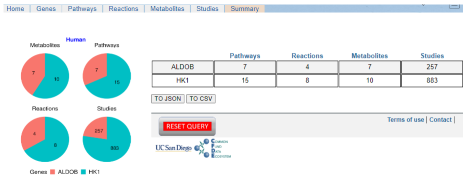

Figure 8. The Summary tab in MetGENE enumerates the total number of pathways, reactions, metabolites and metabolomic studies corresponding to each queried gene.

mat and apply the filters on organism, anatomy, disease/phenotype to obtain genes, pathways, reactions, metabolites and studies information consolidated from various data resources, at one go. Supplementary Figure A1 shows the Gene tab for the PNLPA3 Gene. Links to various online resources (GeneCards, KEGG, NCBI, Uniprot and Marrvel) with genomic, transcriptomic, proteomic, clinical and functional information (via GeneCards link), module and pathway information (via KEGG), transcript and region information (via NCBI link), sequence, variant, gene expression information (via Ensembl link), protein sequence and functional information (via Uniprot link) and gene variants associated with PNLPA3 (via Marrvel link) are provided in the gene information table. Supplementary Figure A2 represents the Pathway tab for the PNLPA3 gene with links to interactions with and pathways that involve PNPLA3 (via Pathway Commons), protein and reaction location information (via Reactome), pathway definitions (via KEGG), and pathway-related collaborative information (via Wikipathways). Supplementary Figure A3 represents the Reactions and Metabolites tabs for PNPLA3. It delineates two hydrolysis reactions where diacylglycerol (DAG) is hydrolyzed to 2-monoacylglycerol (2-MAG), and triacylglycerol (TAG) is hydrolyzed to DAG by PNPLA3. Three generic compounds (TAG, DAG, and Fatty acid) participate in the reactions. The Metabolites tab for PNPLA3 lists all the metabolites (with substitutions for TAG and DAG) along with corresponding RefMet names or KEGG metabolite names (in the absence of RefMet names) along with a MetStat link that points to metabolite statistics information such as a histogram of the RSD (Relative Standard Deviation) metabolite data, ANOVA results for the metabolite with a cut-off p-value in MW via MetStat, with the anatomy and disease filters applied. Supplementary Figure A4 depicts the Studies tab in MetGENE for the gene PNPLA3. Each metabolite would display the corresponding Study IDs if measured/listed in any studies deposited in MW, hyperlinked to the study description. A hover text displays the study title. The tool provides the user with the ability to combine studies for metabolites of interest to a consolidated view, as shown in Supplementary Figure A4. Supplementary Figure A5 serves as an informative visual aid, providing an overview of the information flow network within MetGENE for the PNPLA3 use case. It simplifies the complexity of the underlying technical details of MetGENE, facilitating comprehension of the query-to-results process. All of MetGENE tables can be downloaded in formats such as JSON and CSV directly from the respective pages in the browser or via the REST API. The REST API supports JSON and text formats. They are deposited in the Smart API repository along with the accompanying documentation.

Discussion

Given a gene(s), the MetGENE tool identifies associations between the gene(s) and the metabolites that are biosynthesized, catabolized, or transported by proteins coded by those gene(s). It is a knowledge-based data aggregator, accessing and integrating data from resources such as the KEGG and Metabolomics Workbench. The gene(s) link to metabolites, the chemical transformations involving the metabolites through gene-specified proteins/enzymes, the functional association of these gene-associated metabolites and the pathways involving these metabolites with context-based filtering based on anatomy (sample source), disease/phenotype. The user can specify the gene using a multiplicity of IDs, and the gene ID conversion tool translates these into harmonized IDs that are the basis for metabolite associations. Further, all studies involving the metabolites associated with the gene-coded proteins, as present in the Metabolomics Workbench (MW), will be accessible to the user as a stand-alone tool or via the portal interface for the NIH Common Fund National Metabolomics Data Repository (NMDR). The user can begin their journey either from the main web page for MetGENE (see Availability) or from the NIH Common Fund Data Ecosystem (CFDE) portal (https://app.nih-cfde.org/; the steps are: Data Browser → Vocabulary → Gene).

Potential implications

Features from MetGENE will contribute to the integration of other omics data to draw metabolomics perspectives and with metabolomics data, with genes serving as the bridging nodes. For example, tools such as MetGENE will assist a researcher in interpreting the results of multi-omics data integration holistically, where they can consider both gene-related and metabolomics data in a metabolic pathway. We are also in the process of integrating MetGENE with tools from other DCCs as a part of a broader NIH-CFDE initiative. Through that, we can provide support for persistent and shareable MetGENE-based workflows.

Availability of Supporting Code and Requirements

MetGENE is an open-source collaborative initiative available at GitHub. The main website of MetGENE and the Smart APIs are available, as shown below. MetGENE is registered with the SciCrunch registry (RRID:SCR_023402 (https://scicrunch.org)). We also plan to register MetGENE-based workflows as BioCompute Objects (BCOs) in the Galaxy Community Hub (https://galaxyproject.org/) in the near future as a part of NIH-CFDE efforts.

- Project Name: MetGENE
- Project Home Page: https://bdcw.org/MetGENE/index.php
- Operating System: Platform independent
- Programming languages: R and PHP
- Smart APIs: https://smart-api.info/registry/?q=MetGENE
- Source code: https://github.com/metabolomicsworkbench/MetGENE

Additional Files

- Supplementary Figure A1: The gene information tab for PNPLA3 in MetGENE has links to various online resources that provide different types of information pertaining to the gene, including sequence, gene expression, protein structure, functional and disease information.
- Supplementary Figure A2: The pathway information tab for PNPLA3 in MetGENE has links to various online resources that provide different types of information pertaining to the pathways in which PNPLA3 participates.
- Supplementary Figure A3: The reaction and metabolite information tabs for PNPLA3 in MetGENE have links to reactions controlled by PNLPA3, with Reaction IDs linked to the KeggKEGG reaction definition page. RefMet provides the metabolite details, and the MetStat link provides the context-specific metabolite measurement statistics.
- Supplementary Figure A4: The studies information tab for PNPLA3 in MetGENE has links to studies in MW pertaining to the metabolites listed. Studies can be combined for selected metabolites for further analysis.
- Supplementary Figure A5: A consolidated network of information flow for gene PNPLA3 demonstrating how pathways, reactions, metabolites, studies and summary are connected in the result.

Declarations

List of abbreviations

- CFDE - Common Fund Data Ecosystem
- DCC - Data Coordination Centers
- GTEx - Gene Tissue Expression
- LINCS - Library of Integrated Network-Based Cellular Signatures
- GICT - Gene ID Conversion tool
- MW - Metabolomics Workbench
- NMDR - National Metabolomics Data Repository

Ethical Approval

Not Applicable.

Consent for publication

Not Applicable.

Competing Interests

Not Applicable.

Funding

This work has been supported by the National Institutes of Health Grants (Metabolomics Workbench, U2C-DK119886 and Common Fund Data Ecosystem (CFDE) OT2-OD030544).

Author's Contributions

This work was conceptualized by Sh.S., Su.S., M.R.M. and S.R. The funding acquisition was by Sh.S. The methodology, software development and visualization for MetGENE were developed by Su.S. and M.R.M. Validation and testing was performed by Su.S., M.R.M., S.R. and Sh.S. The disease ontology terms and structuring was provided by S.R. The Metabolomics Workbench REST APIs that are used in this work were developed by E.F. The original draft for the paper was prepared by Su.S and reviewing and editing was done by Sh.S, M.R.M. and S.R. The project administration was done by Sh.S and M.R.M.

Acknowledgements

We thank the Common Fund Data Ecosystem Gene Working Group for useful discussions.

References

1. Sud M, Fahy E, Cotter D, Azam K, Vadivelu I, Burant C, et al. Metabolomics Workbench: An international repository for metabolomics data, metadata and metabolite standards, protocols, tutorials and training, and analysis tools. Nucleic acids research 2016;44:D463–70. http://www.metabolomicsworkbench.org.
2. GTExConsortium. Human genomics. The Genotype-Tissue Expression (GTEx) pilot analysis: multitissue gene regulation in humans. Science 2015;348(6235):648–660. https://gtexportal.org/home/.
3. V S, J T, A K, D V, D C, M FN, et al. LINCS Data Portal 2.0: next generation access point for perturbation-response signatures. Nucleic Acids Research 2020;48:D431–D439.
4. Kanehisa M, Goto S. KEGG: kyoto encyclopedia of genes and genomes. Nucleic Acids Research 2000;28(1):27–30. https://www.genome.jp/kegg/kegg1.html.
5. Schloerke B, Allen J. plumber: An API Generator for R; 2022, https://www.rplumber.io, https://github.com/rstudio/plumber.
6. Safran M, Rosen N, Twik M, BarShir R, Stein TI, Dahary D, et al. In: The GeneCards Suite Singapore: Springer Singapore; 2021. p. 27–56. https://doi.org/10.1007/978-981-16-5812-9_2.
7. S F. The NCBI Taxonomy database. Nucleic acids research. Nucleic Acids Research 2012;40:D136–D143. https://doi.org/10.1093/nar/gkr1178.
8. Cunningham F, Allen JE, Allen J, Alvarez-Jarreta J, Amode MR, Armean IM, et al. Ensembl 2022. Nucleic Acids Research 2022;50(1):D988–D995. https://doi.org/10.1093/nar/gkab1049.
9. Consortium TU. UniProt: the universal protein knowledgebase in 2021. Nucleic Acids Research 2021;49(D1):D480–D489. https://doi.org/10.1093/nar/gkaa1100.
10. Wang J, Al-Ouran, R, Hu, Y, Kim, et al. MARRVEL: Integration of Human and Model Organism Genetic Resources to Facilitate Functional Annotation of the Human Genome. Amer-

ican journal of human genetics 2021;100(6):843–853. https://doi.org/10.1016/j.ajhg.2017.04.010.
11. Cerami EG, Gross BE, Demir E, Rodchenkov I, Babur O, Anwar N, et al. Pathway Commons, a web resource for biological pathway data. Nucleic Acids Research 2010;39:D685–D690. https://www.pathwaycommons.org/.
12. Gillespie M, Jassal B, Stephan R, Milacic M, Rothfels K, Senff-Ribeiro A, et al. The reactome pathway knowledgebase 2022. Nucleic Acids Research 2021;50(D1):D687–D692. https://reactome.org/.
13. M M, A A, A R, A W, DN S, K H, et al. WikiPathways: connecting communities. Nucleic Acids Research 2021;49:D613–D621. https://doi.org/10.1093/nar/gkaa1024.
14. Cohen J, Horton J, HH H. Human fatty liver disease: old questions and new insights. Science 2011;332(6037):1519–23.
15. Gorden D, Myers D, Ivanova P, Fahy E, Maurya M, Gupta S, et al. Human fatty liver disease: old questions and new insights. Science 2011;332(6037):1519–23.
16. P P, S R. The role of PNPLA3 in health and disease. Biochem Biophys Acta Mol Cell Biol Lipids 2019;1864(6):900–906.
17. XC D. PNPLA3—A Potential Therapeutic Target for Personalized Treatment of Chronic Liver Disease. Front Med 2019;6(304).

Response to reviewers Click here to access/download;Manuscript;Response_to_Reviewers.docx

Main comments

Reviewer 1:

1. Overall the knowledge-base is well designed, with a description of the data retrieval mechanism on the query page. However, the term "associated" metabolites to a gene is rather open-ended. It seems that the association is via the encoded protein to a gene, and its known enzymatic activity. This then links the gene to a metabolite, and known pathways where the enzyme is active. There are many other ways that a gene may have an "association" with a small molecule metabolite however - such as via regulation of the gene or its protein product with small molecules metabolites, literature-based connections, gene expression-metabolite associations etc. It may help the user to know what associations are not a part of the analysis, so the user knows what to expect in terms of how information is retrieved from any given query.

Response: We thank the reviewer for this important comment. We have added the following line in the Abstract section, "MetGENE focuses only on those genes that encode for proteins directly associated with metabolites. All other gene-metabolite associations are beyond the current scope of MetGENE." We have also elaborated on this point in the Introduction section, "There are many other ways a gene may have an "association" with a small molecule metabolite, such as via gene regulation (e.g., TF-target relationship) or its protein product, gene expression-metabolite association, protein-protein-metabolite association, etc. However, in MetGENE, we only focus on those genes that encode for proteins directly associated with metabolites; these include metabolic enzymes, transporters and receptors.". We have included the number of associations supported by MetGENE in Figure1C.

2. Related to the point above, a key linkage of a gene to a metabolite is the encoded protein that has catalytic activity. Although it is possible to get to the information on the protein and its enzymatic activity via the Uniprot link via a couple of clicks by the user, it seems that the Uniprot link could be displayed more prominently on the initial results page after the query is entered. This may be a more efficient way for users to get information on the protein, as a key linkage point to the metabolite/pathway information.

Response: We thank the reviewer for this suggestion, and we agree that providing Uniprot link in the main screen may be of help to the user. However, for a given gene, there are several Uniprot IDs and displaying all this information in the main screen would lead to visual cluttering ad hence we decided to display this in the Genes tab along with other useful set of links such as KEGG, Ensembl, etc. which may provide more information to the user.

3. It is understood that the "phenotype" query is not available yet. However, it's unclear what type of queries will be available here. Could the authors provide some information on the options for phenotypes that they plan to make available?

Response: In the current version on MetGENE, we decided to completely do away with the Phenotype menu in the query screen. We have combined both the Disease as well as the Phenotype into a consolidated two step cascading menu list. We have updated Figure 3 in the manuscript to reflect this change. The Metabolomics Workbench does not recognize "Phenotype" as a separate search term and hence we decided to consolidate both disease and

phenotype into a single list. However, in the future, if MW decides to support querying based on the Phenotype terms from databases such as Human Phenotype Ontology, we will support it.

4. They authors should be more clear on exactly how many organisms can be queried - at this point it looks like it is a relatively limited number (human, mouse, rat). Are there plans to include any more organisms - such as pathogenic bacteria or other organisms related to human health that might be of interest?

<u>Response:</u> *We agree with the reviewer and our apologies for not making it clear in our manuscript. We will include microorganisms relevant to human health and disease in a phased manner. We plan to include E. coli (K12), C. elegans, D. melanogaster, and A. gambiae soon* and *have mentioned this in the Result sections of the manuscript*.

5. In test runs it might be nice to show the number of returned results under "Metabolites", "Reactions", "Pathways" in the first window. Some may be empty and it seems inefficient for the user to have to click through each category only to find out there are not any results?

<u>Response:</u> *We thank the reviewer for an excellent suggestion. Since the time taken to compute the number of results can be high in the case of multiple genes or genes who have large number of metabolites associated with them, adding this feature to the main landing page would slow down the loading of the page which is undesirable. We have created a "Summary" tab that displays the number of pathways, reactions, metabolites, and the studies both as a downloadable table as well as a pie chart graphic for better visualization (Figure 4). In addition, we have also added a feature where the tool warns the user about empty results if the gene does not encode a protein directly related to metabolites.*

6. The "clear query" button to start a new query is a bit small - this could be more obvious to the user to clear the form and start again.

<u>Response:</u> *We have increased the size and the graphic on the button to clear the query to address this issue.*

Reviewer 2:

1. There are many methods/databases/tools linking genes to pathways, reactions, and metabolites. For example, MetaCyc or HumanCyc provides web and local interfaces, where the search can start from genes, proteins, pathways, or metabolites. Related information can also be easily fetched (e.g., from genes to related reactions or metabolites). Those databases tend to be curated, which increases accuracy but can probably miss possible connections. This manuscript provides database searching that is not curated which can possibly provide more connections. However, such comparisons are not stated in the manuscript. In addition, many of the databases (KEGG, GeneCards) the authors used and mentioned have blocks of genes, pathways, and metabolites. The authors need to explain the significance and improvement of MetGENE upon other databases or curated tools. Is MetGENE going to provide more complete

results than any of them? How do MetGENE results tradeoff between coverage and accuracy compared with others?

<u>Response:</u> *MetGENE uses a knowledge graph to relate metabolic genes to the metabolites and then downstream to metabolomic studies. The main value proposition of MetGENE is to get studies from MW by providing a gene centric front end. MetGENE is not a database and does not compete with other databases that provides Gene → Pathways → Reactions → Metabolite connections but merely provides a context by becoming a one stop place for accessing other databases in context. Hence, we are not in a position to provide trade-off result comparing to other databases.*

2. It seems that the interface with the metabolic workbench is one of the major innovations of this manuscript. The tool and visualization provided for this functionality are however limited. It's mainly through the block of metabolites (METSTAT) and studies, which indeed seem to provide interesting information. Can the author show how MetGENE helps improve users' understanding or efficiency through such integration with a specific biological example? How does integrating results of multiple workbench studies show something not directly visible in one? Some additional functionality in this part can be helpful to the users (e.g., a table of quantification and corresponding metabolites approaches of the study through API OR a summary information table for the study included in the Study page).

<u>Response:</u> *At the moment, we are not providing any more extra insights by combining studies from MW. However, in the context of quantification of metabolites, METSTAT already provides summary (such as distribution of values of metabolites) based on information from multiple studies. Study summarization with respect to different types of metadata can be done (e.g., type of chromatography, MS/NMR etc). However, integration of information based on metadata (e.g., 6 studies were GC-MS, 4 Studies were LC-MS, etc.) may not be that useful. If we come across a use case that requires integration of multiple studies, we will incorporate it into MetGENE in the future.*

3. The presentation of this paper has been mainly qualitative. More quantitative evaluation and presentations can help. For example, what's the size of the search space of genes for MetGENE to have any results? How many reactions, metabolites, pathways, and studies are connected?

<u>Response:</u> *We thank the reviewer for the suggestion. Now, we support 1069 metabolic genes. We have represented statistics of the associations in Figure 1C.*

4. Only allowing starting from genes seems limited. The search should also be able to start with metabolites or reactions.

<u>Response:</u> *We agree with the suggestion but expanding it to start from Reaction or Pathway is out of the current scope of MetGENE. Also, it would be hard for users to specify the Reaction/Pathway name or description as a starting point to query. We also note that the*

Metabolomics Workbench (https://www.metabolomicsworkbench.org/databases/refmet/refmet.php) already enables connecting metabolites to genes, reactions, and pathways.

5. Even though explained in the method, it's not clear to me what kind of connections between genes, pathways, reactions, and metabolites the authors used for the results. A supplementary figure showing the logical process of searching starting from one gene can be helpful. Particularly, the user can present a network starting from one specific gene (e.g., PNPLA3) and present how different parts (e.g., metabolites, pathways) are connected in the result.

Response: As per the suggestion of the reviewer, we have added an additional figure in the Supplementary Material (Figure A5) that depicts the information flow from query to the results for the use case of the gene PNPLA3.

Minor comments:

6. The pathway result is puzzling as it seems to be links connected to different databases searching for information related to the genes. When I click those links, the information is not solely on pathways. Supplying all the information in a table rather than links can be much easier for the user. The WikiPathways link seems also not working. Similarly, the author can add more information for the tables of reactions (showing the reaction equation itself).

Response: The reason MetGENE is designed to display a list of Pathway database links as opposed to the pathways themselves is to enable the user to have access to different types of information each of these databases provide. One could potentially scrape this information and display consolidated information but to decide what information is useful to display or not would be difficult to assess. As per the reviewer's suggestion, we have included the reaction equation as part of the Reactions table (Figure 6a). We have also updated our REST and Smart APIs to reflect this.

7. It seems white space after the comma when searching for two genes will cause problems.

Response: We have fixed this issue in the current version of the tool. We *thank the reviewer for pointing it out.*

8. There are many technical details in the result which can be put into methods or supplements. For example, the sentence "Given the three-letter KEGG organism code and the ENTREZ gene ID of the gene, the R KEGGREST API provides a way to access all the information such as pathway IDs, reaction IDs and compound (metabolites) IDs as a data frame objects" can be simplified without mention code length, specific ID, specific API methods, etc. The "MetGENE Query Interface" section of the result also seems too detailed and technical. The result part can be rewritten and reduced to improve accessibility to general readers.

Response: We appreciate the reviewer's feedback regarding simplifying the writing. We have simplified the writing in this revised version of the manuscript (Methods section).

Click here to access/download;Figure;Figure1_Main.png

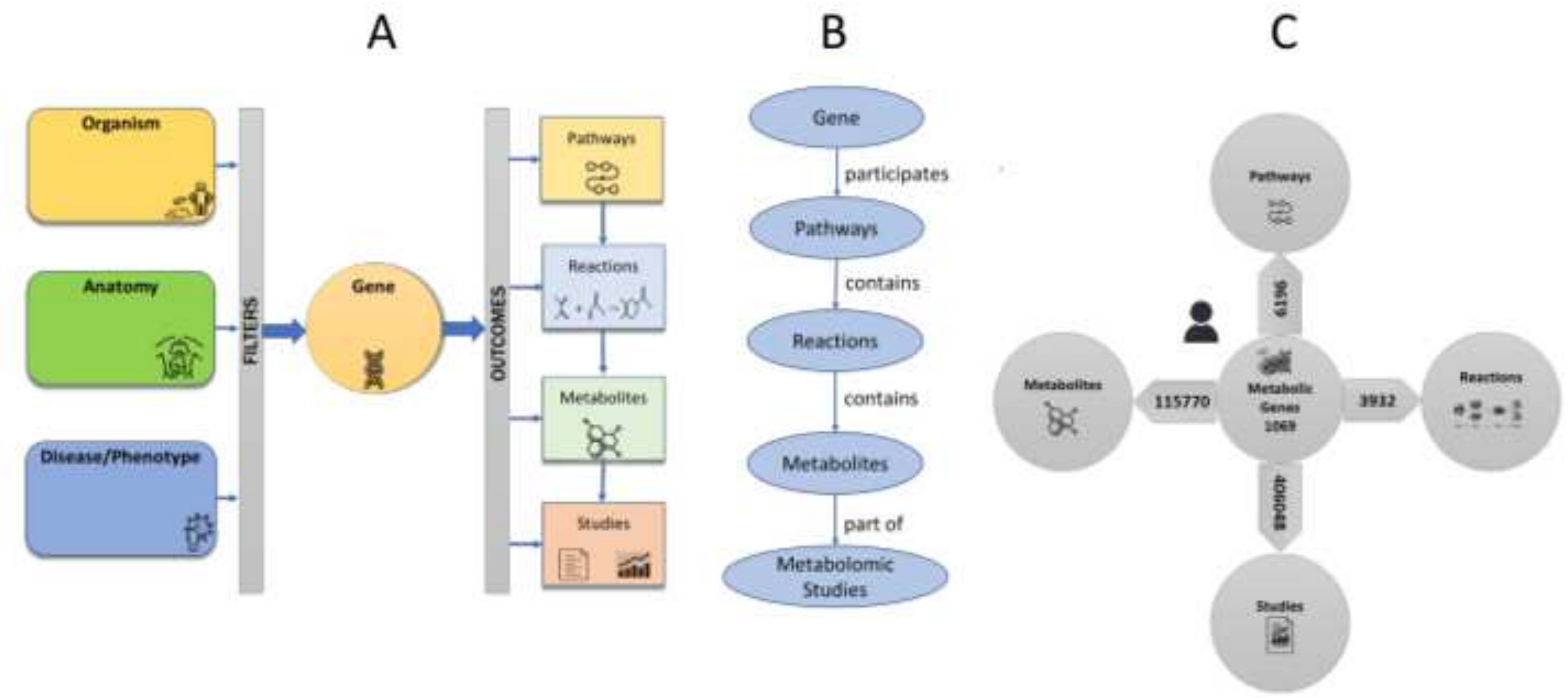

Query page

Click here to access/download;Figure;Figure3_Main.png

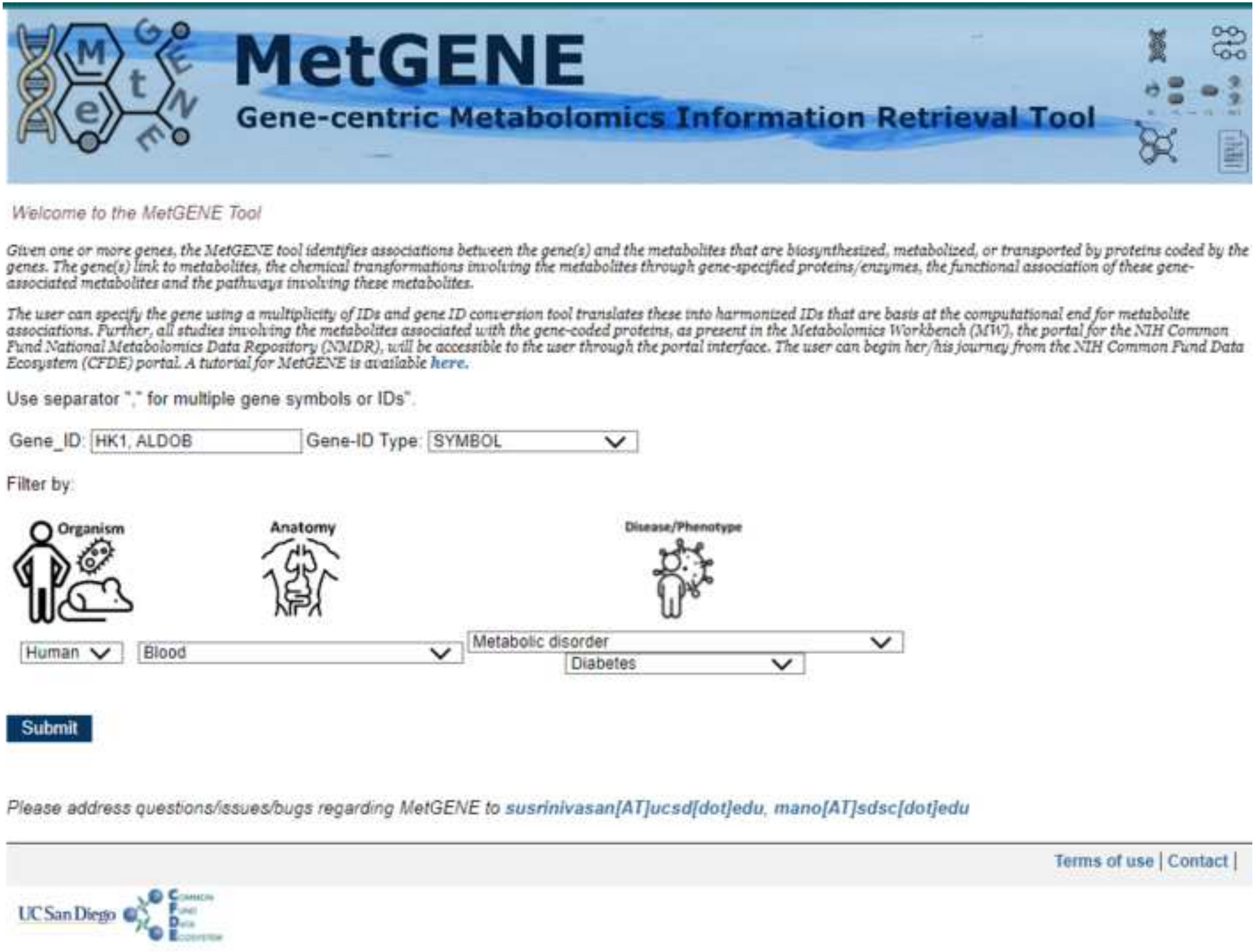

Landing Page

Click here to access/download;Figure;Figure4_Main.png

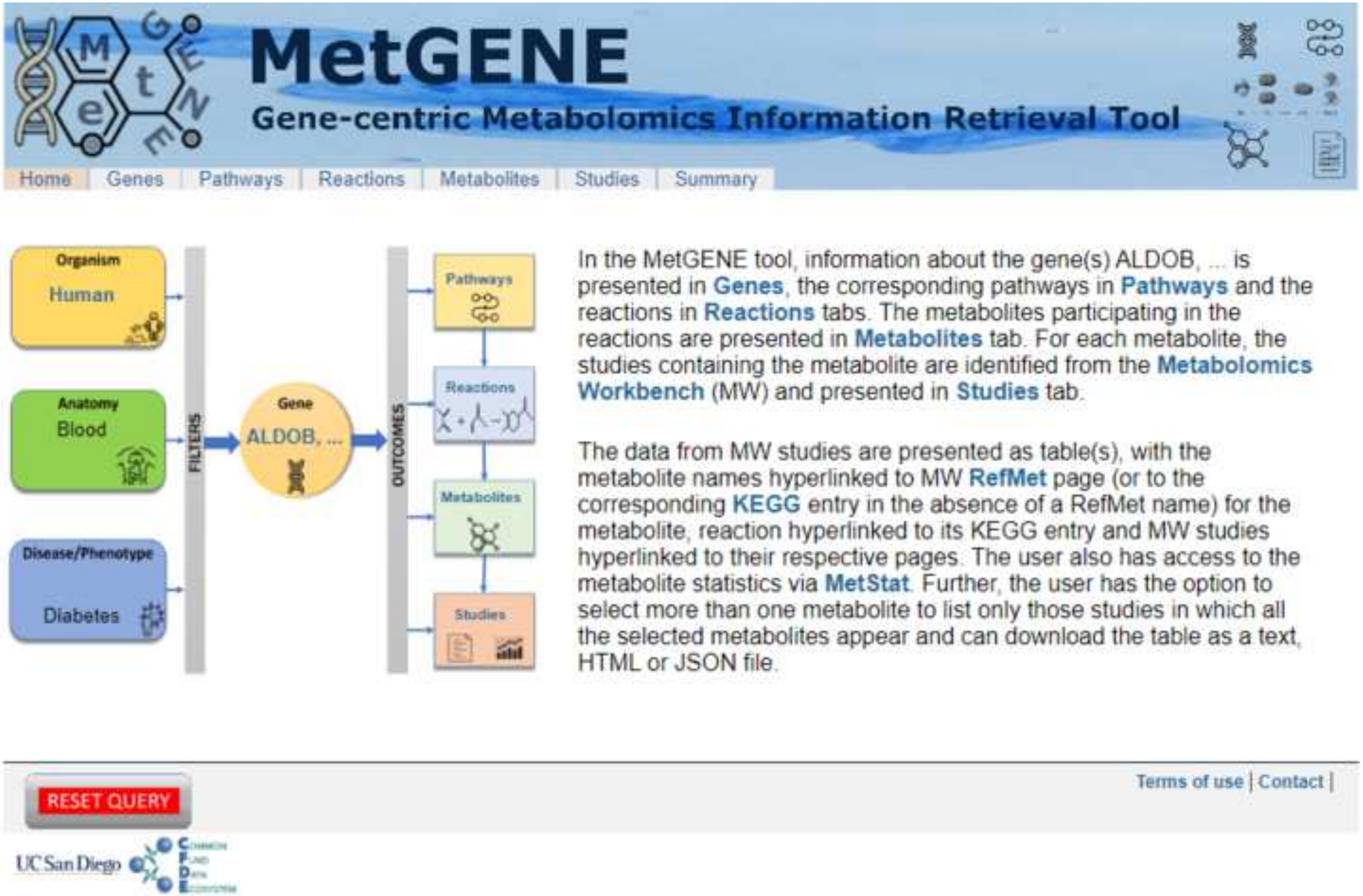

Genes Tab

Click here to access/download;Figure;Figure5A_Main.png

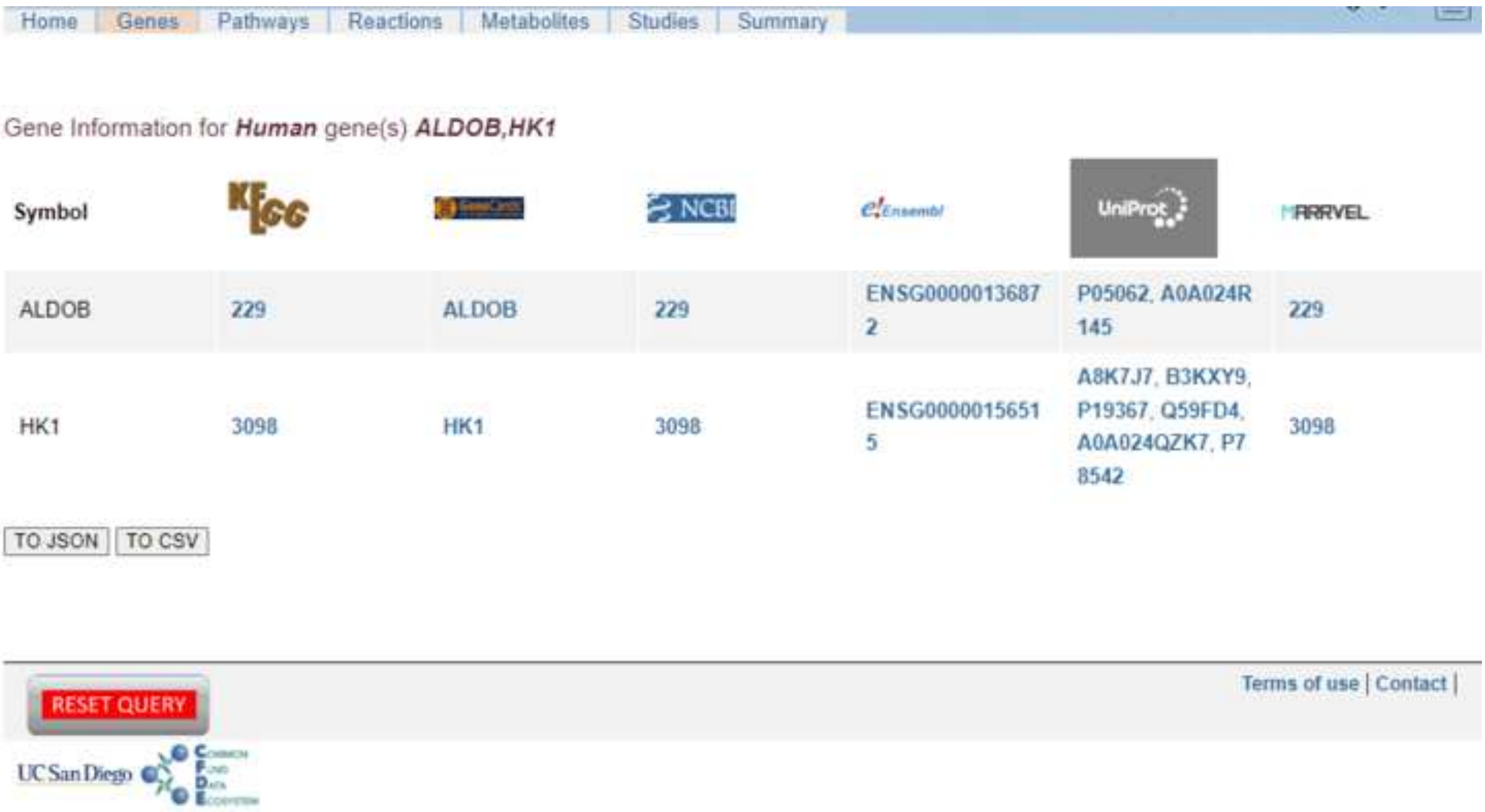

Pathways Tab

Click here to access/download;Figure;Figure5B_Main.png

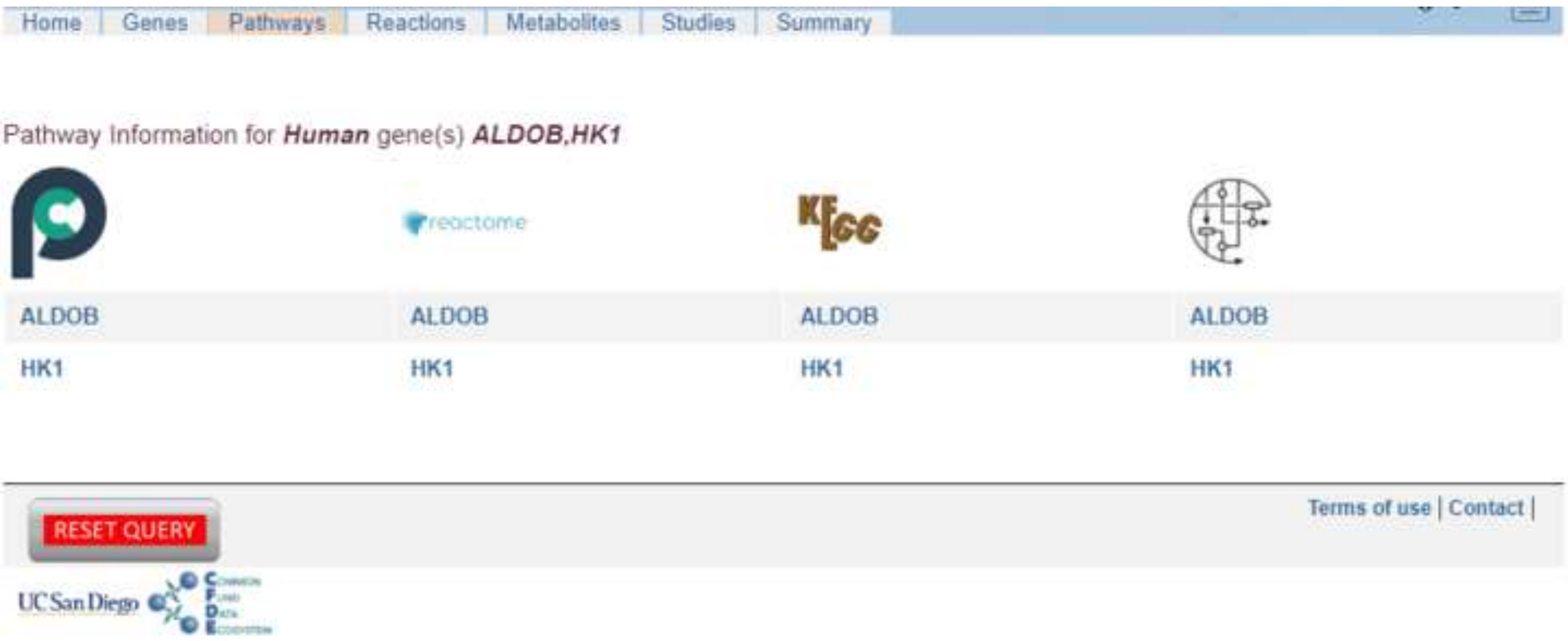

Reactions Tab

Click here to access/download;Figure;Figure6A_Main.png

Home | Genes | Pathways | Reactions | Metabolites | Studies | Summary

Reaction Information for ***Human*** gene ***ALDOB***

KEGG_REACTION_ID	KEGG_REACTION_NAME	KEGG_REACTION_EQN
R01068	D-fructose-1,6-bisphosphate D-glyceraldehyde-3-phosphate-lyase (glycerone-phosphate-forming)	D-Fructose 1,6-bisphosphate <=> Glycerone phosphate + D-Glyceraldehyde 3-phosphate
R01070	beta-D-fructose-1,6-bisphosphate D-glyceraldehyde-3-phosphate-lyase (glycerone-phosphate-forming)	beta-D-Fructose 1,6-bisphosphate <=> Glycerone phosphate + D-Glyceraldehyde 3-phosphate
R01829	sedoheptulose 1,7-bisphosphate D-glyceraldehyde-3-phosphate-lyase	Sedoheptulose 1,7-bisphosphate <=> Glycerone phosphate + D-Erythrose 4-phosphate
R02568	D-fructose 1-phosphate D-glyceraldehyde-3-phosphate-lyase	D-Fructose 1-phosphate <=> Glycerone phosphate + D-Glyceraldehyde

Reaction Information for ***Human*** gene ***HK1***

KEGG_REACTION_ID	KEGG_REACTION_NAME	KEGG_REACTION_EQN
R00299	ATP:D-glucose 6-phosphotransferase	ATP + D-Glucose <=> ADP + D-Glucose 6-phosphate
R00760	ATP:D-fructose 6-phosphotransferase	ATP + D-Fructose <=> ADP + D-Fructose 6-phosphate
R00867	ATP:D-fructose 6-phosphotransferase	ATP + D-Fructose <=> ADP + beta-D-Fructose 6-phosphate
R01326	ATP:D-mannose 6-phosphotransferase	ATP + D-Mannose <=> ADP + D-Mannose 6-phosphate
R01600	ATP:beta-D-glucose 6-phosphotransferase	ATP + beta-D-Glucose <=> ADP + beta-D-Glucose 6-phosphate
R01786	ATP:alpha-D-glucose 6-phosphotransferase	ATP + alpha-D-Glucose <=> ADP + alpha-D-Glucose 6-phosphate
R01961	ATP:D-glucosamine 6-phosphotransferase	ATP + D-Glucosamine <=> ADP + D-Glucosamine 6-phosphate
R03920	ATP:D-fructose 6-phosphotransferase	ATP + beta-D-Fructose <=> ADP + beta-D-Fructose 6-phosphate

TO JSON | TO CSV

Metabolites Tab   Click here to access/download;Figure;Figure6B_Main.png

Home | Genes | Pathways | Reactions | Metabolites | Studies | Summary

Metabolite Information for *Human* gene(s) *ALDOB* anatomy *Blood* disease *Diabetes*

KEGG_COMPOUND_ID	REFMET_NAME	KEGG_REACTION_ID	MET STAT_LINK
C00111	Dihydroxyacetone phosphate	R01068 R01070 R01829 R02568	
C00118	Glyceraldehyde 3-phosphate	R01068 R01070	
C00279	Erythrose 4-phosphate	R01829	
C00354	Fructose 1,6-bisphosphate	R01068	
C00447	Sedoheptulose 1,7-bisphosphate	R01829	
C00577	Glyceraldehyde	R02568	
C01094	Fructose 1-phosphate	R02568	
C05378	beta-D-Fructose 1,6-bisphosphate	R01070	

Metabolite Information for *Human* gene(s) *HK1* anatomy *Blood* disease *Diabetes*

KEGG_COMPOUND_ID	REFMET_NAME	KEGG_REACTION_ID	MET STAT_LINK
C00002	ATP	R00299 R00760 R00867 R01326 R01600 R01786 R01961 R03920	
C00008	ADP	R00299 R00760 R00867 R01326 R01600 R01786 R01961 R03920	
C00031	Glucose	R00299	
C00085	Fructose 6-phosphate	R00760	
C00092	Glucose 6-phosphate	R00299	
C00095	Fructose	R00760 R00867	
C00159	Mannose	R01326	
C00221	beta-D-Glucose	R01600	
C00267	alpha-D-Glucose	R01786	
C00275	Mannose 6-phosphate	R01326	
C00329	Glucosamine	R01961	
C00352	Glucosamine 6-phosphate	R01961	
C00668	alpha-D-Glucose 6-phosphate	R01786	
C01172	beta-D-Glucose 6-phosphate	R01600	
C02336	beta-D-Fructose	R03920	
C05345	beta-D-Fructose 6-phosphate	R00867 R03920	

TO JSON | TO CSV

Studies Tab

Click here to access/download;Figure;Figure7A_Main.png

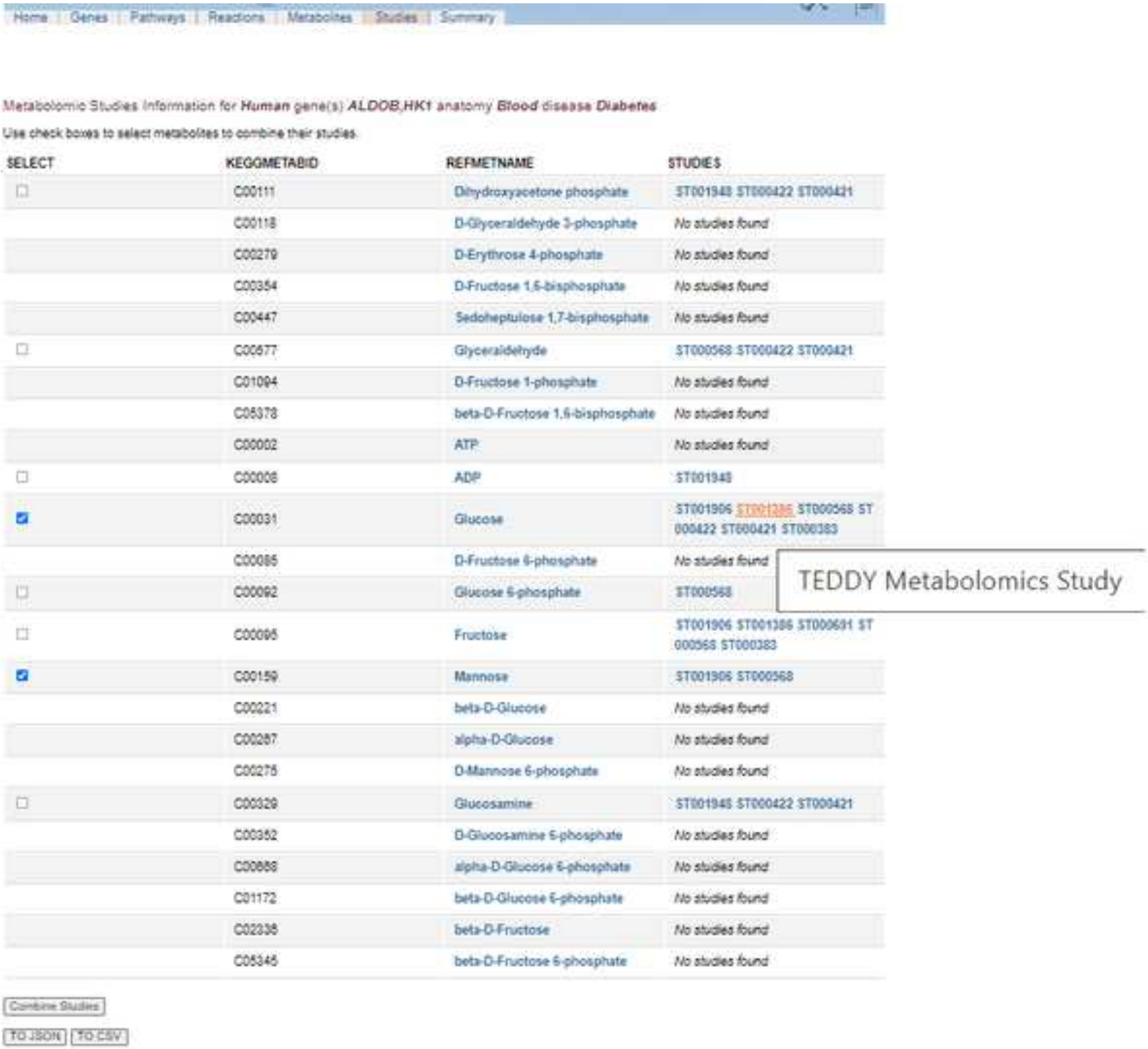

Home | Genes | Pathways | Reactions | Metabolites | Studies | Summary

Metabolomic Studies Information for **Human** gene(s) ***ALDOB,HK1*** anatomy ***Blood*** disease ***Diabetes***

Use check boxes to select metabolites to combine their studies.

SELECT	KEGGMETABID	REFMETNAME	STUDIES
☐	C00111	Dihydroxyacetone phosphate	ST001948 ST000422 ST000421
	C00118	D-Glyceraldehyde 3-phosphate	*No studies found*
	C00279	D-Erythrose 4-phosphate	*No studies found*
	C00354	D-Fructose 1,6-bisphosphate	*No studies found*
	C00447	Sedoheptulose 1,7-bisphosphate	*No studies found*
☐	C00577	Glyceraldehyde	ST000568 ST000422 ST000421
	C01094	D-Fructose 1-phosphate	*No studies found*
	C05378	beta-D-Fructose 1,6-bisphosphate	*No studies found*
	C00002	ATP	*No studies found*
☐	C00008	ADP	ST001948
☑	C00031	Glucose	ST001906 ST001386 ST000568 ST000422 ST000421 ST000383
	C00085	D-Fructose 6-phosphate	*No studies found*
☐	C00092	Glucose 6-phosphate	ST000568
☐	C00095	Fructose	ST001906 ST001386 ST000691 ST000568 ST000383
☑	C00159	Mannose	ST001906 ST000568
	C00221	beta-D-Glucose	*No studies found*
	C00267	alpha-D-Glucose	*No studies found*
	C00275	D-Mannose 6-phosphate	*No studies found*
☐	C00329	Glucosamine	ST001948 ST000422 ST000421
	C00352	D-Glucosamine 6-phosphate	*No studies found*
	C00668	alpha-D-Glucose 6-phosphate	*No studies found*
	C01172	beta-D-Glucose 6-phosphate	*No studies found*
	C02336	beta-D-Fructose	*No studies found*
	C05345	beta-D-Fructose 6-phosphate	*No studies found*

TEDDY Metabolomics Study

Combine Studies

TO JSON | TO CSV

Combined Studies View

Click here to access/download;Figure;Figure7B_Main.png

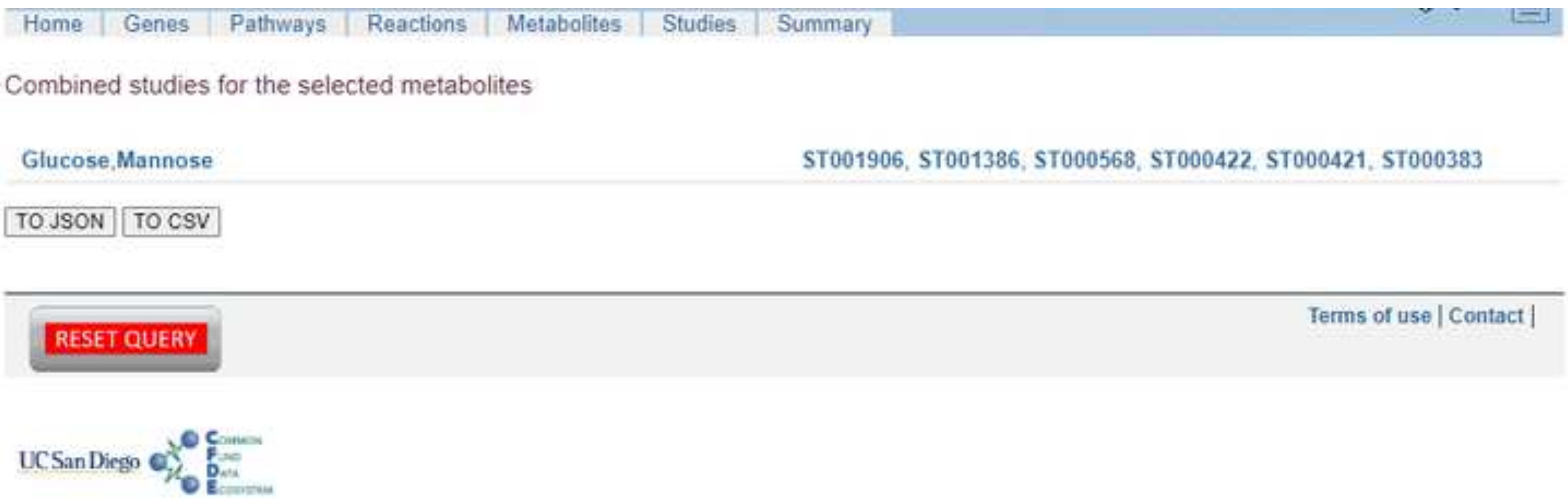

Summary Tab

Click here to access/download;Figure;Figure8_Main.png

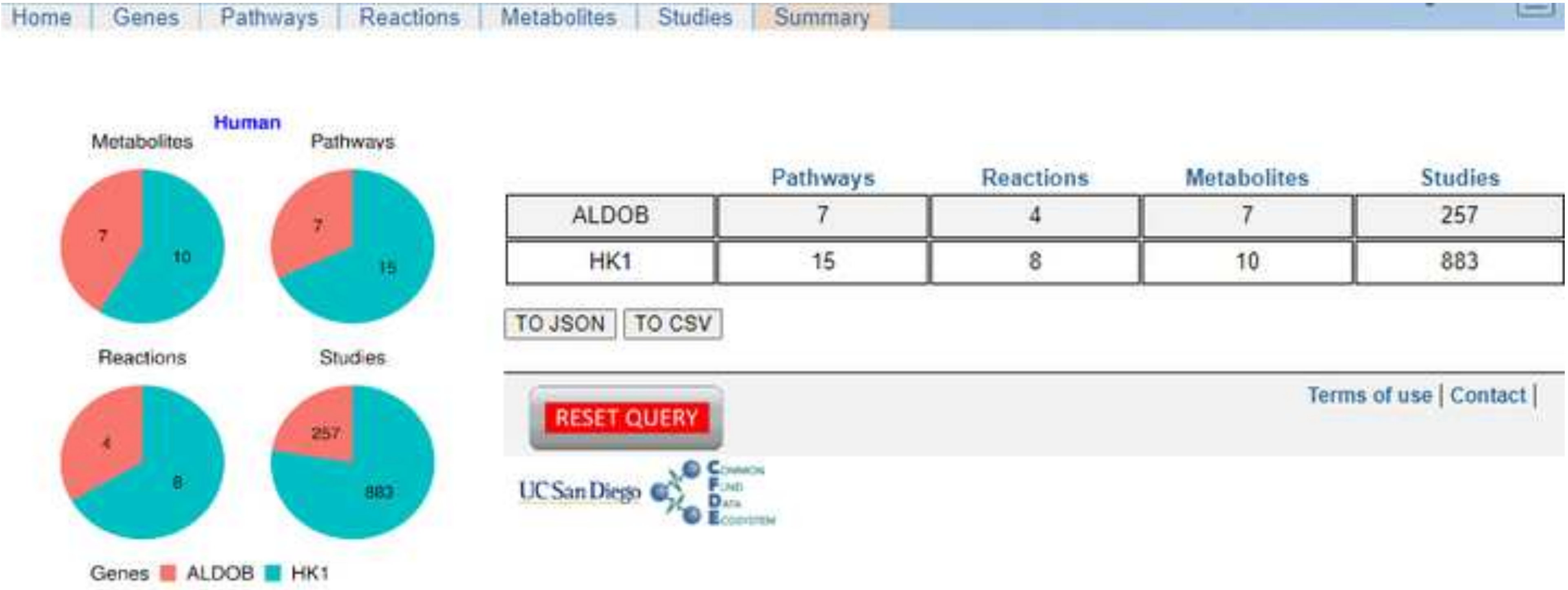

Gene Information View PNPLA3

Click here to access/download
Supplementary Material
FigureA1_Supplementary.png

Pathway Information View PNPLA3

Click here to access/download
Supplementary Material
FigureA2_Supplementary.png

Reaction and Metabolite Information PNPLA3

Click here to access/download
Supplementary Material
FigureA3_Supplementary.png

Click here to access/download
Supplementary Material
FigureA4_Supplementary.png

Information flow for query PNPLA3

Click here to access/download
Supplementary Material
FigureA5_Supplementary.png

Personal Cover
Click here to access/download;Personal Cover;FinalCoverLetter.pdf

Shankar Subramaniam
Joan and Irwin Jacobs Professor of Bioengineering & Systems Biology
Department of Bioengineering
University of California at San Diego
9500 Gilman Drive
La Jolla, CA 92093-0427
SHANKAR@UCSD.EDU
Tel: (858) 822-0986

Dr. Nicole Nogoy
Executive Editor, GigaScience

June 14, 2023

Sub: Submission of the revised manuscript to GigaScience: GIGA-D-23-00021

Dear Dr. Nogoy,

We thank you for arranging high-quality reviews. We would also like to thank the reviewers for their comments and feedback on our manuscript. We have revised the manuscript extensively to address the comments and incorporated reviewer suggestions (major edits are highlighted throughout the main manuscript). We also simplified some of the writing. We believe the manuscript reads better now and has improved considerably. We hope you will find the revised manuscript publishable. Below, we first provide a summary of the main changes followed by detailed responses to the comments by both the reviewers with page/line numbers included for clarity.

Summary of main changes:

1. We have clarified that MetGENE deals with only genes that encode for metabolic enzymes or transporters.
2. We have created a "Summary" tab in the MetGENE tool, and included some details regarding the same in the revised manuscript.
3. We have added an additional figure in the Supplementary Material (Figure A5) that depicts the information flow from query to the results for the use case of the gene PNPLA3.
4. The "Reactions" tab now includes the reaction equation.
5. We have simplified the writing as appropriate.
6. We have added some statistics regarding entity associations supported by MetGENE such as the number of gene-metabolite and gene-studies, and we have depicted them in Figure 1C.

Thank you again for the opportunity to submit our work to Gigascience. We look forward to receiving your feedback.

Sincerely,

Shankar Subramaniam

Shankar Subramaniam, Ph.D.
