# Supplementary material for: MetGENE: gene-centric metabolomics information retrieval tool
Source: Gigascience. 2023 Nov 20;12:giad089. doi: 10.1093/gigascience/giad089 (PMC10659118; doi:10.1093/gigascience/giad089)
Supplement: giad089_GIGA-D-23-00021_Revision_2 [file giad089_giga-d-23-00021_revision_2.pdf]

GigaScience

MetGENE: Gene-centric Metabolomics Information Retrieval Tool

--Manuscript Draft--

**Manuscript Number:**	GIGA-D-23-00021R2
**Full Title:**	MetGENE: Gene-centric Metabolomics Information Retrieval Tool
**Article Type:**	Research
**Funding Information:**	National Institutes of Health (U2C-DK119886) — Dr Shankar Subramaniam<br>National Institutes of Health (OT2-OD030544) — Dr Shankar Subramaniam
**Abstract:**	Background Biomedical research often involves contextual integration of multi-modal and multi-omic data in search of mechanisms for improved diagnosis, treatment and monitoring. Researchers need to access information from diverse sources, comprising data in various and sometimes incongruent formats. The downstream processing of the data to decipher mechanisms by reconstructing networks and developing quantitative models warrants considerable effort. Results MetGENE is a knowledge-based, gene-centric data aggregator that hierarchically retrieves information about the gene(s), their related pathway(s), reaction(s), metabolite(s), and metabolomic studies from standard data repositories under one dashboard to enable ease of access through centralization of relevant information. We note that MetGENE focuses only on those genes that encode for proteins directly associated with metabolites. All other gene-metabolite associations are beyond the current scope of MetGENE. Further, the information can be contextualized by filtering by species, anatomy (tissue) and condition (disease or phenotype). Conclusions MetGENE is an open-source tool that aggregates metabolite information for a given gene(s) and presents them in different computable formats, e.g., JSON, for further integration with other omics studies. MetGENE is available at https://bdcw.org/MetGENE/index.php.
**Corresponding Author:**	Shankar Subramaniam, Ph.D.<br>University of California San Diego<br>La Jolla, CA UNITED STATES
**Corresponding Author Secondary Information:**	
**Corresponding Author's Institution:**	University of California San Diego
**Corresponding Author's Secondary Institution:**	
**First Author:**	Sumana Srinivasan
**First Author Secondary Information:**	
**Order of Authors:**	Sumana Srinivasan<br>Mano Ram Maurya<br>Srinivasan Ramachandran<br>Eoin Fahy<br>Shankar Subramaniam, Ph.D.
**Order of Authors Secondary Information:**	
**Response to Reviewers:**	Reviewer 1<br><br>Reviewer: The authors have addressed my comments and suggested revisions satisfactorily.<br><br>Response: We thank the reviewer for the time and effort taken to review our revised manuscript.

Powered by Editorial Manager® and ProduXion Manager® from Aries Systems Corporation

Reviewer: One small comment - on the current entry page (https://bdcw.org/MetGENE/index.php), the "Select disease/phenotype" dropdown menu is duplicated when I access the URL (using a Chrome browser). The authors should make sure this is fixed prior to publication.

Response: This problem has been fixed. There is a dropdown menu for selecting the Disease/Phenotype category, followed by another dropdown menu (cascading filters) to select the specific Disease/Phenotype itself. This design is implemented to prevent users with an overwhelming list of diseases/phenotypes while offering them organized categories of disorders to choose from. This is mentioned in the manuscript.

Reviewer 2

Reviewer: I still insist on my comments about comparing with other similar approaches. Starting from a gene (or metabolite or proteins), a biologist can easily get a similar amount of information from MetaCyc or GeneCards. Many of those databases are already listed in the paper. In MetaCyc (and other species-specific databases), searching from genes, metabolites, and pathways is quite easy and results are curated and summarized (not as links). I am still unclear what is the additional value provided by this paper. Many of those databases/webs are already built as a summary or one-stop place for different information sources (e.g., GeneCards and MetaCyc).

Response: We thank the reviewer for the comments. While GeneCards offers comprehensive gene-related information (albeit not conveniently accessible for metabolites, even with hyperlinked ChEBI IDs), and MetaCyc furnishes insights into genes, their associated pathways, and reactions, to the best of our knowledge, no other tool effectively establishes a direct connection between a gene(s) and its pertinent metabolomic studies and datasets in the manner accomplished by MetGENE. This is important for several reasons.

We consider a scenario where a biomedical researcher aims to uncover therapeutic targets for a gene like IDH1, which plays a crucial role in the initial stages of tumorigenesis across various cancers. Given its metabolic significance, the researcher may want to find specific metabolomic studies and their findings (e.g., statistical, and biological insights) relevant to cancer. Without MetGENE, users would be compelled to manually compile a comprehensive list of metabolite names associated with a specific gene, such as IDH1, by extracting information from sources like MetaCyc. This endeavor would be arduous due to the lack of a streamlined download option for acquiring all metabolites linked to a particular gene within MetaCyc.

Taking the case of IDH1 as an example, MetaCyc designates isocitrate—a metabolite participating in the reaction catalyzed by IDH1—as D-threo-isocitrate. Converting this designation to either a KEGG ID or an INCHI key from a different database would be necessary. Subsequently, this must be input into the Metabolomics Workbench to uncover pertinent studies with relevant filters.
The resulting metabolite list could become substantial for genes such as HK1 or PNPLA3 that catalyze numerous reactions, making the entire process time-consuming, inefficient, and prone to errors. This approach would also involve managing discrepancies in metabolite nomenclature, necessitating the conversion of names from one format to another. MetGENE addresses these challenges comprehensively, eliminating the need for manual intervention and reducing the risk of errors. By automating the conversion of metabolite names to RefMet names, a format comprehensible to the Metabolomics Workbench using the MW API behind the scenes, it facilitates direct connections between gene(s) and pertinent metabolomic studies that are further contextualized by condition and anatomy. This not only drastically reduces the time and effort required by users but also streamlines the exploration of relevant data on a single, accessible platform.

Furthermore, MetGENE goes beyond offering information about individual genes; it extends its capabilities to cover entire gene sets in one seamless process. When users identify a specific subset of genes within a particular context, MetGENE stands out by efficiently processing the full list. This differs from fetching information individually, as commonly seen in platforms like GeneCards, MetaCyc, or KEGG.
Our ongoing efforts involve the integration of MetGENE APIs with NIH-CFDE's

Powered by Editorial Manager® and ProduXion Manager® from Aries Systems Corporation

	Playbook Partnership initiative, which is developing tools to build workflows by facilitating the integration of API-based decentralized tools. This integrated approach enhances efficiency and represents a significant advancement in user convenience and research capabilities. We have added this information in the introduction as a motivator for MetGENE.<br><br>Reviewer: It also seems to me that the manuscript might be providing support to MW data and a bioinformatics data workflow. If this is the case, then providing more details on the workflow and integrated usage cases might be helpful.<br><br>Response: We have extensively illustrated the utility of MetGENE through a use case involving the gene PNPLA3 in the manuscript. Furthermore, we have incorporated a scenario exemplifying the motivation behind a tool like MetGENE, focusing on the gene IDH1 and accentuating its innovative features. To augment MetGENE's functionalities, we are in the process of including additional links beyond statistics. These links will facilitate users performing metabolite enrichment on selected metabolites via MetGENE, seamlessly integrating with the Metabolomics Workbench.<br>Furthermore, we underscore the ongoing integration of MetGENE APIs into the NIH CFDE Playbook Partnership tool. This integration empowers users to conduct cross-queries across diverse CFDE resources, constructing meaningful and persistent workflows that expand their research capabilities.<br><br>Reviewer: At least, additional visualization on the web for the users and a summary table instead of links should be provided. In addition, the correctness of the link should be checked.<br><br>Response: We believe that providing additional information from other resources (in the Genes and Pathway tabs) would duplicate information and clutter the views. Instead, taking the user to the actual resource would help in utilizing the resource more effectively. We have used a tool named DeadLinkChecker (DeadLinkChecker.com) and found no dead links. To the best of our knowledge, there are no incorrect links in MetGENE.<br><br>Reviewer: The writing seems fine though it can be friendlier to laypersons by reducing the amount of abbreviation and technical details in results.<br><br>Response: We thank the reviewer for the suggestion. We have made sure we have elaborated all the abbreviations before they are used. We have tried to minimize unnecessary technical jargon in the revised manuscript.
**Additional Information:**	
**Question**	**Response**
Are you submitting this manuscript to a special series or article collection?	No
**Experimental design and statistics**<br><br>Full details of the experimental design and statistical methods used should be given in the Methods section, as detailed in our Minimum Standards Reporting Checklist. Information essential to interpreting the data presented should be made available in the figure legends.<br><br>Have you included all the information requested in your manuscript?	Yes

**Resources**<br><br>A description of all resources used, including antibodies, cell lines, animals and software tools, with enough information to allow them to be uniquely identified, should be included in the Methods section. Authors are strongly encouraged to cite Research Resource Identifiers (RRIDs) for antibodies, model organisms and tools, where possible.<br><br>Have you included the information requested as detailed in our Minimum Standards Reporting Checklist?	Yes
**Availability of data and materials**<br><br>All datasets and code on which the conclusions of the paper rely must be either included in your submission or deposited in publicly available repositories (where available and ethically appropriate), referencing such data using a unique identifier in the references and in the "Availability of Data and Materials" section of your manuscript.<br><br>Have you have met the above requirement as detailed in our Minimum Standards Reporting Checklist?	Yes

Powered by Editorial Manager® and ProduXion Manager® from Aries Systems Corporation

LaTeX - Main Document (TeX file)

Click here to access/download;LaTeX - Main Document (TeX file);main.tex

Placeholder for OUP logo oup.pdf

Placeholder for journal logo gigascience-logo.pdf

GigaScience, 2023, 1–7

doi: xx.xxxx/xxxx
Manuscript in Preparation
Paper

PAPER

MetGENE: Gene-centric Metabolomics Information Retrieval Tool

Sumana Srinivasan[1,*], Mano R. Maurya[1,*], Srinivasan Ramachandran[1], Eoin Fahy[1] and Shankar Subramaniam[1,†]

[1]Department of Bioengineering, University of California, San Diego, La Jolla, CA 92093

[†]shsubramaniam@ucsd.edu
[*]Contributed equally.

Abstract

Background Biomedical research often involves contextual integration of multi-modal and multi-omic data in search of mechanisms for improved diagnosis, treatment and monitoring. Researchers need to access information from diverse sources, comprising data in various and sometimes incongruent formats. The downstream processing of the data to decipher mechanisms by reconstructing networks and developing quantitative models warrants considerable effort. **Results** MetGENE is a knowledge-based, gene-centric data aggregator that hierarchically retrieves information about the gene(s), their related pathway(s), reaction(s), metabolite(s), and metabolomic studies from standard data repositories under one dashboard to enable ease of access through centralization of relevant information. We note that MetGENE focuses only on those genes that encode for proteins directly associated with metabolites. All other gene-metabolite associations are beyond the current scope of MetGENE. Further, the information can be contextualized by filtering by species, anatomy (tissue) and condition (disease or phenotype). **Conclusions** MetGENE is an open-source tool that aggregates metabolite information for a given gene(s) and presents them in different computable formats, e.g., JSON, for further integration with other omics studies. MetGENE is available at https://bdcw.org/MetGENE/index.php.

Key words: metabolomics workbench; gene-centric; data aggregator; web application

Introduction

Recent advances in high-throughput technologies have led to many high-resolution multiomic measurements available to biomedical researchers. However, obtaining biological insights remains challenging since considerable effort is required to find and access data from diverse sources, deal with diverse and sometimes incomplete data formats, and tease out the connections within those high-dimensional datasets. This has led to an initiative by the US National Institutes of Health (NIH) called the Common Fund Data Ecosystem (CFDE), which aims to provide a single portal that makes data findable, accessible, interoperable and reusable (FAIR) across the data repositories maintained by Data Coordination Centers (DCCs). Some examples of DCCs include the Metabolomics Workbench (MW), which is a national metabolomics data repository [1], Genotype-Tissue Expression (GTEx) Project, a comprehensive resource to study tissue-specific gene expression and regulation [2], and the Library of Integrated Network-Based Cellular Signatures (LINCS) with the goal of generating a large-scale and comprehensive catalog of perturbation-response signatures by utilizing a diverse collection of perturbations across many model systems and assay types [3]. MW is a comprehensive resource hosting more than 2000 curated metabolomics studies and provides an integrated environment for data analysis and visualization through a suite of tools and interfaces to facilitate gaining biological insights.

A gene is a fundamental unit of query in the multi-omics data hierarchy. One of the goals of CFDE is to make every DCC support gene-centric querying within their repositories. Currently, MW supports a limited capability to perform gene-centric queries on the studies. MetGENE was designed to bridge this gap and enhance the capability by allowing a user to specify a gene or a set of genes that code for the metabolic enzyme(s) as a search term

Compiled on: August 31, 2023.
Draft manuscript prepared by the author.

Key Points

- Knowledge-based data aggregator.
- Gene-centric query.
- Metabolomics Workbench studies.

and, in return, fetch the relevant information from sources like the Kyoto Encyclopedia of Genes and Genomes (KEGG) [4] and the MW. Given one or more genes, the MetGENE tool identifies associations between the gene(s) and the metabolites (biosynthesized/catabolized or transported by proteins coded by the genes) and the reactions and pathways involving these metabolites. For each metabolite, studies containing the metabolite are identified from the MW. The results are organized as a gene landing page or a Dashboard, with all the information presented in a user-friendly manner to enable further analyses. There are many other ways a gene may have an "association" with a small molecule metabolite, such as via gene regulation (e.g., TF-target relationship) or its protein product, gene expression-metabolite association, protein-protein-metabolite association, etc. However, in MetGENE, we only focus on those genes that encode for proteins directly associated with metabolites, namely, metabolic enzymes.

While other databases such as GeneCards [5] which provides extensive gene-related information (though not in a readily accessible format for metabolites, despite the presence of hyperlinked ChEBI IDs), and MetaCyc [6] which provides information on genes, their associated pathways, and reactions, to the best of our knowledge, there exists no other tool that establishes a link between a gene and its relevant metabolomic studies like MetGENE does. This is important for several reasons. Consider a scenario where a biomedical researcher aims to uncover therapeutic targets for a gene like IDH1, which plays a crucial role in the early stages of tumorigenesis across various cancers. Given its metabolic significance, the researcher may want to find specific metabolomic studies and their findings (e.g., statistical and biological insights) relevant to cancer. Currently, apart from conducting searches on the Metabolomics Workbench, the only viable option would entail painstakingly combing through scientific literature to locate relevant information. MetGENE simplifies this process by employing targeted anatomy and disease filters and seamlessly connects genes with the precise metabolomic studies of interest. This not only drastically reduces the time and effort required by users but also streamlines the exploration of relevant data on a single, accessible platform. Furthermore, MetGENE goes beyond offering information about individual genes; it extends its capabilities to cover entire gene sets in one seamless process. In scenarios where users identify a specific subset of genes within a particular context, MetGENE stands out by efficiently processing the entire list. This differs from the practice of fetching information individually, as commonly seen in platforms like GeneCards, MetaCyc, or KEGG.

Methods

MetGENE is a hierarchical, knowledge-based gene-centric information retrieval tool. Given a gene or a set of genes as a search term, MetGENE returns entities associated with the gene(s), namely pathways, reactions, metabolites and metabolomic studies in MW, as shown in Figure 1. MetGENE contextualizes the search by allowing the users to specify filters based on organism name, anatomy or tissue name (broadly, sample source), and disease/phenotype as a part of its query interface, as shown in Figure 1A. A knowledge graph represents a network of entities, such as objects or concepts, and depicts their relationship. The knowledge graph that underlies information retrieval in MetGENE is depicted in Figure 1B. Further, for

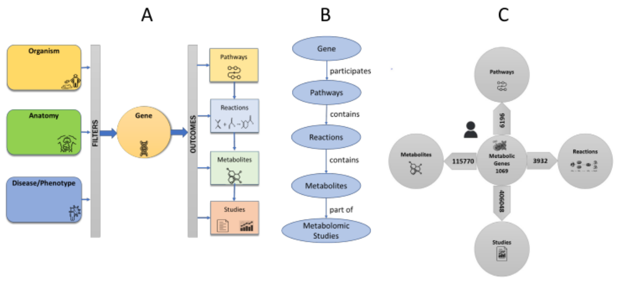

Figure 1. A. Gene(s) search is contextualized by the organism (species). The gene-associated pathways, reactions, metabolites and their corresponding metabolomic studies are reported as outcomes. Metabolites and Studies information can be filtered using anatomy (sample source), disease or phenotype. B. The knowledge graph underlying MetGENE. C. The number of associations for each relation in MetGENE.

the human species, the number of metabolic genes, gene-pathway, gene-reaction, gene-metabolite and gene-metabolomic study associations found in MW are enumerated in Figure 1C.

MetGENE is designed as a web-based application using PHP and JavaScript as the front-end. The back-end contains R scripts with wrapper functions to retrieve information from various data repositories, such as KEGG [4] (for reaction and metabolite/compound IDs) and Metabolomics Workbench (for metabolite study IDs and RefMet names), as shown in Figure 2. The KEGG database provides the KEGG REST API to access information from the KEGG database. For any given gene, MetGENE supports SYMBOL, ENTREZ ID, RefSeq, UniProt, Ensembl and ALIAS (SYMBOL_OR_ALIAS) formats and converts IDs using an in-house Gene ID Conversion Tool (GICT). The GICT uses R Bioconductor packages, org.Xy.eg.db (e.g., org.Hs.eg.db for human) and NCBI gene_info table to convert the gene IDs. If the ID type for the input term is SYMBOL_OR_ALIAS, then the term is first searched in SYMBOL. If not found, then it is searched in ALIAS. Given the three-letter KEGG organism code and the ENTREZ gene ID (of the input gene term from the GICT), the R KEGGREST API provides a way to access all the information, such as pathway IDs, reaction IDs and compound (metabolites) IDs as a data frame object which is parsed further to display relevant information. The KEGG compound ID, along with the filter information pertaining to the species, anatomy and disease/phenotype, is used to extract information such as RefMet names and Study IDs using the MW REST API. RefMet names provide a standardized reference nomenclature for both discrete metabolite structures and metabolite species identified in metabolomic experiments. This is an essential prerequisite for comparing and contrasting metabolite data across different experiments and studies. For efficiency and to speed up the display, MetGENE caches the number of pathways, reactions, metabolites and studies associated with a particular gene which is updated weekly to accommodate new studies being deposited into MW.

MetGENE maintains session variables for species ID and organism name; ENTREZ gene ID and gene symbol; anatomy, disease/phenotype terms, and the previous values of these terms to enable server-side caching of pages and thus avoid unnecessary and time-consuming fetching of data across the network. The MetGENE back-end R functions are packaged into a library called metgene and will be available on GitHub for download. For pro-

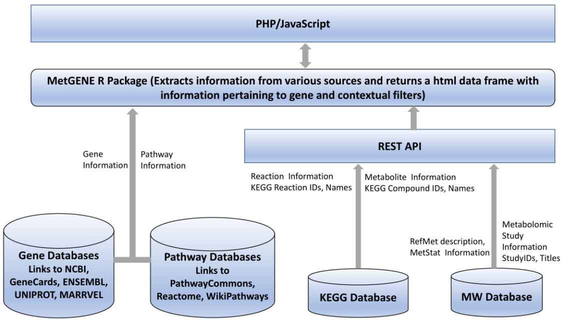

Figure 2. The Architecture of MetGENE comprises server-side PHP and JavaScript interacting with R scripts that use REST APIs to extract information from KEGG and MW databases. The gene and pathway information links are generated for specific repositories.

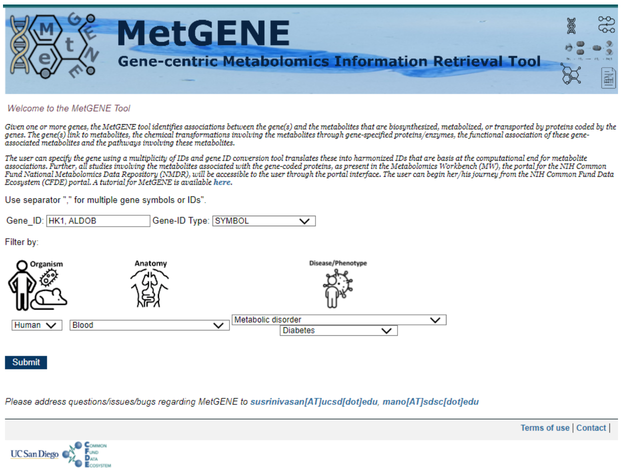

Figure 3. MetGENE Query page with the organism, anatomy (sample source), disease/phenotype-specific filters.

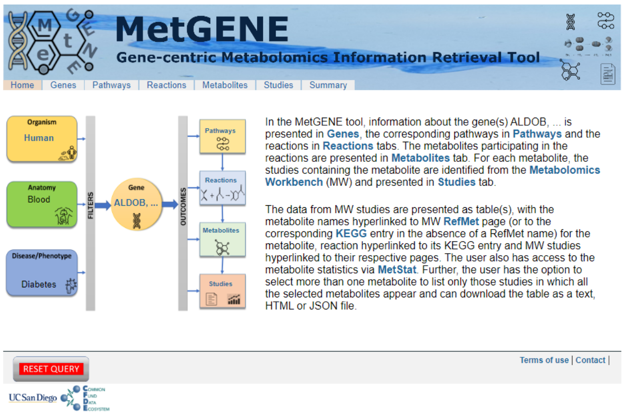

Figure 4. MetGENE landing page with context-sensitive display and access to Gene, Pathway, Reactions, Metabolites, Study and Summary information.

grammatic ease of access, we provide REST APIs that output each information table displayed in JSON or CSV formats. The REST APIs are developed using Smart/Open API format [7].

Results

In this section, we describe the user experience starting from the MetGENE Query Page and ending with MetGENE Studies Page containing metabolomic studies corresponding to the gene in the Metabolomics Workbench, incorporating various intermediate views of interest based on the knowledge graph described earlier.

MetGENE Query Interface

The user can input the gene information as a gene ID in any one of the formats described in the previous section. The format of the query page is as shown in Figure 3. The gene search input is validated on the client-side to allow only alphanumeric symbols. Invalid gene IDs are recognized, and appropriate error messages are displayed. MetGENE uses terms (e.g., Human, Mouse) for taxonomy filtering as per the NCBI taxonomy database (Coordinators, 2000). Currently, MetGENE supports human (*H. sapiens*), mouse (*M. musculus*) and rat (*R. norvegicus*) species, and we plan to add *E. coli* (K12), *C. elegans*, common fruit fly (*D. melanogaster*), and mosquito (*A. gambiae*) in the near future. For filtering the information on metabolites and studies by anatomy/tissue (e.g., Liver, Blood) and disease/phenotype (e.g., Diabetes, Fatty liver disease), terms from Metabolomics Workbench are used. Internally, the MW database records disease and phenotype under the metadata field, *disease*. Hence, the phenotype is searched as a disease term within MW. The JSON files for each filter/category are curated and updated regularly and used to generate a pull-down menu. For the disease/phenotype filter, a two-step selection menu with slim (or disease class) terms in the first level and fine-grained terms in the second level is used for ease of presenting the options to the user. The user inputs from this page (main landing page) are submitted as a form, and a second page for MetGENE (as shown in Figure 4) is populated with the context-specific filtering terms. The second page comprises tabs for the search term associated entities, "Genes", "Pathways", "Reactions", "Metabolites", "Studies" and "Summary". The Summary tab displays the total number of pathways, reactions, metabolites and studies corresponding to each gene in the query. As MetGENE supports only those genes that encode proteins directly associated with metabolites, a warning is issued if the queried gene or a set of queried genes does not encode for such proteins.

Gene and Pathway Information Pages

The gene information page shown in Figure 5a presents gene IDs in different formats hyperlinked to the corresponding web pages pointing to repositories such as KEGG [4], GeneCards [5], NCBI [8], Ensembl [9], UniProt [10] and Marrvel [11]. The URLs to these repositories for the specific genes are constructed based on their base URLs and the respective supported gene ID types. This information is obtained from the REST API of the GICT and converted from JSON to a HTML table format for display purposes.

The pathway information page (Figure 5b) displays gene symbols hyperlinked with species and gene ID or symbol information as appropriate to various well-maintained pathway databases such as Pathway Commons [12], Reactome [13], KEGG [4] and Wikipathways [14]. MetGENE provides context-specific ease of access to these online resources.

Reaction and Metabolite Information Pages

The KEGG database provides the KEGG REST API to access information from the KEGG database. Given the organism code and the gene ID, the R KEGGREST API provides a way to access all the information, such as pathway IDs, reaction IDs, reaction names, reaction equations and compound (metabolites) IDs which are displayed in a tabular format. Figure 6a depicts the reaction information tab corresponding to the metabolic gene(s) of interest in a table view (one per gene) comprising reaction IDs hyperlinked to the corresponding KEGG reaction information page, reaction names and the

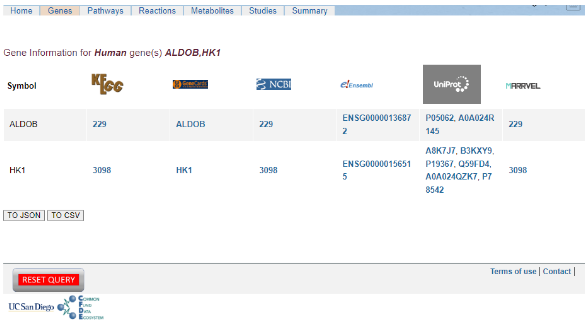

(a) Genes tab view.

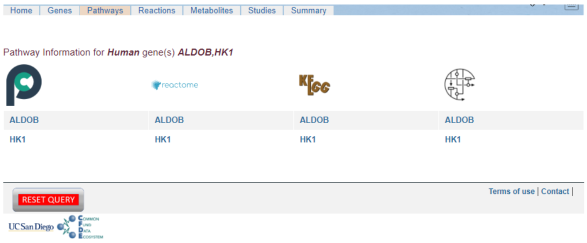

(b) Pathways tab view.

Figure 5. (a) MetGENE gene information page comprising Gene IDs in various formats corresponding to the searched gene(s) hyperlinked to various online resources. (b) MetGENE pathway information page comprising Gene IDs hyperlinked to various pathway resources.

reaction equation.

In the metabolites information tab, as shown in Figure 6b, a unique list of metabolites across all reactions corresponding to a given gene, along with their respective RefMet names (MW provides REST APIs to access the RefMet name corresponding to a KEGG compound ID), KEGG reaction IDs of all the reactions the metabolite participates in, are displayed. Further, the MW MetStat link provides access to the statistics about the metabolite measured across various studies in the MW database, filtered by anatomy (sample source) and disease/phenotype. This tool generates a report for any given metabolite in MW, comprising all the unique studies containing that metabolite and their median value of the relative standard deviation (RSD) across all those studies. The KEGG compound IDs that do not have RefMet names in the MW database display only the KEGG compound name and reaction IDs. The KEGG compound IDs are hyperlinked to the corresponding KEGG compound information page. MetGENE allows users to download the tables directly from the displayed page in JSON or CSV formats for further analysis. MW also provides REST APIs to access all the study IDs, titles, and RefMet names for a given KEGG compound ID in JSON, text and HTML formats.

MetGENE Metabolomics Study Information Page

In the Studies page (as shown in Figure 7a), a tabular view of the unique list of metabolites for the queried gene(s), their RefMet names hyperlinked to their corresponding description page in MW, and a comma-separated list of study IDs in which the metabolite participates (with each study ID linked to its corresponding study description page in MW) is presented. Further, a helpful text hover feature displaying the study title corresponding to a particular study ID is also provided to the user. MetGENE also allows users to select metabolites of interest and combine their studies for download and further analysis, as shown in Figure 7b.

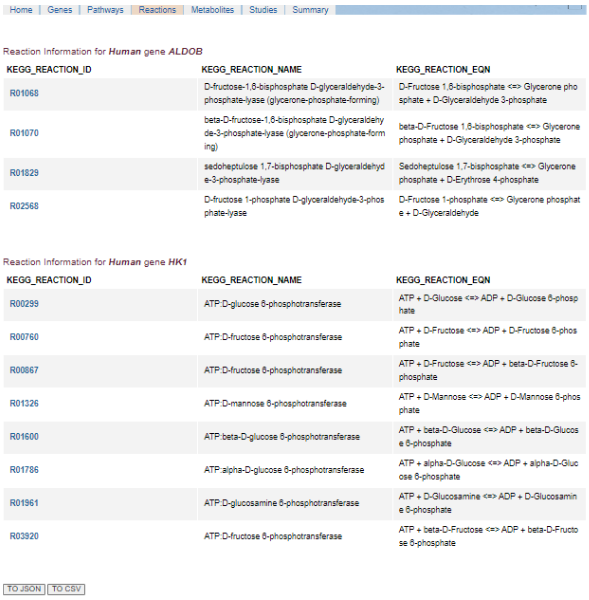

(a) Reactions tab view.

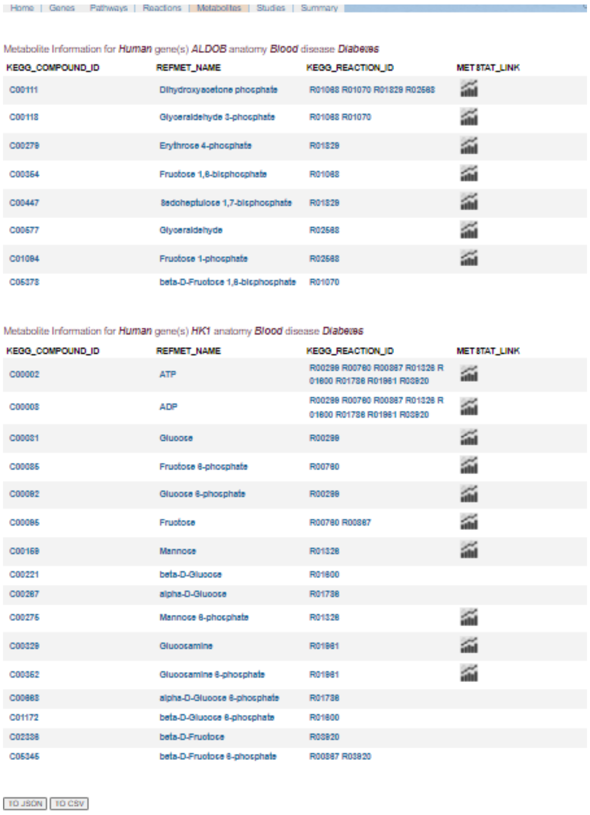

(b) Metabolites tab view.

Figure 6. (a) MetGENE reaction information page comprising KEGG Reaction IDs hyperlinked to KEGG reaction information page and reaction descriptions in a tabular format. (b) MetGENE metabolite information page comprises KEGG compound IDs, MW RefMet names, reaction IDs in which a metabolite participates in, hyperlinked to the KEGG reaction information page and MetStat link for the metabolite in a tabular format.

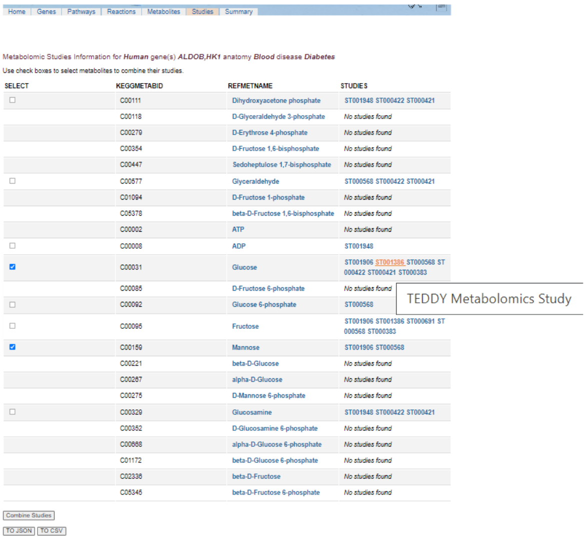

(a) Studies tab view.

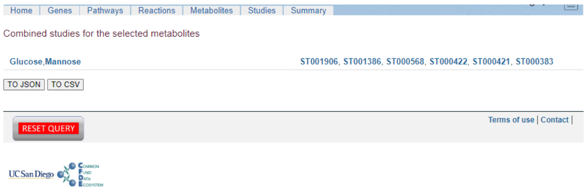

(b) Combined studies tab view.

Figure 7. (a) MetGENE metabolomics studies information page comprises KEGG compound IDs, RefMet names, and MW study IDs corresponding to a metabolite in a tabular format. (b) MetGENE allows users to combine studies for a selected set of metabolites.

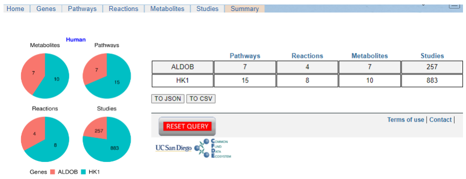

Figure 8. The Summary tab in MetGENE enumerates the total number of pathways, reactions, metabolites and metabolomic studies corresponding to each queried gene.

MetGENE Summary Page

In the Summary page (as shown in Figure 8), MetGENE displays the total number of pathways, reactions, metabolites and metabolomic studies for each queried gene and displays the information in a tabular format and a pie chart. This information is available for download both in JSON and CSV formats.

Case Study: Exploring gene PNPLA3 using MetGENE

Here, we demonstrate a use case that shows the utility of MetGENE as a one-stop tool to obtain all metabolomic information associated with a gene(s) in a specific disease condition. The protein Adiponutrin, encoded by the gene PNPLA3, is a multi-functional enzyme that belongs to the IPLA2/lipase family, which has both triacylglycerol lipase and acylglycerol O-acyltransferase activities. PNPLA3 is predominantly expressed in adipocytes and liver cells. It regulates the development of adipocytes and the metabolism of fats (lipogenesis and lipolysis). Diseases associated with PNPLA3 mutations include Fatty Liver Disease and Non-Alcoholic Steatohepatitis (NASH) [15],[16], [17] and [18].

To obtain information about a gene(s) and associated entities like enzymes, reactions, pathways and existing metabolomic studies in MW for a given context (fatty liver disease in humans), a user needs to identify precise key terms, perform a search on the internet, sift through the results to identify literature about various metabolomic studies and download the studies to perform downstream analyses. These steps are sometimes time-consuming and misleading, depending on the search terms' specificity. However, with MetGENE, the user can specify a gene ID(s) in any popular format and apply the filters on organism, anatomy, disease/phenotype to obtain genes, pathways, reactions, metabolites and studies information consolidated from various data resources, at one go. Supplementary Figure A1 shows the Gene tab for the PNLPA3 Gene. Links to various online resources (GeneCards, KEGG, NCBI, Uniprot and Marrvel) with genomic, transcriptomic, proteomic, clinical and functional information (via GeneCards link), module and pathway information (via KEGG), transcript and region information (via NCBI link), sequence, variant, gene expression information (via Ensembl link), protein sequence and functional information (via Uniprot link) and gene variants associated with PNLPA3 (via Marrvel link) are provided in the gene information table. Supplementary Figure A2 represents the Pathway tab for the PNLPA3 gene with links to interactions with and pathways that involve PNPLA3 (via Pathway Commons), protein and reaction location information (via Reactome), pathway definitions (via KEGG), and pathway-related collaborative information (via Wikipathways). Supplementary Figure A3 represents the Reactions and Metabolites tabs for PNPLA3. It delineates two hydrolysis reactions where diacylglycerol (DAG) is hydrolyzed to 2-monoacylglycerol (2-MAG), and triacylglycerol (TAG) is hydrolyzed to DAG by PNPLA3. Three generic compounds (TAG, DAG, and Fatty acid) participate in the reactions. The Metabolites tab for PNPLA3 lists all the metabolites (with substitutions for TAG and DAG) along with corresponding RefMet names or KEGG metabolite names (in the absence of RefMet names) along with a MetStat link that points to metabolite statistics information such as a histogram of the RSD (Relative Standard Deviation) metabolite data, ANOVA results for the metabolite with a cut-off p-value in MW via MetStat, with the anatomy and disease filters applied. Supplementary Figure A4 depicts the Studies tab in MetGENE for the gene PNPLA3. Each metabolite would display the corresponding Study IDs if measured/listed in any studies deposited in MW, hyperlinked to the study description. A hover text displays the study title. The tool provides the user with the ability to combine studies for metabolites of interest to a consolidated view, as shown in Supplementary Figure A4. Supplementary Figure A5 serves as an informative visual aid, providing an overview of the information flow network within MetGENE for the PNPLA3 use case. It simplifies the complexity of the underlying technical details of MetGENE, facilitating comprehension of the query-to-results process. All of MetGENE tables can be downloaded in formats such as JSON and CSV directly from the respective pages in the browser or via the REST API. The REST API supports JSON and text formats. They are deposited in the Smart API repository along with the accompanying documentation.

Discussion

Given a gene(s), the MetGENE tool identifies associations between the gene(s) and the metabolites that are biosynthesized, catabolized, or transported by proteins coded by those gene(s). It is a knowledge-based data aggregator, accessing and integrating data from resources such as the KEGG and Metabolomics Workbench. The gene(s) link to metabolites, the chemical transformations involving the metabolites through gene-specified proteins/enzymes, the functional association of these gene-associated metabolites and the pathways involving these metabolites with context-based filtering based on anatomy (sample source), disease/phenotype. The user can specify the gene using a multiplicity of IDs, and the gene

ID conversion tool translates these into harmonized IDs that are the basis for metabolite associations. Further, all studies involving the metabolites associated with the gene-coded proteins, as present in the Metabolomics Workbench (MW), will be accessible to the user as a stand-alone tool or via the portal interface for the NIH Common Fund National Metabolomics Data Repository (NMDR). The user can begin their journey either from the main web page for MetGENE (see Availability) or from the NIH Common Fund Data Ecosystem (CFDE) portal (https://app.nih-cfde.org/; the steps are: Data Browser → Vocabulary → Gene).

Potential implications

Features from MetGENE will contribute to the integration of other omics data to draw metabolomics perspectives and with metabolomics data, with genes serving as the bridging nodes. For example, tools such as MetGENE will assist a researcher in interpreting the results of multi-omics data integration holistically, where they can consider both gene-related and metabolomics data in a metabolic pathway. We are also in the process of integrating MetGENE with tools from other DCCs as a part of a broader NIH-CFDE initiative. Through that, we can provide support for persistent and shareable MetGENE-based workflows.

Availability of Supporting Code and Requirements

MetGENE is an open-source collaborative initiative available at GitHub. The main website of MetGENE and the Smart APIs are available, as shown below. MetGENE is registered with the SciCrunch registry (RRID:SCR_023402 (https://scicrunch.org)). We also plan to register MetGENE-based workflows as BioCompute Objects (BCOs) in the Galaxy Community Hub (https://galaxyproject.org/) in the near future as a part of NIH-CFDE efforts.

- Project Name: MetGENE
- Project Home Page: https://bdcw.org/MetGENE/index.php
- Operating System: Platform independent
- Programming languages: R and PHP
- Smart APIs: https://smart-api.info/registry/?q=MetGENE
- Source code: https://github.com/metabolomicsworkbench/MetGENE

Additional Files

- Supplementary Figure A1: The gene information tab for PNPLA3 in MetGENE has links to various online resources that provide different types of information pertaining to the gene, including sequence, gene expression, protein structure, functional and disease information.
- Supplementary Figure A2: The pathway information tab for PNPLA3 in MetGENE has links to various online resources that provide different types of information pertaining to the pathways in which PNPLA3 participates.
- Supplementary Figure A3: The reaction and metabolite information tabs for PNPLA3 in MetGENE have links to reactions controlled by PNLPA3, with Reaction IDs linked to the KeggKEGG reaction definition page. RefMet provides the metabolite details, and the MetStat link provides the context-specific metabolite measurement statistics.
- Supplementary Figure A4: The studies information tab for PNPLA3 in MetGENE has links to studies in MW pertaining to the metabolites listed. Studies can be combined for selected metabolites for further analysis.
- Supplementary Figure A5: A consolidated network of information flow for gene PNPLA3 demonstrating how pathways, reactions, metabolites, studies and summary are connected in the result.

Declarations

List of abbreviations

- CFDE - Common Fund Data Ecosystem
- DCC - Data Coordination Centers
- GTEx - Gene Tissue Expression
- LINCS - Library of Integrated Network-Based Cellular Signatures
- GICT - Gene ID Conversion tool
- MW - Metabolomics Workbench
- NMDR - National Metabolomics Data Repository

Ethical Approval

Not Applicable.

Consent for publication

Not Applicable.

Competing Interests

Not Applicable.

Funding

This work has been supported by the National Institutes of Health Grants (Metabolomics Workbench, U2C-DK119886 and Common Fund Data Ecosystem (CFDE) OT2-OD030544).

Author's Contributions

This work was conceptualized by Sh.S., Su.S., M.R.M. and S.R. The funding acquisition was by Sh.S. The methodology, software development and visualization for MetGENE were developed by Su.S. and M.R.M. Validation and testing was performed by Su.S., M.R.M., S.R. and Sh.S. The disease ontology terms and structuring was provided by S.R. The Metabolomics Workbench REST APIs that are used in this work were developed by E.F. The original draft for the paper was prepared by Su.S and reviewing and editing was done by Sh.S, M.R.M. and S.R. The project administration was done by Sh.S and M.R.M.

Acknowledgements

We thank the Common Fund Data Ecosystem Gene Working Group for useful discussions.

References

1. Sud M, Fahy E, Cotter D, Azam K, Vadivelu I, Burant C, et al. Metabolomics Workbench: An international repository for metabolomics data, metadata and metabolite standards, protocols, tutorials and training, and analysis tools. Nucleic acids research 2016;44:D463–70. http://www.metabolomicsworkbench.org.
2. GTExConsortium. Human genomics. The Genotype-Tissue Expression (GTEx) pilot analysis: multitissue gene regula-

tion in humans. Science 2015;348(6235):648–660. https://gtexportal.org/home/.
3. V S, J T, A K, D V, D C, M FN, et al. LINCS Data Portal 2.0: next generation access point for perturbation-response signatures. Nucleic Acids Research 2020;48:D431–D439.
4. Kanehisa M, Goto S. KEGG: kyoto encyclopedia of genes and genomes. Nucleic Acids Research 2000;28(1):27–30. https://www.genome.jp/kegg/kegg1.html.
5. Safran M, Rosen N, Twik M, BarShir R, Stein TI, Dahary D, et al. In: The GeneCards Suite Singapore: Springer Singapore; 2021. p. 27–56. https://doi.org/10.1007/978-981-16-5812-9_2.
6. R C, R B, IM K, A K, M K, PE M, et al. The MetaCyc database of metabolic pathways and enzymes - a 2019 update. Nucleic Acids Research 2020;48:D445–D453.
7. Schloerke B, Allen J. plumber: An API Generator for R; 2022, https://www.rplumber.io, https://github.com/rstudio/plumber.
8. S F. The NCBI Taxonomy database. Nucleic acids research. Nucleic Acids Research 2012;40:D136–D143. https://doi.org/10.1093/nar/gkr1178.
9. Cunningham F, Allen JE, Allen J, Alvarez-Jarreta J, Amode MR, Armean IM, et al. Ensembl 2022. Nucleic Acids Research 2022;50(1):D988–D995. https://doi.org/10.1093/nar/gkab1049.
10. Consortium TU. UniProt: the universal protein knowledgebase in 2021. Nucleic Acids Research 2021;49(D1):D480–D489. https://doi.org/10.1093/nar/gkaa1100.
11. Wang J, Al-Ouran, R, Hu, Y, Kim, et al. MARRVEL: Integration of Human and Model Organism Genetic Resources to Facilitate Functional Annotation of the Human Genome. American journal of human genetics 2021;100(6):843–853. https://doi.org/10.1016/j.ajhg.2017.04.010.
12. Cerami EG, Gross BE, Demir E, Rodchenkov I, Babur O, Anwar N, et al. Pathway Commons, a web resource for biological pathway data. Nucleic Acids Research 2010;39:D685–D690. https://www.pathwaycommons.org/.
13. Gillespie M, Jassal B, Stephan R, Milacic M, Rothfels K, Senff-Ribeiro A, et al. The reactome pathway knowledgebase 2022. Nucleic Acids Research 2021;50(D1):D687–D692. https://reactome.org/.
14. M M, A A, A R, A W, DN S, K H, et al. WikiPathways: connecting communities. Nucleic Acids Research 2021;49:D613–D621. https://doi.org/10.1093/nar/gkaa1024.
15. Cohen J, Horton J, HH H. Human fatty liver disease: old questions and new insights. Science 2011;332(6037):1519–23.
16. Gorden D, Myers D, Ivanova P, Fahy E, Maurya M, Gupta S, et al. Human fatty liver disease: old questions and new insights. Science 2011;332(6037):1519–23.
17. P P, S R. The role of PNPLA3 in health and disease. Biochem Biophys Acta Mol Cell Biol Lipids 2019;1864(6):900–906.
18. XC D. PNPLA3—A Potential Therapeutic Target for Personalized Treatment of Chronic Liver Disease. Front Med 2019;6(304).

Manuscript Click here to access/download;Manuscript;Manuscript.pdf.pdf

OXFORD (GIGA)^n SCIENCE

GigaScience, 2023, 1–7

doi: xx.xxxx/xxxx
Manuscript in Preparation
Paper

PAPER

MetGENE: Gene-centric Metabolomics Information Retrieval Tool

Sumana Srinivasan[1,*], Mano R. Maurya[1,*], Srinivasan Ramachandran[1], Eoin Fahy[1] and Shankar Subramaniam[1,†]

[1]Department of Bioengineering, University of California, San Diego, La Jolla, CA 92093

[†]shsubramaniam@ucsd.edu
[*]Contributed equally.

Abstract

Background Biomedical research often involves contextual integration of multi-modal and multi-omic data in search of mechanisms for improved diagnosis, treatment and monitoring. Researchers need to access information from diverse sources, comprising data in various and sometimes incongruent formats. The downstream processing of the data to decipher mechanisms by reconstructing networks and developing quantitative models warrants considerable effort. **Results** MetGENE is a knowledge-based, gene-centric data aggregator that hierarchically retrieves information about the gene(s), their related pathway(s), reaction(s), metabolite(s), and metabolomic studies from standard data repositories under one dashboard to enable ease of access through centralization of relevant information. We note that MetGENE focuses only on those genes that encode for proteins directly associated with metabolites. All other gene-metabolite associations are beyond the current scope of MetGENE. Further, the information can be contextualized by filtering by species, anatomy (tissue) and condition (disease or phenotype). **Conclusions** MetGENE is an open-source tool that aggregates metabolite information for a given gene(s) and presents them in different computable formats, e.g., JSON, for further integration with other omics studies. MetGENE is available at `https://bdcw.org/MetGENE/index.php`.

Key words: metabolomics workbench; gene-centric; data aggregator; web application

Introduction

Recent advances in high-throughput technologies have led to many high-resolution multiomic measurements available to biomedical researchers. However, obtaining biological insights remains challenging since considerable effort is required to find and access data from diverse sources, deal with diverse and sometimes incomplete data formats, and tease out the connections within those high-dimensional datasets. This has led to an initiative by the US National Institutes of Health (NIH) called the Common Fund Data Ecosystem (CFDE), which aims to provide a single portal that makes data findable, accessible, interoperable and reusable (FAIR) across the data repositories maintained by Data Coordination Centers (DCCs). Some examples of DCCs include the Metabolomics Workbench (MW), which is a national metabolomics data repository [1], Genotype-Tissue Expression (GTEx) Project, a comprehensive resource to study tissue-specific gene expression and regulation [2], and the Library of Integrated Network-Based Cellular Signatures (LINCS) with the goal of generating a large-scale and comprehensive catalog of perturbation-response signatures by utilizing a diverse collection of perturbations across many model systems and assay types [3]. MW is a comprehensive resource hosting more than 2000 curated metabolomics studies and provides an integrated environment for data analysis and visualization through a suite of tools and interfaces to facilitate gaining biological insights.

A gene is a fundamental unit of query in the multi-omics data hierarchy. One of the goals of CFDE is to make every DCC support gene-centric querying within their repositories. Currently, MW supports a limited capability to perform gene-centric queries on the studies. MetGENE was designed to bridge this gap and enhance the capability by allowing a user to specify a gene or a set of genes that code for the metabolic enzyme(s) as a search term

Compiled on: September 1, 2023.
Draft manuscript prepared by the author.

Key Points

- Knowledge-based data aggregator.
- Gene-centric query.
- Metabolomics Workbench studies.

and, in return, fetch the relevant information from sources like the Kyoto Encyclopedia of Genes and Genomes (KEGG) [4] and the MW. Given one or more genes, the MetGENE tool identifies associations between the gene(s) and the metabolites (biosynthesized/catabolized or transported by proteins coded by the genes) and the reactions and pathways involving these metabolites. For each metabolite, studies containing the metabolite are identified from the MW. The results are organized as a gene landing page or a Dashboard, with all the information presented in a user-friendly manner to enable further analyses. There are many other ways a gene may have an "association" with a small molecule metabolite, such as via gene regulation (e.g., TF-target relationship) or its protein product, gene expression-metabolite association, protein-protein-metabolite association, etc. However, in MetGENE, we only focus on those genes that encode for proteins directly associated with metabolites, namely, metabolic enzymes.

While other databases such as GeneCards [5] which provides extensive gene-related information (though not in a readily accessible format for metabolites, despite the presence of hyperlinked ChEBI IDs), and MetaCyc [6] which provides information on genes, their associated pathways, and reactions, to the best of our knowledge, there exists no other tool that establishes a link between a gene and its relevant metabolomic studies like MetGENE does. This is important for several reasons. Consider a scenario where a biomedical researcher aims to uncover therapeutic targets for a gene like IDH1, which plays a crucial role in the early stages of tumorigenesis across various cancers. Given its metabolic significance, the researcher may want to find specific metabolomic studies and their findings (e.g., statistical and biological insights) relevant to cancer. Currently, apart from conducting searches on the Metabolomics Workbench, the only viable option would entail painstakingly combing through scientific literature to locate relevant information. MetGENE simplifies this process by employing targeted anatomy and disease filters and seamlessly connects genes with the precise metabolomic studies of interest. This not only drastically reduces the time and effort required by users but also streamlines the exploration of relevant data on a single, accessible platform. Furthermore, MetGENE goes beyond offering information about individual genes; it extends its capabilities to cover entire gene sets in one seamless process. In scenarios where users identify a specific subset of genes within a particular context, MetGENE stands out by efficiently processing the entire list. This differs from the practice of fetching information individually, as commonly seen in platforms like GeneCards, MetaCyc, or KEGG.

Methods

MetGENE is a hierarchical, knowledge-based gene-centric information retrieval tool. Given a gene or a set of genes as a search term, MetGENE returns entities associated with the gene(s), namely pathways, reactions, metabolites and metabolomic studies in MW, as shown in Figure 1. MetGENE contextualizes the search by allowing the users to specify filters based on organism name, anatomy or tissue name (broadly, sample source), and disease/phenotype as a part of its query interface, as shown in Figure 1A. A knowledge graph represents a network of entities, such as objects or concepts, and depicts their relationship. The knowledge graph that underlies information retrieval in MetGENE is depicted in Figure 1B. Further, for

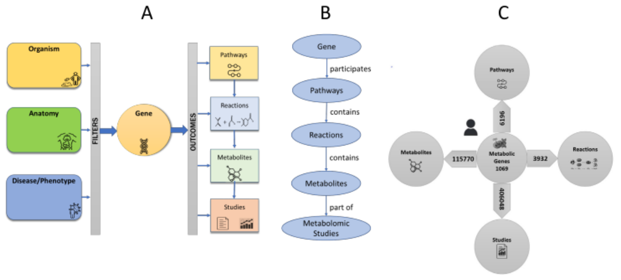

Figure 1. A. Gene(s) search is contextualized by the organism (species). The gene-associated pathways, reactions, metabolites and their corresponding metabolomic studies are reported as outcomes. Metabolites and Studies information can be filtered using anatomy (sample source), disease or phenotype. B. The knowledge graph underlying MetGENE. C. The number of associations for each relation in MetGENE.

the human species, the number of metabolic genes, gene-pathway, gene-reaction, gene-metabolite and gene-metabolomic study associations found in MW are enumerated in Figure 1C.

MetGENE is designed as a web-based application using PHP and JavaScript as the front-end. The back-end contains R scripts with wrapper functions to retrieve information from various data repositories, such as KEGG [4] (for reaction and metabolite/compound IDs) and Metabolomics Workbench (for metabolite study IDs and RefMet names), as shown in Figure 2. The KEGG database provides the KEGG REST API to access information from the KEGG database. For any given gene, MetGENE supports SYMBOL, ENTREZ ID, RefSeq, UniProt, Ensembl and ALIAS (SYMBOL_OR_ALIAS) formats and converts IDs using an in-house Gene ID Conversion Tool (GICT). The GICT uses R Bioconductor packages, org.Xy.eg.db (e.g., org.Hs.eg.db for human) and NCBI gene_info table to convert the gene IDs. If the ID type for the input term is SYMBOL_OR_ALIAS, then the term is first searched in SYMBOL. If not found, then it is searched in ALIAS. Given the three-letter KEGG organism code and the ENTREZ gene ID (of the input gene term from the GICT), the R KEGGREST API provides a way to access all the information, such as pathway IDs, reaction IDs and compound (metabolites) IDs as a data frame object which is parsed further to display relevant information. The KEGG compound ID, along with the filter information pertaining to the species, anatomy and disease/phenotype, is used to extract information such as RefMet names and Study IDs using the MW REST API. RefMet names provide a standardized reference nomenclature for both discrete metabolite structures and metabolite species identified in metabolomic experiments. This is an essential prerequisite for comparing and contrasting metabolite data across different experiments and studies. For efficiency and to speed up the display, MetGENE caches the number of pathways, reactions, metabolites and studies associated with a particular gene which is updated weekly to accommodate new studies being deposited into MW.

MetGENE maintains session variables for species ID and organism name; ENTREZ gene ID and gene symbol; anatomy, disease/phenotype terms, and the previous values of these terms to enable server-side caching of pages and thus avoid unnecessary and time-consuming fetching of data across the network. The MetGENE back-end R functions are packaged into a library called metgene and will be available on GitHub for download. For pro-

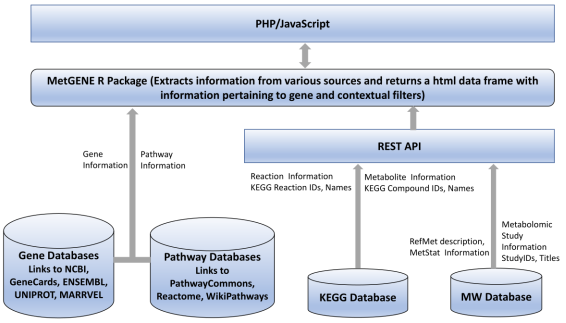

Figure 2. The Architecture of MetGENE comprises server-side PHP and JavaScript interacting with R scripts that use REST APIs to extract information from KEGG and MW databases. The gene and pathway information links are generated for specific repositories.

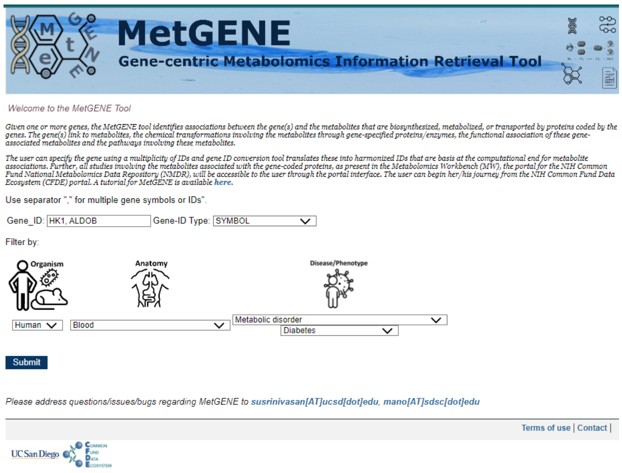

Figure 3. MetGENE Query page with the organism, anatomy (sample source), disease/phenotype-specific filters.

grammatic ease of access, we provide REST APIs that output each information table displayed in JSON or CSV formats. The REST APIs are developed using Smart/Open API format [7].

Results

In this section, we describe the user experience starting from the MetGENE Query Page and ending with MetGENE Studies Page containing metabolomic studies corresponding to the gene in the Metabolomics Workbench, incorporating various intermediate views of interest based on the knowledge graph described earlier.

MetGENE Query Interface

The user can input the gene information as a gene ID in any one of the formats described in the previous section. The format of the query page is as shown in Figure 3. The gene search input is validated on the client-side to allow only alphanumeric symbols. Invalid gene IDs are recognized, and appropriate error messages are displayed. MetGENE uses terms (e.g., Human, Mouse) for taxonomy filtering as per the NCBI taxonomy database (Coordinators, 2000). Currently, MetGENE supports human (*H. sapiens*), mouse (*M. musculus*) and rat (*R. norvegicus*) species, and we plan to add *E. coli* (K12), *C. elegans*, common fruit fly (*D. melanogaster*), and mosquito (*A. gambiae*) in the near future. For filtering the information on metabolites and studies by anatomy/tissue (e.g., Liver, Blood) and disease/phenotype (e.g., Diabetes, Fatty liver disease), terms from

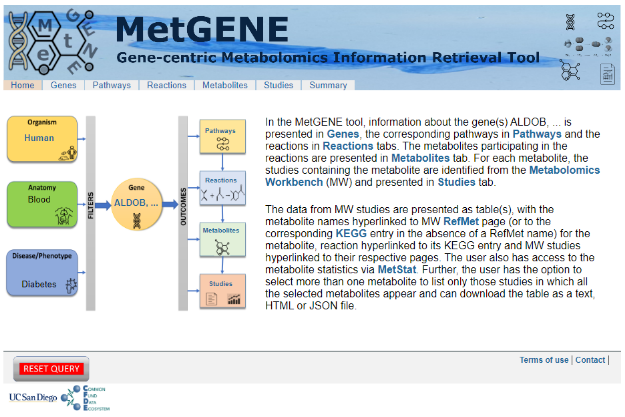

Figure 4. MetGENE landing page with context-sensitive display and access to Gene, Pathway, Reactions, Metabolites, Study and Summary information.

Metabolomics Workbench are used. Internally, the MW database records disease and phenotype under the metadata field, *disease*. Hence, the phenotype is searched as a disease term within MW. The JSON files for each filter/category are curated and updated regularly and used to generate a pull-down menu. For the disease/phenotype filter, a two-step selection menu with slim (or disease class) terms in the first level and fine-grained terms in the second level is used for ease of presenting the options to the user. The user inputs from this page (main landing page) are submitted as a form, and a second page for MetGENE (as shown in Figure 4) is populated with the context-specific filtering terms. The second page comprises tabs for the search term associated entities, "Genes", "Pathways", "Reactions", "Metabolites", "Studies" and "Summary". The Summary tab displays the total number of pathways, reactions, metabolites and studies corresponding to each gene in the query. As MetGENE supports only those genes that encode proteins directly associated with metabolites, a warning is issued if the queried gene or a set of queried genes does not encode for such proteins.

Gene and Pathway Information Pages

The gene information page shown in Figure 5a presents gene IDs in different formats hyperlinked to the corresponding web pages pointing to repositories such as KEGG [4], GeneCards [5], NCBI [8], Ensembl [9], UniProt [10] and Marrvel [11]. The URLs to these repositories for the specific genes are constructed based on their base URLs and the respective supported gene ID types. This information is obtained from the REST API of the GICT and converted from JSON to a HTML table format for display purposes.

The pathway information page (Figure 5b) displays gene symbols hyperlinked with species and gene ID or symbol information as appropriate to various well-maintained pathway databases such as Pathway Commons [12], Reactome [13], KEGG [4] and Wikipathways [14]. MetGENE provides context-specific ease of access to these online resources.

Reaction and Metabolite Information Pages

The KEGG database provides the KEGG REST API to access information from the KEGG database. Given the organism code and the gene ID, the R KEGGREST API provides a way to access all the information, such as pathway IDs, reaction IDs, reaction names, reaction equations and compound (metabolites) IDs which are displayed in a tabular format. Figure 6a depicts the reaction information tab corresponding to the metabolic gene(s) of interest in a table view (one per gene) comprising reaction IDs hyperlinked to the corresponding KEGG reaction information page, reaction names and the

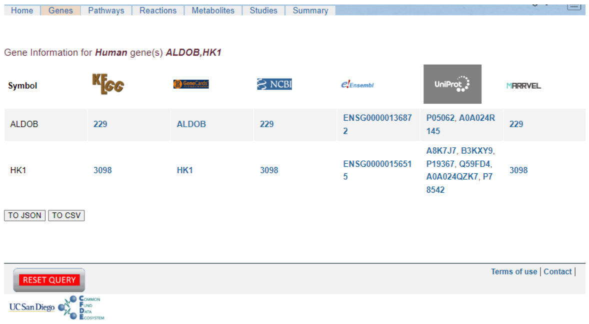

(a) Genes tab view.

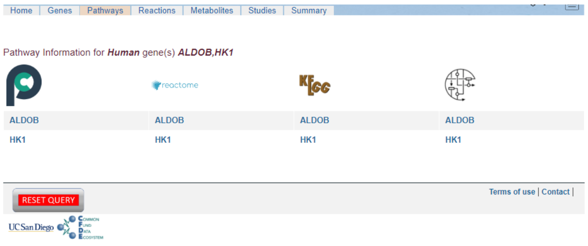

(b) Pathways tab view.

Figure 5. (a) MetGENE gene information page comprising Gene IDs in various formats corresponding to the searched gene(s) hyperlinked to various online resources. (b) MetGENE pathway information page comprising Gene IDs hyperlinked to various pathway resources.

reaction equation.

In the metabolites information tab, as shown in Figure 6b, a unique list of metabolites across all reactions corresponding to a given gene, along with their respective RefMet names (MW provides REST APIs to access the RefMet name corresponding to a KEGG compound ID), KEGG reaction IDs of all the reactions the metabolite participates in, are displayed. Further, the MW MetStat link provides access to the statistics about the metabolite measured across various studies in the MW database, filtered by anatomy (sample source) and disease/phenotype. This tool generates a report for any given metabolite in MW, comprising all the unique studies containing that metabolite and their median value of the relative standard deviation (RSD) across all those studies. The KEGG compound IDs that do not have RefMet names in the MW database display only the KEGG compound name and reaction IDs. The KEGG compound IDs are hyperlinked to the corresponding KEGG compound information page. MetGENE allows users to download the tables directly from the displayed page in JSON or CSV formats for further analysis. MW also provides REST APIs to access all the study IDs, titles, and RefMet names for a given KEGG compound ID in JSON, text and HTML formats.

MetGENE Metabolomics Study Information Page

In the Studies page (as shown in Figure 7a), a tabular view of the unique list of metabolites for the queried gene(s), their RefMet names hyperlinked to their corresponding description page in MW, and a comma-separated list of study IDs in which the metabolite participates (with each study ID linked to its corresponding study description page in MW) is presented. Further, a helpful text hover feature displaying the study title corresponding to a particular study ID is also provided to the user. MetGENE also allows users to select metabolites of interest and combine their studies for download and further analysis, as shown in Figure 7b.

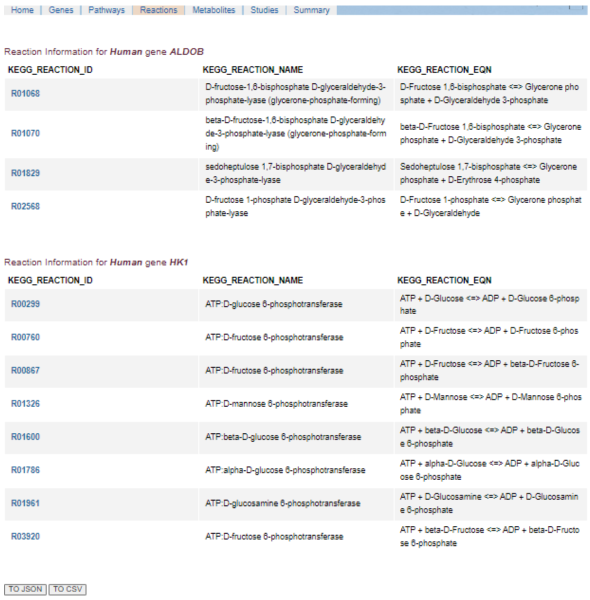

(a) Reactions tab view.

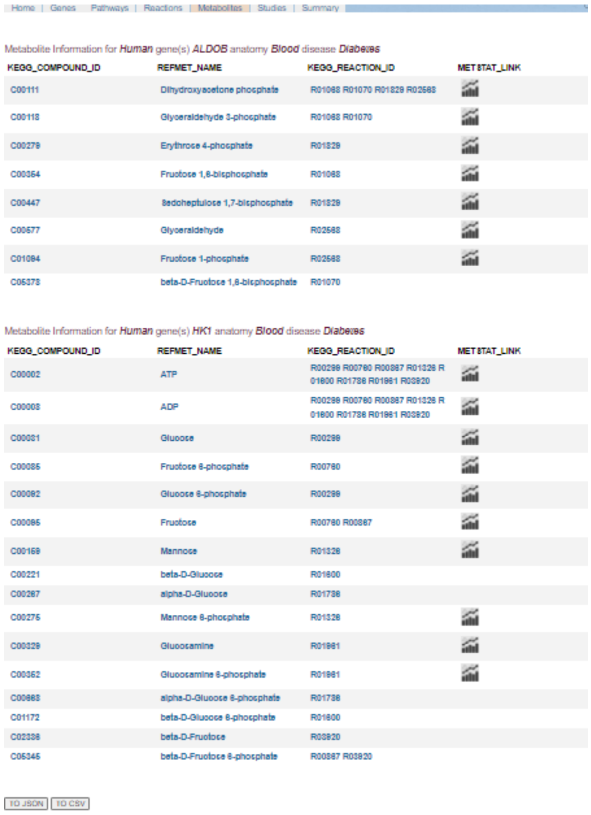

(b) Metabolites tab view.

Figure 6. (a) MetGENE reaction information page comprising KEGG Reaction IDs hyperlinked to KEGG reaction information page and reaction descriptions in a tabular format. (b) MetGENE metabolite information page comprises KEGG compound IDs, MW RefMet names, reaction IDs in which a metabolite participates in, hyperlinked to the KEGG reaction information page and MetStat link for the metabolite in a tabular format.

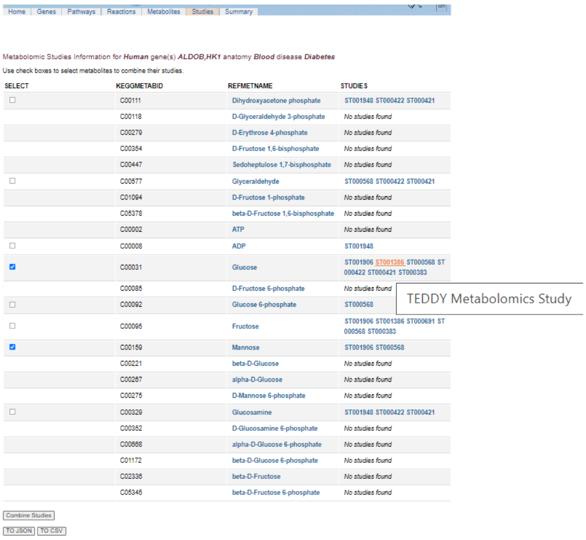

(a) Studies tab view.

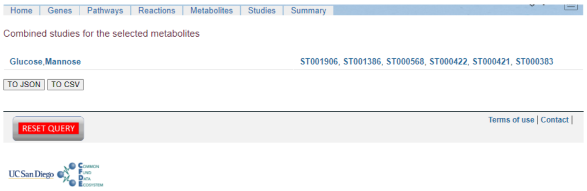

(b) Combined studies tab view.

Figure 7. (a) MetGENE metabolomics studies information page comprises KEGG compound IDs, RefMet names, and MW study IDs corresponding to a metabolite in a tabular format. (b) MetGENE allows users to combine studies for a selected set of metabolites.

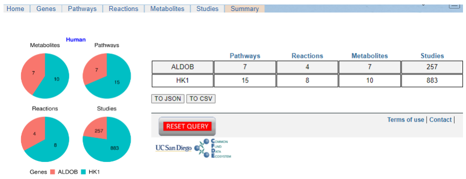

Figure 8. The Summary tab in MetGENE enumerates the total number of pathways, reactions, metabolites and metabolomic studies corresponding to each queried gene.

MetGENE Summary Page

In the Summary page (as shown in Figure 8), MetGENE displays the total number of pathways, reactions, metabolites and metabolomic studies for each queried gene and displays the information in a tabular format and a pie chart. This information is available for download both in JSON and CSV formats.

Case Study: Exploring gene PNPLA3 using MetGENE

Here, we demonstrate a use case that shows the utility of MetGENE as a one-stop tool to obtain all metabolomic information associated with a gene(s) in a specific disease condition. The protein Adiponutrin, encoded by the gene PNPLA3, is a multi-functional enzyme that belongs to the IPLA2/lipase family, which has both triacylglycerol lipase and acylglycerol O-acyltransferase activities. PNPLA3 is predominantly expressed in adipocytes and liver cells. It regulates the development of adipocytes and the metabolism of fats (lipogenesis and lipolysis). Diseases associated with PNPLA3 mutations include Fatty Liver Disease and Non-Alcoholic Steatohepatitis (NASH) [15],[16], [17] and [18].

To obtain information about a gene(s) and associated entities like enzymes, reactions, pathways and existing metabolomic studies in MW for a given context (fatty liver disease in humans), a user needs to identify precise key terms, perform a search on the internet, sift through the results to identify literature about various metabolomic studies and download the studies to perform downstream analyses. These steps are sometimes time-consuming and misleading, depending on the search terms' specificity. However, with MetGENE, the user can specify a gene ID(s) in any popular format and apply the filters on organism, anatomy, disease/phenotype to obtain genes, pathways, reactions, metabolites and studies information consolidated from various data resources, at one go. Supplementary Figure A1 shows the Gene tab for the PNLPA3 Gene. Links to various online resources (GeneCards, KEGG, NCBI, Uniprot and Marrvel) with genomic, transcriptomic, proteomic, clinical and functional information (via GeneCards link), module and pathway information (via KEGG), transcript and region information (via NCBI link), sequence, variant, gene expression information (via Ensembl link), protein sequence and functional information (via Uniprot link) and gene variants associated with PNLPA3 (via Marrvel link) are provided in the gene information table. Supplementary Figure A2 represents the Pathway tab for the PNLPA3 gene with links to interactions with and pathways that involve PNPLA3 (via Pathway Commons), protein and reaction location information (via Reactome), pathway definitions (via KEGG), and pathway-related collaborative information (via Wikipathways). Supplementary Figure A3 represents the Reactions and Metabolites tabs for PNPLA3. It delineates two hydrolysis reactions where diacylglycerol (DAG) is hydrolyzed to 2-monoacylglycerol (2-MAG), and triacylglycerol (TAG) is hydrolyzed to DAG by PNPLA3. Three generic compounds (TAG, DAG, and Fatty acid) participate in the reactions. The Metabolites tab for PNPLA3 lists all the metabolites (with substitutions for TAG and DAG) along with corresponding RefMet names or KEGG metabolite names (in the absence of RefMet names) along with a MetStat link that points to metabolite statistics information such as a histogram of the RSD (Relative Standard Deviation) metabolite data, ANOVA results for the metabolite with a cut-off p-value in MW via MetStat, with the anatomy and disease filters applied. Supplementary Figure A4 depicts the Studies tab in MetGENE for the gene PNPLA3. Each metabolite would display the corresponding Study IDs if measured/listed in any studies deposited in MW, hyperlinked to the study description. A hover text displays the study title. The tool provides the user with the ability to combine studies for metabolites of interest to a consolidated view, as shown in Supplementary Figure A4. Supplementary Figure A5 serves as an informative visual aid, providing an overview of the information flow network within MetGENE for the PNPLA3 use case. It simplifies the complexity of the underlying technical details of MetGENE, facilitating comprehension of the query-to-results process. All of MetGENE tables can be downloaded in formats such as JSON and CSV directly from the respective pages in the browser or via the REST API. The REST API supports JSON and text formats. They are deposited in the Smart API repository along with the accompanying documentation.

Discussion

Given a gene(s), the MetGENE tool identifies associations between the gene(s) and the metabolites that are biosynthesized, catabolized, or transported by proteins coded by those gene(s). It is a knowledge-based data aggregator, accessing and integrating data from resources such as the KEGG and Metabolomics Workbench. The gene(s) link to metabolites, the chemical transformations involving the metabolites through gene-specified proteins/enzymes, the functional association of these gene-associated metabolites and the pathways involving these metabolites with context-based filtering based on anatomy (sample source), disease/phenotype. The user can specify the gene using a multiplicity of IDs, and the gene

ID conversion tool translates these into harmonized IDs that are the basis for metabolite associations. Further, all studies involving the metabolites associated with the gene-coded proteins, as present in the Metabolomics Workbench (MW), will be accessible to the user as a stand-alone tool or via the portal interface for the NIH Common Fund National Metabolomics Data Repository (NMDR). The user can begin their journey either from the main web page for MetGENE (see Availability) or from the NIH Common Fund Data Ecosystem (CFDE) portal (https://app.nih-cfde.org/; the steps are: Data Browser → Vocabulary → Gene).

Potential implications

Features from MetGENE will contribute to the integration of other omics data to draw metabolomics perspectives and with metabolomics data, with genes serving as the bridging nodes. For example, tools such as MetGENE will assist a researcher in interpreting the results of multi-omics data integration holistically, where they can consider both gene-related and metabolomics data in a metabolic pathway. We are also in the process of integrating MetGENE with tools from other DCCs as a part of a broader NIH-CFDE initiative. Through that, we can provide support for persistent and shareable MetGENE-based workflows.

Availability of Supporting Code and Requirements

MetGENE is an open-source collaborative initiative available at GitHub. The main website of MetGENE and the Smart APIs are available, as shown below. MetGENE is registered with the SciCrunch registry (RRID:SCR_023402 (https://scicrunch.org)). We also plan to register MetGENE-based workflows as BioCompute Objects (BCOs) in the Galaxy Community Hub (https://galaxyproject.org/) in the near future as a part of NIH-CFDE efforts.

- Project Name: MetGENE
- Project Home Page: https://bdcw.org/MetGENE/index.php
- Operating System: Platform independent
- Programming languages: R and PHP
- Smart APIs: https://smart-api.info/registry/?q=MetGENE
- Source code: https://github.com/metabolomicsworkbench/MetGENE

Additional Files

- Supplementary Figure A1: The gene information tab for PNPLA3 in MetGENE has links to various online resources that provide different types of information pertaining to the gene, including sequence, gene expression, protein structure, functional and disease information.
- Supplementary Figure A2: The pathway information tab for PNPLA3 in MetGENE has links to various online resources that provide different types of information pertaining to the pathways in which PNPLA3 participates.
- Supplementary Figure A3: The reaction and metabolite information tabs for PNPLA3 in MetGENE have links to reactions controlled by PNLPA3, with Reaction IDs linked to the KeggKEGG reaction definition page. RefMet provides the metabolite details, and the MetStat link provides the context-specific metabolite measurement statistics.
- Supplementary Figure A4: The studies information tab for PNPLA3 in MetGENE has links to studies in MW pertaining to the metabolites listed. Studies can be combined for selected metabolites for further analysis.
- Supplementary Figure A5: A consolidated network of information flow for gene PNPLA3 demonstrating how pathways, reactions, metabolites, studies and summary are connected in the result.

Declarations

List of abbreviations

- CFDE - Common Fund Data Ecosystem
- DCC - Data Coordination Centers
- GTEx - Gene Tissue Expression
- LINCS - Library of Integrated Network-Based Cellular Signatures
- GICT - Gene ID Conversion tool
- MW - Metabolomics Workbench
- NMDR - National Metabolomics Data Repository

Ethical Approval

Not Applicable.

Consent for publication

Not Applicable.

Competing Interests

Not Applicable.

Funding

This work has been supported by the National Institutes of Health Grants (Metabolomics Workbench, U2C-DK119886 and Common Fund Data Ecosystem (CFDE) OT2-OD030544).

Author’s Contributions

This work was conceptualized by Sh.S., Su.S., M.R.M. and S.R. The funding acquisition was by Sh.S. The methodology, software development and visualization for MetGENE were developed by Su.S. and M.R.M. Validation and testing was performed by Su.S., M.R.M., S.R. and Sh.S. The disease ontology terms and structuring was provided by S.R. The Metabolomics Workbench REST APIs that are used in this work were developed by E.F. The original draft for the paper was prepared by Su.S and reviewing and editing was done by Sh.S, M.R.M. and S.R. The project administration was done by Sh.S and M.R.M.

Acknowledgements

We thank the Common Fund Data Ecosystem Gene Working Group for useful discussions.

References

1. Sud M, Fahy E, Cotter D, Azam K, Vadivelu I, Burant C, et al. Metabolomics Workbench: An international repository for metabolomics data, metadata and metabolite standards, protocols, tutorials and training, and analysis tools. Nucleic acids research 2016;44:D463–70. http://www.metabolomicsworkbench.org.
2. GTExConsortium. Human genomics. The Genotype-Tissue Expression (GTEx) pilot analysis: multitissue gene regula-

tion in humans. Science 2015;348(6235):648–660. https://gtexportal.org/home/.
3. V S, J T, A K, D V, D C, M FN, et al. LINCS Data Portal 2.0: next generation access point for perturbation-response signatures. Nucleic Acids Research 2020;48:D431–D439.
4. Kanehisa M, Goto S. KEGG: kyoto encyclopedia of genes and genomes. Nucleic Acids Research 2000;28(1):27–30. https://www.genome.jp/kegg/kegg1.html.
5. Safran M, Rosen N, Twik M, BarShir R, Stein TI, Dahary D, et al. In: The GeneCards Suite Singapore: Springer Singapore; 2021. p. 27–56. https://doi.org/10.1007/978-981-16-5812-9_2.
6. R C, R B, IM K, A K, M K, PE M, et al. The MetaCyc database of metabolic pathways and enzymes - a 2019 update. Nucleic Acids Research 2020;48:D445–D453.
7. Schloerke B, Allen J. plumber: An API Generator for R; 2022, https://www.rplumber.io, https://github.com/rstudio/plumber.
8. S F. The NCBI Taxonomy database. Nucleic acids research. Nucleic Acids Research 2012;40:D136–D143. https://doi.org/10.1093/nar/gkr1178.
9. Cunningham F, Allen JE, Allen J, Alvarez-Jarreta J, Amode MR, Armean IM, et al. Ensembl 2022. Nucleic Acids Research 2022;50(1):D988–D995. https://doi.org/10.1093/nar/gkab1049.
10. Consortium TU. UniProt: the universal protein knowledgebase in 2021. Nucleic Acids Research 2021;49(D1):D480–D489. https://doi.org/10.1093/nar/gkaa1100.
11. Wang J, Al-Ouran, R, Hu, Y, Kim, et al. MARRVEL: Integration of Human and Model Organism Genetic Resources to Facilitate Functional Annotation of the Human Genome. American journal of human genetics 2021;100(6):843–853. https://doi.org/10.1016/j.ajhg.2017.04.010.
12. Cerami EG, Gross BE, Demir E, Rodchenkov I, Babur O, Anwar N, et al. Pathway Commons, a web resource for biological pathway data. Nucleic Acids Research 2010;39:D685–D690. https://www.pathwaycommons.org/.
13. Gillespie M, Jassal B, Stephan R, Milacic M, Rothfels K, Senff-Ribeiro A, et al. The reactome pathway knowledgebase 2022. Nucleic Acids Research 2021;50(D1):D687–D692. https://reactome.org/.
14. M M, A A, A R, A W, DN S, K H, et al. WikiPathways: connecting communities. Nucleic Acids Research 2021;49:D613–D621. https://doi.org/10.1093/nar/gkaa1024.
15. Cohen J, Horton J, HH H. Human fatty liver disease: old questions and new insights. Science 2011;332(6037):1519–23.
16. Gorden D, Myers D, Ivanova P, Fahy E, Maurya M, Gupta S, et al. Human fatty liver disease: old questions and new insights. Science 2011;332(6037):1519–23.
17. P P, S R. The role of PNPLA3 in health and disease. Biochem Biophys Acta Mol Cell Biol Lipids 2019;1864(6):900–906.
18. XC D. PNPLA3—A Potential Therapeutic Target for Personalized Treatment of Chronic Liver Disease. Front Med 2019;6(304).

Gene Information View PNPLA3

Click here to access/download
Supplementary Material
FigureA1_Supplementary.png

Pathway Information View PNPLA3

Click here to access/download

Supplementary Material

FigureA2_Supplementary.png

Reaction and Metabolite Information PNPLA3

Click here to access/download
Supplementary Material
FigureA3_Supplementary.png

Click here to access/download
Supplementary Material
FigureA4_Supplementary.png

Information flow for query PNPLA3

Click here to access/download
Supplementary Material
FigureA5_Supplementary.png

Letter to Editor Click here to access/download;Personal Cover;FinalCoverLetter.docx

UNIVERSITY OF CALIFORNIA, SAN DIEGO UCSD

BERKELEY • DAVIS • IRVINE • LOS ANGELES • MERCED • RIVERSIDE • SAN DIEGO • SAN FRANCISCO SANTA BARBARA • SANTA CRUZ

Shankar Subramaniam
Joan and Irwin Jacobs Professor of Bioengineering & Systems Biology
Department of Bioengineering
University of California at San Diego
9500 Gilman Drive
La Jolla, CA 92093-0427
SHANKAR@UCSD.EDU
Tel: (858) 822-0986

Dr. Nicole Nogoy
Executive Editor, GigaScience

August 18, 2023

Sub: Submission of the revised manuscript to GigaScience: GIGA-D-23-00021R1

Dear Dr Nogoy,

We thank you for your positive feedback regarding our manuscript and considering it as potentially acceptable. We would also like to thank the reviewers for their comments and feedback on the revised manuscript. We have revised the manuscript again in the manner you suggested and incorporated sentences detailing the importance of MetGENE with a use case scenario in the Introduction section (major edits are highlighted in the main manuscript). We believe the manuscript reads better now and has improved considerably. We hope you will find the revised manuscript publishable. Below, we summarize the main changes and a detailed response to the comments by both reviewers is included in the submission as a separate document.

Summary of main changes:

1. We have clarified that MetGENE deals with only genes that encode for metabolic enzymes. We are not including transporters since we could not find a convenient resource and API to get information on metabolite transport related enzymes. We will try to incorporate this in the future.
2. We have motivated the need for MetGENE and its novelty by highlighting its useful features compared to existing databases like GeneCards and MetaCyc as suggested by you and Reviewer #2 (we provide a comprehensive argument in our response to reviewer's comments).

We passionately believe that MetGENE represents a significant advancement in user convenience and research capabilities by bridging a crucial gap that allows biomedical researchers to seamlessly access metabolomic studies relevant to their research that no other tool provides today. It is scalable as it allows gene sets comprising of multiple genes as opposed to single gene queries that other tools provide and shields the users from the complexities of data format conversions. Its APIs are currently being used to build comprehensive workflows via the NIH-CFDE partnership that will allow for the creation of persistent workflows, integrating information from all other CFDE data resources. Therefore, we would like to submit our article under the category of Research, and not as a Technical Note. Thank you again for the opportunity to submit our work to GigaScience. We look forward to receiving your feedback.

Sincerely,

Shankar Subramaniam

Shankar Subramaniam, Ph.D.

Response to Reviewers
Click here to access/download;Personal Cover;Response to Reviewer Comments_Final.docx

Response to Reviewer Comments

Reviewer 1

The authors have addressed my comments and suggested revisions satisfactorily.

Response: We thank the reviewer for the time and effort taken to review our revised manuscript.

One small comment - on the current entry page (https://bdcw.org/MetGENE/index.php), the "Select disease/phenotype" dropdown menu is duplicated when I access the URL (using a Chrome browser). The authors should make sure this is fixed prior to publication.

Response: This problem has been fixed. There is a dropdown menu for selecting the Disease/Phenotype category, followed by another dropdown menu (cascading filters) to select the specific Disease/Phenotype itself. This design is implemented to prevent users with an overwhelming list of diseases/phenotypes while offering them organized categories of disorders to choose from. This is mentioned in the manuscript.

Reviewer 2

I still insist on my comments about comparing with other similar approaches. Starting from a gene (or metabolite or proteins), a biologist can easily get a similar amount of information from MetaCyc or GeneCards. Many of those databases are already listed in the paper. In MetaCyc (and other species-specific databases), searching from genes, metabolites, and pathways is quite easy and results are curated and summarized (not as links). I am still unclear what is the additional value provided by this paper. Many of those databases/webs are already built as a summary or one-stop place for different information sources (e.g., GeneCards and MetaCyc).

Response: We thank the reviewer for the comments. While GeneCards offers comprehensive gene-related information (albeit not conveniently accessible for metabolites, even with hyperlinked ChEBI IDs), and MetaCyc furnishes insights into genes, their associated pathways, and reactions, to the best of our knowledge, no other tool effectively establishes a direct connection between a gene(s) and its pertinent metabolomic studies and datasets in the manner accomplished by MetGENE. This is important for several reasons.

We consider a scenario where a biomedical researcher aims to uncover therapeutic targets for a gene like IDH1, which plays a crucial role in the initial stages of tumorigenesis across various cancers. Given its metabolic significance, the researcher may want to find specific metabolomic studies and their findings (e.g., statistical, and biological insights) relevant to cancer. Without MetGENE, users would be compelled to manually compile a comprehensive list of metabolite names associated with a specific gene, such as IDH1, by extracting information from sources like MetaCyc. This endeavor would be

arduous due to the lack of a streamlined download option for acquiring all metabolites linked to a particular gene within MetaCyc.

Taking the case of IDH1 as an example, MetaCyc designates isocitrate—a metabolite participating in the reaction catalyzed by IDH1—as D-threo-isocitrate. Converting this designation to either a KEGG ID or an INCHI key from a different database would be necessary. Subsequently, this must be input into the Metabolomics Workbench to uncover pertinent studies with relevant filters.

The resulting metabolite list could become substantial for genes such as HK1 or PNPLA3 that catalyze numerous reactions, making the entire process time-consuming, inefficient, and prone to errors. This approach would also involve managing discrepancies in metabolite nomenclature, necessitating the conversion of names from one format to another. MetGENE addresses these challenges comprehensively, eliminating the need for manual intervention and reducing the risk of errors. By automating the conversion of metabolite names to RefMet names, a format comprehensible to the Metabolomics Workbench using the MW API behind the scenes, it facilitates direct connections between gene(s) and pertinent metabolomic studies that are further contextualized by condition and anatomy. This not only drastically reduces the time and effort required by users but also streamlines the exploration of relevant data on a single, accessible platform.

Furthermore, MetGENE goes beyond offering information about individual genes; it extends its capabilities to cover entire gene sets in one seamless process. When users identify a specific subset of genes within a particular context, MetGENE stands out by efficiently processing the full list. This differs from fetching information individually, as commonly seen in platforms like GeneCards, MetaCyc, or KEGG.

Our ongoing efforts involve the integration of MetGENE APIs with NIH-CFDE's Playbook Partnership initiative, which is developing tools to build workflows by facilitating the integration of API-based decentralized tools. This integrated approach enhances efficiency and represents a significant advancement in user convenience and research capabilities. We have added this information in the introduction as a motivator for MetGENE.

It also seems to me that the manuscript might be providing support to MW data and a bioinformatics data workflow. If this is the case, then providing more details on the workflow and integrated usage cases might be helpful.

Response: We have extensively illustrated the utility of MetGENE through a use case involving the gene PNPLA3 in the manuscript. Furthermore, we have incorporated a scenario exemplifying the motivation behind a tool like MetGENE, focusing on the gene IDH1 and accentuating its innovative features. To augment MetGENE's functionalities, we are in the process of including additional links beyond statistics. These links will facilitate users performing metabolite enrichment on selected metabolites via MetGENE, seamlessly integrating with the Metabolomics Workbench.

Furthermore, we underscore the ongoing integration of MetGENE APIs into the NIH CFDE Playbook Partnership tool. This integration empowers users to conduct cross-queries across diverse CFDE resources, constructing meaningful and persistent workflows that expand their research capabilities.

At least, additional visualization on the web for the users and a summary table instead of links should be provided. In addition, the correctness of the link should be checked.

Response: We believe that providing additional information from other resources (in the Genes and Pathway tabs) would duplicate information and clutter the views. Instead, taking the user to the actual resource would help in utilizing the resource more effectively. We have used a tool named DeadLinkChecker (DeadLinkChecker.com) and found no dead links. To the best of our knowledge, there are no incorrect links in MetGENE.

The writing seems fine though it can be friendlier to laypersons by reducing the amount of abbreviation and technical details in results.

Response: We thank the reviewer for the suggestion. We have made sure we have elaborated all the abbreviations before they are used. We have tried to minimize unnecessary technical jargon in the revised manuscript.
