# Supplementary material for: MetGENE: gene-centric metabolomics information retrieval tool
Source: Gigascience. 2023 Nov 20;12:giad089. doi: 10.1093/gigascience/giad089 (PMC10659118; doi:10.1093/gigascience/giad089)
Supplement: giad089_Response_to_Reviewer_Comments_Original_Submission [file giad089_response_to_reviewer_comments_original_submission.pdf]

Author's Response To Reviewer Comments

Main comments

Reviewer 1:

1. Overall the knowledge-base is well designed, with a description of the data retrieval mechanism on the query page. However, the term "associated" metabolites to a gene is rather open-ended. It seems that the association is via the encoded protein to a gene, and its known enzymatic activity. This then links the gene to a metabolite, and known pathways where the enzyme is active. There are many other ways that a gene may have an "association" with a small molecule metabolite however - such as via regulation of the gene or its protein product with small molecules metabolites, literature-based connections, gene expression-metabolite associations etc. It may help the user to know what associations are not a part of the analysis, so the user knows what to expect in terms of how information is retrieved from any given query.

Response: We thank the reviewer for this important comment. We have added the following line in the Abstract section, "MetGENE focuses only on those genes that encode for proteins directly associated with metabolites. All other gene-metabolite associations are beyond the current scope of MetGENE." We have also elaborated on this point in the Introduction section, "There are many other ways a gene may have an "association" with a small molecule metabolite, such as via gene regulation (e.g., TF-target relationship) or its protein product, gene expression-metabolite association, protein-protein-metabolite association, etc. However, in MetGENE, we only focus on those genes that encode for proteins directly associated with metabolites; these include metabolic enzymes, transporters and receptors.". We have included the number of associations supported by MetGENE in Figure1C.

2. Related to the point above, a key linkage of a gene to a metabolite is the encoded protein that has catalytic activity. Although it is possible to get to the information on the protein and its enzymatic activity via the Uniprot link via a couple of clicks by the user, it seems that the Uniprot link could be displayed more prominently on the initial results page after the query is entered. This may be a more efficient way for users to get information on the protein, as a key linkage point to the metabolite/pathway information.

Response: We thank the reviewer for this suggestion, and we agree that providing Uniprot link in the main screen may be of help to the user. However, for a given gene, there are several Uniprot IDs and displaying all this information in the main screen would lead to visual cluttering ad hence we decided to display this in the Genes tab along with other useful set of links such as KEGG, Ensembl, etc. which may provide more information to the user.

3. It is understood that the "phenotype" query is not available yet. However, it's unclear what type of queries will be available here. Could the authors provide some information on the options for phenotypes that they plan to make available?

Response: In the current version on MetGENE, we decided to completely do away with the Phenotype menu in the query screen. We have combined both the Disease as well as the Phenotype into a consolidated two step cascading menu list. We have updated Figure 3 in the manuscript to reflect this change. The Metabolomics Workbench does not recognize "Phenotype" as a separate search term and hence we decided to consolidate both disease and phenotype into a single list. However, in the future, if MW decides to support querying based on the Phenotype terms from databases such as Human Phenotype Ontology, we will support it.

4. They authors should be more clear on exactly how many organisms can be queried - at this point it looks like it is a relatively limited number (human, mouse, rat). Are there plans to include any more organisms - such as pathogenic bacteria or other organisms related to human health that might be of interest?

Response: We agree with the reviewer and our apologies for not making it clear in our manuscript. We will

include microorganisms relevant to human health and disease in a phased manner. We plan to include E. coli (K12), C. elegans, D. melanogaster, and A. gambiae soon and have mentioned this in the Result sections of the manuscript.

5. In test runs it might be nice to show the number of returned results under "Metabolites", "Reactions", "Pathways" in the first window. Some may be empty and it seems inefficient for the user to have to click through each category only to find out there are not any results?

Response: We thank the reviewer for an excellent suggestion. Since the time taken to compute the number of results can be high in the case of multiple genes or genes who have large number of metabolites associated with them, adding this feature to the main landing page would slow down the loading of the page which is undesirable. We have created a “Summary” tab that displays the number of pathways, reactions, metabolites, and the studies both as a downloadable table as well as a pie chart graphic for better visualization (Figure 4). In addition, we have also added a feature where the tool warns the user about empty results if the gene does not encode a protein directly related to metabolites.

6. The "clear query" button to start a new query is a bit small - this could be more obvious to the user to clear the form and start again.

Response: We have increased the size and the graphic on the button to clear the query to address this issue.

Reviewer 2:

1. There are many methods/databases/tools linking genes to pathways, reactions, and metabolites. For example, MetaCyc or HumanCyc provides web and local interfaces, where the search can start from genes, proteins, pathways, or metabolites. Related information can also be easily fetched (e.g., from genes to related reactions or metabolites). Those databases tend to be curated, which increases accuracy but can probably miss possible connections. This manuscript provides database searching that is not curated which can possibly provide more connections. However, such comparisons are not stated in the manuscript. In addition, many of the databases (KEGG, GeneCards) the authors used and mentioned have blocks of genes, pathways, and metabolites. The authors need to explain the significance and improvement of MetGENE upon other databases or curated tools. Is MetGENE going to provide more complete results than any of them? How do MetGENE results tradeoff between coverage and accuracy compared with others?

Response: MetGENE uses a knowledge graph to relate metabolic genes to the metabolites and then downstream to metabolomic studies. The main value proposition of MetGENE is to get studies from MW by providing a gene centric front end. MetGENE is not a database and does not compete with other databases that provides Gene ◊ Pathways ◊ Reactions ◊ Metabolite connections but merely provides a context by becoming a one stop place for accessing other databases in context. Hence, we are not in a position to provide trade-off result comparing to other databases.

2. It seems that the interface with the metabolic workbench is one of the major innovations of this manuscript. The tool and visualization provided for this functionality are however limited. It's mainly through the block of metabolites (METSTAT) and studies, which indeed seem to provide interesting information. Can the author show how MetGENE helps improve users' understanding or efficiency through such integration with a specific biological example? How does integrating results of multiple workbench studies show something not directly visible in one? Some additional functionality in this part can be helpful to the users (e.g., a table of quantification and corresponding metabolites approaches of the study through API OR a summary information table for the study included in the Study page).

Response: At the moment, we are not providing any more extra insights by combining studies from MW. However, in the context of quantification of metabolites, METSTAT already provides summary (such as distribution of values of metabolites) based on information from multiple studies. Study summarization with respect to different types of metadata can be done (e.g., type of chromatography, MS/NMR etc). However, integration of information based on metadata (e.g., 6 studies were GC-MS, 4 Studies were LC-MS, etc.) may not be that useful. If we come across a use case that requires integration of multiple

studies, we will incorporate it into MetGENE in the future.

3. The presentation of this paper has been mainly qualitative. More quantitative evaluation and presentations can help. For example, what's the size of the search space of genes for MetGENE to have any results? How many reactions, metabolites, pathways, and studies are connected?
Response: We thank the reviewer for the suggestion. Now, we support 1069 metabolic genes. We have represented statistics of the associations in Figure 1C.

4. Only allowing starting from genes seems limited. The search should also be able to start with metabolites or reactions.
Response: We agree with the suggestion but expanding it to start from Reaction or Pathway is out of the current scope of MetGENE. Also, it would be hard for users to specify the Reaction/Pathway name or description as a starting point to query. We also note that the Metabolomics Workbench (https://www.metabolomicsworkbench.org/databases/refmet/refmet.php) already enables connecting metabolites to genes, reactions, and pathways.

5. Even though explained in the method, it's not clear to me what kind of connections between genes, pathways, reactions, and metabolites the authors used for the results. A supplementary figure showing the logical process of searching starting from one gene can be helpful. Particularly, the user can present a network starting from one specific gene (e.g., PNPLA3) and present how different parts (e.g., metabolites, pathways) are connected in the result.
Response: As per the suggestion of the reviewer, we have added an additional figure in the Supplementary Material (Figure A5) that depicts the information flow from query to the results for the use case of the gene PNPLA3.

Minor comments:

6. The pathway result is puzzling as it seems to be links connected to different databases searching for information related to the genes. When I click those links, the information is not solely on pathways. Supplying all the information in a table rather than links can be much easier for the user. The WikiPathways link seems also not working. Similarly, the author can add more information for the tables of reactions (showing the reaction equation itself).
Response: The reason MetGENE is designed to display a list of Pathway database links as opposed to the pathways themselves is to enable the user to have access to different types of information each of these databases provide. One could potentially scrape this information and display consolidated information but to decide what information is useful to display or not would be difficult to assess. As per the reviewer's suggestion, we have included the reaction equation as part of the Reactions table (Figure 6a). We have also updated our REST and Smart APIs to reflect this.

7. It seems white space after the comma when searching for two genes will cause problems.
Response: We have fixed this issue in the current version of the tool. We thank the reviewer for pointing it out.

8. There are many technical details in the result which can be put into methods or supplements. For example, the sentence "Given the three-letter KEGG organism code and the ENTREZ gene ID of the gene, the R KEGGREST API provides a way to access all the information such as pathway IDs, reaction IDs and compound (metabolites) IDs as a data frame objects" can be simplified without mention code length, specific ID, specific API methods, etc. The "MetGENE Query Interface" section of the result also seems too detailed and technical. The result part can be rewritten and reduced to improve accessibility to general readers.

Response: We appreciate the reviewer's feedback regarding simplifying the writing. We have simplified the writing in this revised version of the manuscript (Methods section).
