# Supplementary material for: MetGENE: gene-centric metabolomics information retrieval tool
Source: Gigascience. 2023 Nov 20;12:giad089. doi: 10.1093/gigascience/giad089 (PMC10659118; doi:10.1093/gigascience/giad089)
Supplement: giad089_Response_to_Reviewer_Comments_Revision_1 [file giad089_response_to_reviewer_comments_revision_1.pdf]

Author's Response To Reviewer Comments

Reviewer 1

Reviewer: The authors have addressed my comments and suggested revisions satisfactorily.

Response: We thank the reviewer for the time and effort taken to review our revised manuscript.

Reviewer: One small comment - on the current entry page (https://bdcw.org/MetGENE/index.php), the "Select disease/phenotype" dropdown menu is duplicated when I access the URL (using a Chrome browser). The authors should make sure this is fixed prior to publication.

Response: This problem has been fixed. There is a dropdown menu for selecting the Disease/Phenotype category, followed by another dropdown menu (cascading filters) to select the specific Disease/Phenotype itself. This design is implemented to prevent users with an overwhelming list of diseases/phenotypes while offering them organized categories of disorders to choose from. This is mentioned in the manuscript.

Reviewer 2

Reviewer: I still insist on my comments about comparing with other similar approaches. Starting from a gene (or metabolite or proteins), a biologist can easily get a similar amount of information from MetaCyc or GeneCards. Many of those databases are already listed in the paper. In MetaCyc (and other species-specific databases), searching from genes, metabolites, and pathways is quite easy and results are curated and summarized (not as links). I am still unclear what is the additional value provided by this paper. Many of those databases/webs are already built as a summary or one-stop place for different information sources (e.g., GeneCards and MetaCyc).

Response: We thank the reviewer for the comments. While GeneCards offers comprehensive gene-related information (albeit not conveniently accessible for metabolites, even with hyperlinked ChEBI IDs), and MetaCyc furnishes insights into genes, their associated pathways, and reactions, to the best of our knowledge, no other tool effectively establishes a direct connection between a gene(s) and its pertinent metabolomic studies and datasets in the manner accomplished by MetGENE. This is important for several reasons.

We consider a scenario where a biomedical researcher aims to uncover therapeutic targets for a gene like IDH1, which plays a crucial role in the initial stages of tumorigenesis across various cancers. Given its metabolic significance, the researcher may want to find specific metabolomic studies and their findings (e.g., statistical, and biological insights) relevant to cancer. Without MetGENE, users would be compelled to manually compile a comprehensive list of metabolite names associated with a specific gene, such as IDH1, by extracting information from sources like MetaCyc. This endeavor would be arduous due to the lack of a streamlined download option for acquiring all metabolites linked to a particular gene within MetaCyc.

Taking the case of IDH1 as an example, MetaCyc designates isocitrate—a metabolite participating in the reaction catalyzed by IDH1—as D-threo-isocitrate. Converting this designation to either a KEGG ID or an INCHI key from a different database would be necessary. Subsequently, this must be input into the Metabolomics Workbench to uncover pertinent studies with relevant filters.
The resulting metabolite list could become substantial for genes such as HK1 or PNPLA3 that catalyze numerous reactions, making the entire process time-consuming, inefficient, and prone to errors. This approach would also involve managing discrepancies in metabolite nomenclature, necessitating the conversion of names from one format to another. MetGENE addresses these challenges comprehensively, eliminating the need for manual intervention and reducing the risk of errors. By automating the conversion of metabolite names to RefMet names, a format comprehensible to the Metabolomics Workbench using the MW API behind the scenes, it facilitates direct connections between gene(s) and pertinent metabolomic studies that are further contextualized by condition and anatomy. This not only drastically reduces the time and effort required by users but also streamlines the exploration of relevant data on a single, accessible

platform.

Furthermore, MetGENE goes beyond offering information about individual genes; it extends its capabilities to cover entire gene sets in one seamless process. When users identify a specific subset of genes within a particular context, MetGENE stands out by efficiently processing the full list. This differs from fetching information individually, as commonly seen in platforms like GeneCards, MetaCyc, or KEGG.
Our ongoing efforts involve the integration of MetGENE APIs with NIH-CFDE's Playbook Partnership initiative, which is developing tools to build workflows by facilitating the integration of API-based decentralized tools. This integrated approach enhances efficiency and represents a significant advancement in user convenience and research capabilities. We have added this information in the introduction as a motivator for MetGENE.

Reviewer: It also seems to me that the manuscript might be providing support to MW data and a bioinformatics data workflow. If this is the case, then providing more details on the workflow and integrated usage cases might be helpful.

Response: We have extensively illustrated the utility of MetGENE through a use case involving the gene PNPLA3 in the manuscript. Furthermore, we have incorporated a scenario exemplifying the motivation behind a tool like MetGENE, focusing on the gene IDH1 and accentuating its innovative features. To augment MetGENE's functionalities, we are in the process of including additional links beyond statistics. These links will facilitate users performing metabolite enrichment on selected metabolites via MetGENE, seamlessly integrating with the Metabolomics Workbench.
Furthermore, we underscore the ongoing integration of MetGENE APIs into the NIH CFDE Playbook Partnership tool. This integration empowers users to conduct cross-queries across diverse CFDE resources, constructing meaningful and persistent workflows that expand their research capabilities.

Reviewer: At least, additional visualization on the web for the users and a summary table instead of links should be provided. In addition, the correctness of the link should be checked.

Response: We believe that providing additional information from other resources (in the Genes and Pathway tabs) would duplicate information and clutter the views. Instead, taking the user to the actual resource would help in utilizing the resource more effectively. We have used a tool named DeadLinkChecker (DeadLinkChecker.com) and found no dead links. To the best of our knowledge, there are no incorrect links in MetGENE.

Reviewer: The writing seems fine though it can be friendlier to laypersons by reducing the amount of abbreviation and technical details in results.

Response: We thank the reviewer for the suggestion. We have made sure we have elaborated all the abbreviations before they are used. We have tried to minimize unnecessary technical jargon in the revised manuscript.
