# Supplementary material for: MetGENE: gene-centric metabolomics information retrieval tool
Source: Gigascience. 2023 Nov 20;12:giad089. doi: 10.1093/gigascience/giad089 (PMC10659118; doi:10.1093/gigascience/giad089)
Supplement: giad089_Response_to_Reviewer_Comments_Revision_2 [file giad089_response_to_reviewer_comments_revision_2.pdf]

Author's Response To Reviewer Comments

We have made the changes requested by the editor.
