# Supplementary material for: MetGENE: gene-centric metabolomics information retrieval tool
Source: Gigascience. 2023 Nov 20;12:giad089. doi: 10.1093/gigascience/giad089 (PMC10659118; doi:10.1093/gigascience/giad089)
Supplement: giad089_Reviewer_1_Report_Original_Submission — Timothy Griffin -- 2/26/2023 [file giad089_reviewer_1_report_original_submission.pdf]

Reviewer Report

Title: MetGENE: Gene-centric Metabolomics Information Retrieval Tool

Version: Original Submission **Date:** 2/26/2023

Reviewer name: Timothy Griffin

Reviewer Comments to Author:

The authors present a new resource called MetGene, a knowledge-base developed to provide gene-centric information on metabolites and biological pathways tied to annotated genes. The user interface enables searching for known, annotated genes from several organisms, along with disease or even phenotype specific context, and retrieves known information on connected enzyme driven processes, such as metabolites associated with the gene, pathways and specific reactions. Users may also download results from these queries in tabular formats or potentially integrate query outputs with other computing platforms. Overall, the resource could have high value for researchers seeking to understand gene-metabolite relationships and connectivity to biological pathways of interest. In reviewing the knowledgebase, some suggestions for modifications that may improve the utility for users are noted and describe below.

Main comments

1. Overall the knowledge-base is well designed, with a description of the data retrieval mechanism on the query page. However, the term "associated" metabolites to a gene is rather open-ended. It seems that the association is via the encoded protein to a gene, and its known enzymatic activity. This then links the gene to a metabolite, and known pathways where the enzyme is active. There are many other ways that a gene may have an "association" with a small molecule metabolite however - such as via regulation of the gene or its protein product with small molecules metabolites, literature-based connections, gene expression-metabolite associations etc. It may help the user to know what associations are not a part of the analysis, so the user knows what to expect in terms of how information is retrieved from any given query.
2. Related to the point above, a key linkage of a gene to a metabolite is the encoded protein that has catalytic activity. Although it is possible to get to the information on the protein and its enzymatic activity via the Uniprot link via a couple of clicks by the user, it seems that the Uniprot link could be displayed more prominently on the initial results page after the query is entered. This may be a more efficient way for users to get information on the protein, as a key linkage point to the metabolite/pathway information.
3. It is understood that the "phenotype" query is not available yet. However, it's unclear what type of queries will be available here. Could the authors provide some information on the options for phenotypes that they plan to make available?
4. They authors should be more clear on exactly how many organisms can be queried - at this point it looks like it is a relatively limited number (human, mouse, rat). Are there plans to include any more organisms - such as pathogenic bacteria or other organisms related to human health that might be of interest?

5. In test runs it might be nice to show the number of returned results under "Metabolites", "Reactions", "Pathways" in the first window. Some may be empty and it seems inefficient for the user to have to click through each category only to find out there are not any results?
6. The "clear query" button to start a new query is a bit small - this could be more obvious to the user to clear the form and start again.

Methods

Are the methods appropriate to the aims of the study, are they well described, and are necessary controls included? Choose an item.

Conclusions

Are the conclusions adequately supported by the data shown? Choose an item.

Reporting Standards

Does the manuscript adhere to the journal's guidelines on minimum standards of reporting? Choose an item.

Choose an item.

Statistics

Are you able to assess all statistics in the manuscript, including the appropriateness of statistical tests used? Choose an item.

Quality of Written English

Please indicate the quality of language in the manuscript: Choose an item.

Declaration of Competing Interests

Please complete a declaration of competing interests, considering the following questions:

- Have you in the past five years received reimbursements, fees, funding, or salary from an organisation that may in any way gain or lose financially from the publication of this manuscript, either now or in the future?
- Do you hold any stocks or shares in an organisation that may in any way gain or lose financially from the publication of this manuscript, either now or in the future?
- Do you hold or are you currently applying for any patents relating to the content of the manuscript?
- Have you received reimbursements, fees, funding, or salary from an organization that holds or has applied for patents relating to the content of the manuscript?

- Do you have any other financial competing interests?
- Do you have any non-financial competing interests in relation to this paper?

If you can answer no to all of the above, write 'I declare that I have no competing interests' below. If your reply is yes to any, please give details below.

I declare that I have no competing interests

I agree to the open peer review policy of the journal. I understand that my name will be included on my report to the authors and, if the manuscript is accepted for publication, my named report including any attachments I upload will be posted on the website along with the authors' responses. I agree for my report to be made available under an Open Access Creative Commons CC-BY license (http://creativecommons.org/licenses/by/4.0/). I understand that any comments which I do not wish to be included in my named report can be included as confidential comments to the editors, which will not be published.

Choose an item.

To further support our reviewers, we have joined with Publons, where you can gain additional credit to further highlight your hard work (see: https://publons.com/journal/530/gigascience). On publication of this paper, your review will be automatically added to Publons, you can then choose whether or not to claim your Publons credit. I understand this statement.

Yes Choose an item.
