# Supplementary material for: MetGENE: gene-centric metabolomics information retrieval tool
Source: Gigascience. 2023 Nov 20;12:giad089. doi: 10.1093/gigascience/giad089 (PMC10659118; doi:10.1093/gigascience/giad089)
Supplement: giad089_Reviewer_2_Report_Original_Submission — Yue Wu -- 3/13/2023 [file giad089_reviewer_2_report_original_submission.pdf]

Reviewer Report

Title: MetGENE: Gene-centric Metabolomics Information Retrieval Tool

Version: Original Submission **Date:** 3/13/2023

Reviewer name: Yue Wu

Reviewer Comments to Author:

The authors constructed a system linking pathway databases and metabolomics workbench and built an interface for gene-centric information presentation. On the one hand, there are novelties to connect genes to deposited metabolomics datasets, and data can be interpreted better by merging multiple databases. The API interface can also be possibly useful for future development and batch analysis. On the other hand, the author failed to mention and compare existing tools and the functionality provided by this paper is limited. The writing needs to be improved as well. I suggested a major revision before publication.

Major comments:

1. There are many methods/databases/tools linking genes to pathways, reactions, and metabolites. For example, MetaCyc or HumanCyc provides web and local interfaces, where the search can start from genes, proteins, pathways, or metabolites. Related information can also be easily fetched (e.g., from genes to related reactions or metabolites). Those databases tend to be curated, which increases accuracy but can probably miss possible connections. This manuscript provides database searching that is not curated which can possibly provide more connections. However, such comparisons are not stated in the manuscript. In addition, many of the databases (KEGG, GeneCards) the authors used and mentioned have blocks of genes, pathways, and metabolites. The authors need to explain the significance and improvement of MetGENE upon other databases or curated tools. Is MetGENE going to provide more complete results than any of them? How do MetGENE results trade off between coverage and accuracy compared with others?
2. It seems that the interface with the metabolic workbench is one of the major innovations of this manuscript. The tool and visualization provided for this functionality are however limited. It's mainly through the block of metabolites (METSTAT) and studies, which indeed seem to provide interesting information. Can the author show how MetGENE helps improve users' understanding or efficiency through such integration with a specific biological example? How does integrating results of multiple workbench studies show something not directly visible in one? Some additional functionality in this part can be helpful to the users (e.g., a table of quantification and corresponding metabolites approaches of the study through API OR a summary information table for the study included in the Study page).
3. The presentation of this paper has been mainly qualitative. More quantitative evaluation and presentations can help. For example, what's the size of the search space of genes for MetGENE to have any results? How many reactions, metabolites, pathways, and studies are connected?
4. Only allowing starting from genes seems limited. The search should also be able to start with metabolites or reactions.
5. Even though explained in the method, it's not clear to me what kind of connections between genes,

pathways, reactions, and metabolites the authors used for the results. A supplementary figure showing the logical process of searching starting from one gene can be helpful. Particularly, the user can present a network starting from one specific gene (e.g., PNPLA3) and present how different parts (e.g., metabolites, pathways) are connected in the result.

Minor comments:

6. The pathway result is puzzling as it seems to be links connected to different databases searching for information related to the genes. When I click those links, the information is not solely on pathways. Supplying all the information in a table rather than links can be much easier for the user. The WikiPathways link seems also not working. Similarly, the author can add more information for the tables of reactions (showing the reaction equation itself).

7. It seems white space after the comma when searching for two genes will cause problems.

8. There are many technical details in the result which can be put into methods or supplements. For example, the sentence "Given the three-letter KEGG organism code and the ENTREZ gene ID of the gene, the R KEGGREST API provides a way to access all the information such as pathway IDs, reaction IDs and compound (metabolites) IDs as a data frame objects" can be simplified without mention code length, specific ID, specific API methods, etc. The "MetGENE Query Interface" section of the result also seems too detailed and technical. The result part can be rewritten and reduced to improve accessibility to general readers.

Methods

Are the methods appropriate to the aims of the study, are they well described, and are necessary controls included? Choose an item.

Conclusions

Are the conclusions adequately supported by the data shown? Choose an item.

Reporting Standards

Does the manuscript adhere to the journal's guidelines on minimum standards of reporting? Choose an item.

Choose an item.

Statistics

Are you able to assess all statistics in the manuscript, including the appropriateness of statistical tests used? Choose an item.

Quality of Written English

Please indicate the quality of language in the manuscript: Choose an item.

Declaration of Competing Interests

Please complete a declaration of competing interests, considering the following questions:

- Have you in the past five years received reimbursements, fees, funding, or salary from an organisation that may in any way gain or lose financially from the publication of this manuscript, either now or in the future?
- Do you hold any stocks or shares in an organisation that may in any way gain or lose financially from the publication of this manuscript, either now or in the future?
- Do you hold or are you currently applying for any patents relating to the content of the manuscript?
- Have you received reimbursements, fees, funding, or salary from an organization that holds or has applied for patents relating to the content of the manuscript?
- Do you have any other financial competing interests?
- Do you have any non-financial competing interests in relation to this paper?

If you can answer no to all of the above, write 'I declare that I have no competing interests' below. If your reply is yes to any, please give details below.

I declare that I have no competing interests

I agree to the open peer review policy of the journal. I understand that my name will be included on my report to the authors and, if the manuscript is accepted for publication, my named report including any attachments I upload will be posted on the website along with the authors' responses. I agree for my report to be made available under an Open Access Creative Commons CC-BY license (http://creativecommons.org/licenses/by/4.0/). I understand that any comments which I do not wish to be included in my named report can be included as confidential comments to the editors, which will not be published.

Choose an item.

To further support our reviewers, we have joined with Publons, where you can gain additional credit to further highlight your hard work (see: https://publons.com/journal/530/gigascience). On publication of this paper, your review will be automatically added to Publons, you can then choose whether or not to claim your Publons credit. I understand this statement.

Yes Choose an item.
